# Expanding the Repertoire of Spongian-16-One Derivatives in Australian Nudibranchs of the Genus *Goniobranchus* and Evaluation of Their Anatomical Distribution

**DOI:** 10.3390/md19120680

**Published:** 2021-11-29

**Authors:** Louise C. Forster, Jack K. Clegg, Karen L. Cheney, Mary J. Garson

**Affiliations:** 1 School of Chemistry and Molecular Biosciences, The University of Queensland, St. Lucia, QLD 4072, Australia; l.forster1@uq.edu.au (L.C.F.); j.clegg@uq.edu.au (J.K.C.); 2 School of Biological Sciences, The University of Queensland, St. Lucia, QLD 4072, Australia; k.cheney@uq.edu.au

**Keywords:** diterpenes, nudibranch, Chromodorididae, *Goniobranchus aureopurpureus*, X-ray crystallography

## Abstract

Extracts of the mantle and viscera of the Indo-Pacific nudibranchs *Goniobranchus aureopurpureus* and *Goniobranchus* sp. 1 afforded 11 new diterpenoids (**1**–**11**), all of which possess a tetracyclic spongian-16-one scaffold with extensive oxidation at C-6, C-7, C-11, C-12, C-13, and/or C-20. The structures and relative configuration were investigated by NMR experiments, while X-ray crystallography provided the absolute configuration of **1**, including a 2′*S* configuration for the 2-methylbutanoate substituent located at C-7. Dissection of animal tissue revealed that the mantle and viscera tissues differed in their metabolite composition with diterpenes **1**–**11** present in the mantle tissue of the two nudibranch species.

## 1. Introduction

Spongian diterpenes are bioactive natural products isolated from sponges of the orders *Dictyoceratida* and *Dendroceratida*, and nudibranchs (shell-less mollusc) predators [1,2]. The first example of the spongian diterpene scaffold was isoagatholactone, isolated from the Mediterranean sponge *Spongia officinalis* by Cimino et al., and with the structure and absolute configuration established through chemical correlation with grindelic acid [3]. The metabolites spongian-16-one and spongian-15,16-dione were isolated from the New Zealand sponge *Dictyodendrilla cavernosa* [4]. The structure of spongian-16-one was determined through exhaustive nuclear magnetic resonance (NMR) studies carried out independently by two groups, those of Kernan et al. [4] and Hambley et al. [5]. Spongian-16-one has also been isolated from several nudibranch species, including *Chromodoris obsoleta* and exhibits moderate anti-neoplastic activity against L1210 (IC_50_ = 5.0 µg mL^−1^) and KB (IC_50_ = 9.2 µg mL^−1^) cell lines [6]. In this group of tetracyclic diterpenes, further oxidation is commonly seen at C-7, C-11, C-12, C-13, C-15, C-17, and/or C-20 [7].

The current study, which forms part of our comprehensive study on nudibranchs of the genus *Goniobranchus* [7,8,9,10,11,12,13,14,15,16,17,18,19], represents the first chemical report on the secondary metabolite profile of the two nudibranch species, *Goniobranchus aureopurpureus* and *Goniobranchus sp.* 1 [20], each reassigned from an earlier taxonomic classification as *Chromodoris* species [21]. The structures and relative configuration of eleven isolated diterpene metabolites were determined by analysis of their two-dimensional NMR spectra as well as where applicable by X-ray crystallography to determine the absolute configuration. Our study also investigated the anatomical location of the terpenes and compared this with the distribution of metabolites in other *Goniobranchus* species.

## 2. Results and Discussions

### 2.1. Diterpenes from Goniobranchus aureopurpureus

**Figure 1:**
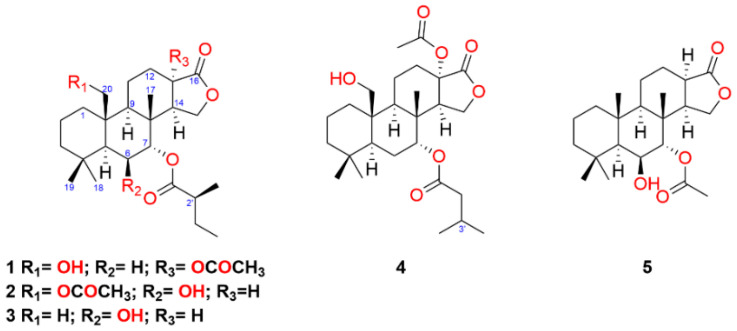
Structures of diterpenes **1**–**5**.

Six specimens of *G. aureopurpureus* were collected from Nelson Bay (New South Wales, Australia) in March 2016. Specimens were dissected into their mantle and viscera and each body part was finely chopped, extracted with acetone and the extract concentrated under vacuum. The aqueous residues were partitioned with diethyl ether to yield an orange oil from the mantles and a green oil from the visceras. The individual mantle extracts were combined, as were the individual viscera extracts prior to fractionation by silica-flash chromatography. Subsequent normal phase high-performance liquid chromatography (NP-HPLC) yielded terpenes **1**–**5**. The known compounds macfarlandin E [22], aplyviolene [23], polyrhaphin B [24], shahamin C [25], and secoshahamin [26] were also isolated from the mantle extract, while the viscera provided luffarin-X [27], spongian-16-one [4,5], 7α-acetoxyspongian-16-one [28], polyrhaphin A [24], 15,16-diacetoxyshahamin B [25], and 12-desacetoxypolyrhaphin A [29]. The known terpenes spongian-16-one, 7α-acetoxyspongian-16-one, macfarlandin E, aplyviolene, polyrhaphin B, and secoshahamin were isolated from both tissues. The new spongian diterpenes (**1**–**5**) show varying levels of oxidation, particularly at positions C-6, C-7, C-13 and C-20 (Figure 1).

**Figure 2:**
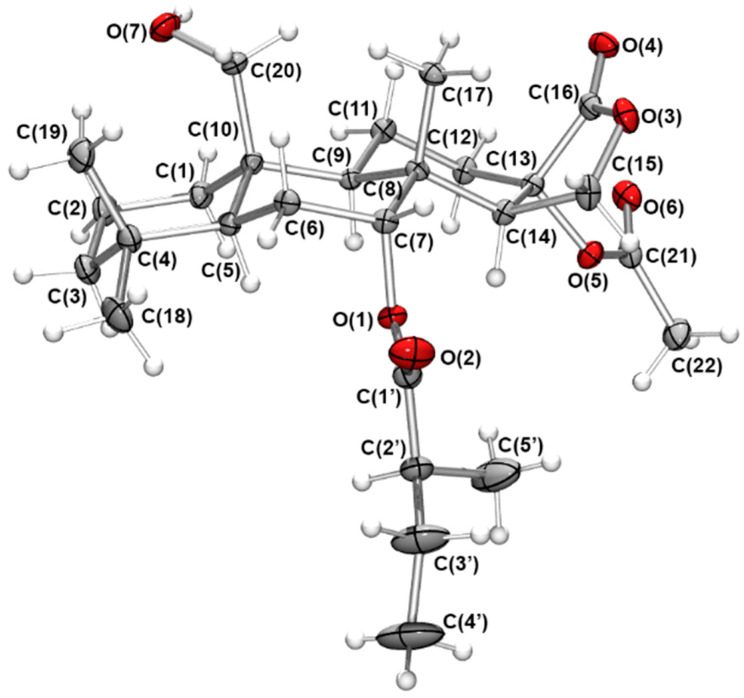
Oak Ridge Thermal Ellipsoid Plot (ORTEP) [32] representation of the crystal structure of (5*S*, 7*R*, 8*R*, 9*R*, 10*R*, 13*S*, 14*R,* 2′*S*)*-*13-acetoxy-20-hydroxy-7α-oxyspongian-16-one-7α-(2-methyl)-butanoate (**1**) shown with 30% probability ellipsoids.

Metabolite **1** was isolated as a colourless oil and displayed a sodiated ion at *m/z* 501.2829 [M + Na]^+^ from high-resolution electrospray ionisation mass spectrometry (HRESIMS) for C_27_H_42_O_7_. These data indicated an additional seven carbons and five oxygens when compared to spongian-16-one. The ^1^H and ^13^C NMR spectroscopic data (Table 1 and Table 2; see Supplementary Material) also supported a substituted spongian-16-one skeleton [4,5] but with a methyl singlet at δ_H_ 2.04 and a triplet signal at δ_H_ 4.85, suggesting additional functionality, namely an acetate group and substitution at C-7, respectively. Doublet and triplet signals at δ_H_ 1.15 (*J* = 6.9 Hz) and δ_H_ 0.89 (*J* = 7.4 Hz), respectively, were attributed to the methyl groups of a 2-methylbutanoate ester, accounting for the remaining five carbon atoms, with gCOSY and HSQC data further validating the CH_3_(CH_3_CH_2_)CH- substructure. Signals for three ester carbonyls (δ_C_ 169.7, 174.0, and 175.4) validated six of the oxygen atoms present in the molecular formula. HMBC correlations from the signals at δ_H_ 4.85 (H-7, t, *J* = 2.8 Hz) and 1.15 (7-OCOCH*CH_3_*CH_2_CH_3_, d) to the signal at δ_C_ 175.4 confirmed the 2-methylbutanoate group at C-7. Noting that the *J* values of H-14 (δ_H_ 2.92, dd, *J* = 1.5, 6.5 Hz) for **1** were different from those observed in spongian-16-one (Kernan et al.: 2.07, dd, *J* = 5.0, 8.0 Hz; Hambley et al.: 2.09, dd, *J* = 5.4, 8.0 Hz ) [4,5], HMBC correlations from H-12eq (δ_H_ 2.28), H-14 (δ_H_ 2.92) and H-15ax (δ_H_ 4.20), as well as the signals at δ_H_ 2.04 (13-OCOCH_3_) and 2.92 (H-14) to the signal at δ_C_ 81.1 (C-13), located the acetate group at C-13. The signal for Me-20 of spongian-16-one [4,5] was replaced by signals for an oxymethylene H_2_-20 (δ_H_ 4.05 and δ_H_ 3.92) in **1**. These proton chemical shifts were inconsistent with esterification at C-20 [7,30]; therefore, a hydroxy group was located at C-20, identifying the final oxygen atom. NOESY correlations observed between H-7/Me-17 and H_2_-20/Me-17 confirmed the same relative configuration as spongian-16-one; however, the configuration of the acetate at C-13 and the 2′-methyl in the 2-methylbutanoate substituent could not be determined by NMR methods. Metabolite **1** was crystallized from 10% EtOAc/hexanes, producing small needle-shaped crystals which were suitable for diffraction. The resulting crystal structure obtained established the overall relative configuration. The absolute configuration was assigned as 5*S*, 7*R*, 8*R*, 9*R*, 10*R*, 13*S*, 14*R,* 2′*S*; within naturally-occurring 2-alkylalkanoic acid derivatives, the 2′*S* configuration is favoured [31]. In **1**, the cyclohexane rings A, B, and C each adopt a chair conformation. As a result, adjacent molecules interact through hydrogen bonds O(7)H•••O(4) = 2.17 Å, 167°, resulting in the formation of an undulating one-dimensional polymeric chain that extends down parallel to the crystallographic *a*-axis (Figure 2). The name of compound **1** was assigned as (-)-13-acetoxy-20-hydroxy-7α-oxyspongian-16-one-7α-(2-methyl)-butanoate.

Diterpene **2** was isolated as a colourless oil and found to have the same C_27_H_42_O_7_ molecular formula as **1** inferred from HRESIMS (*m/z* 501.2824 [M + Na]^+^). Examination of the ^1^H and ^13^C NMR spectroscopic data (Table 1 and Table 2) revealed similar signals to those of **1**, including a methyl doublet at δ_H_ 1.15 (*J* = 6.9 Hz) and a methyl triplet at δ_H_ 0.91 (*J* = 7.4 Hz) for the methyl groups of a 2-methylbutanoate ester; there was also an acetate methyl singlet at δ_H_ 2.03. HMBC correlations from the signals at δ_H_ 4.87 (H-7, d, *J* = 3.2 Hz) and 1.15 (7-OCOCH*CH_3_*CH_2_CH_3_, d) to the signal at δ_C_ 174.9 confirmed the 2-methylbutanoate group at C-7. The configuration of the 2′-methyl in the ester sidechain could not be established further, owing to the small sample size, but was selected as identical to that in **1** on biogenetic considerations. HMBC correlations from H-20a (δ_H_ 4.79) and H-20b (δ_H_ 4.73) to the signal at δ_C_ 170.6 confirmed the position of the acetoxy group at C-20; there were NOESY correlations between H_2_-20/Me-17 and H-20b/Me-19. The doublet appearance of H-7 (*J* = 3.2 Hz) was initially considered consistent with an equatorial OH group at C-6; however, the signal for H-5 was a broadened singlet rather than the doublet with a large *J* value anticipated if H-6 was axial, (cf. aplyroseol-19 from *Chromodoris reticulata* [33]). The NOESY correlation between H-6/Me-18 supported an equatorial H-6, while the absence of an NOE between H-6 and Me-17, although not diagnostic, was also consistent with the changed configuration at C-6 compared to that in aplyroseol-19 [33]. The NOESY correlation between H-7/Me-17 placed the C-7 ester substituent on the opposite face to Me-17. Compound **2** was assigned the systematic name (-)-20-acetoxy-6β-hydroxy-7α-oxyspongian-16-one-7α-(2-methyl)-butanoate.

Metabolite **3**, also isolated as a colourless oil, displayed an adduct ion at *m/z* 443.2779 [M + Na]^+^ in HRESIMS analysis, which established the molecular formula as C_25_H_40_O_5_ with an additional five carbons and three oxygens compared with spongian-16-one. Due to the small sample quantity (<0.1 mg), a Shigemi tube was employed to increase the sensitivity of NMR signal detection [34]. The spectroscopic data again revealed a 2-methylbutanoate moiety, located at C-7 from the identical HMBC correlations for H-7 to those in **1** and **2**. NOESY data could not be obtained, but the similar appearance of the signals for H-6 (δ_H_ 4.18, br s) and H-7 (δ_H_ 4.84, *J* = 2.6 Hz) compared to **2** established the axial hydroxy group at C-6 and the equatorial ester group at C-7. The 7.8 Hz coupling between H-13 and H-14 assigned the *cis* C/D ring junction. The name of compound **3** was assigned as (-)-6β-hydroxy-7α-oxyspongian-16-one-7α-(2-methyl)-butanoate.

Diterpene **4**, a colourless oil, exhibited an adduct ion at *m/z* 501.2831 [M + Na]^+^ in the HRESIMS, corresponding to a molecular formula of C_27_H_42_O_7_, which was the same molecular formula observed for **1** and **2**. The ^1^H and ^13^C NMR spectroscopic data revealed an acetate methyl singlet at δ_H_ 2.04 as well as oxymethylene signals at δ_H_ 4.04 (d, *J* = 11.8 Hz) and 3.91 (d, *J* = 11.8 Hz) for H_2_-20, similar to comparable signals for **1**, and suggesting a C-20 hydroxy group. The major difference compared to the data for **1** was the presence of two methyl doublets at δ_H_ 0.95 (d, *J* = 6.6 Hz) and 0.96 (d, *J* = 6.6 Hz) suggesting a 3-methylbutanoate substituent. There were gCOSY correlations from the H-3′ methine (δ_H_ 2.11, m) to both Me-4′ and Me-5′ and H_2_-2′ (δ_H_ 2.22, m, 2H). HMBC correlations from the signals at δ_H_ 4.86 (H-7, t, *J* = 2.6 Hz), 2.11 (7-OCOCH_2_*CH*(CH_3_)_2_, m, H-3′) and the methylene signals at δ_H_ 2.22 (7-OCO*CH_2_*CH(CH_3_)_2_, m, H_2_-2′) to the carbon at δ_C_ 171.9 confirmed the 3-methylbutanoate group was attached at C-7. These ^1^H chemical shifts and HMBC correlations were comparable to those of 7α-11α-dioxyspongian-16-one-7α-isopentanoate-11α-propionate [7]. The similarity of the signal pattern and chemical shift of H-14 (δ_H_ 2.91, dd, *J* = 1.2, 6.3 Hz) to that in **1**, together with HMBC correlations from the signals at δ_H_ 2.04 (13-OCOCH_3_) and 2.91 (H-14) to the carbon at δ_C_ 81.0 (C-13) confirmed an acetate group at C-13. NOESY correlations determined the relative configuration of C-7 and C-10 to be identical to those of **1** and **2**. X-ray studies (See Supplementary Materials) supported the configuration of C-13 to be the same as in **1**. Compound **4** was assigned the name (-)-13-acetoxy-20-hydroxy-7α-oxyspongian-16-one-7α-(3-methyl)-butanoate.

Diterpene **5** was isolated as a colourless oil and displayed a sodiated molecular ion peak by HRESIMS at *m/z* 401.2293 [M + Na]^+^, corresponding to a molecular formula of C_22_H_34_O_5_. The ^1^H and ^13^C NMR spectroscopic data (Table 1 and Table 2) showed an acetoxy methyl singlet at δ_H_ 2.09 and associated carbonyl signal at δ_C_ 169.7, comparable to those in the NMR data of 7α-acetoxyspongian-16-one [28]. HMBC correlations from the signals at δ_H_ 2.09 and 4.84 (H-7, d, *J* = 3.1 Hz) to the carbonyl at δ_C_ 169.7 confirmed the position of the acetoxy group at C-7. The doublet appearance of H-7 suggested hydroxy substitution at C-6. The relative configuration of **5** was identical to that of **2** from the NOESY correlations between H-6/Me-18, H-7/Me-17, H-5/H-9, and H-9/H-14. Compound **5** was assigned the systematic name (-)-7α-acetoxy-6β-hydroxyspongian-16-one.

### 2.2. Diterpenes from Goniobranchus sp. 1

**Figure 3:**
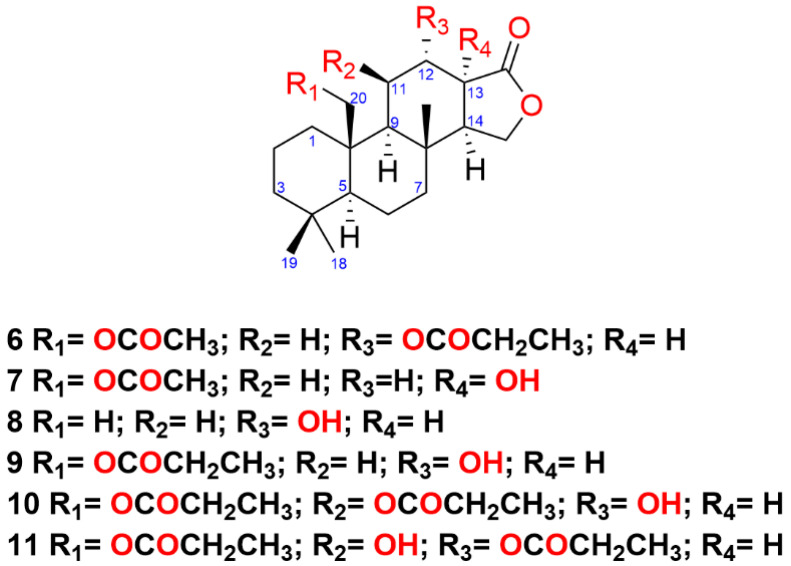
Chemical structures of **6–11**.

Three specimens of *Goniobranchus* sp. 1 were collected from Mudjimba and Gneerings Reefs, South East Queensland, Australia. The extraction and chemical profile of the metabolites from the mantle and viscera tissue were carried out based on the previously described procedures. A total of fifteen spongian diterpene metabolites were isolated from *Goniobranchus* sp 1, including the new spongian-16-one analogues **6–11**. From the mantle isoagatholactone [3], 12α-acetoxyspongian-16-one [30], 20-acetoxyspongian-16-one [30], 20-oxyspongian-16-one-propionate [30], 12α,20-dioxyspongian-16-one-dipropionate [30], 12α,20-diacetoxyspongian-16-one [7], 12α-acetoxy-20-oxyspongian-16-one-20-propionate [7], 20-acetoxy-12α-oxyspongian-16-one-12α-propionate (**6**), 20-acetoxy-13-hydroxyspongian-16-one (**7**), 12-hydroxyspongian-16-one (**8**), 12-hydroxy-20-oxyspongian-16-one-20-propionate (**9**), 12-hydroxy-11,20-dioxyspongian-16-one-11,20-dipropionate (**10**), and 11-hydroxy-12,20-dioxyspongian-16-one-12,20-dipropionate (**11**) were also isolated, while the viscera contained spongian-16-one [4,5] and 7α-acetoxyspongian-16-one [28]. The known metabolites isoagatholactone, 12α-acetoxyspongian-16-one [30], 20-acetoxyspongian-16-one [30], and 12α-acetoxy,20-oxyspongian-16-one-20-propionate [7] were isolated from both tissues. The new compounds (**6–11**) demonstrate a high level of oxidation, in particular at positions C-11, C-12, C-13 and/or C-20 (Figure 3).

Diterpene **6** was obtained as a colourless oil from NP-HPLC and exhibited a sodiated molecular ion peak in the HRESIMS at *m/z* 457.2566 [M + Na]^+^ (C_25_H_38_O_6_). The ^1^H and ^13^C NMR spectroscopic data (Table 3 and Table 4) indicated an acetate group (δ_H_ 2.03, δ_C_ 21.2, 170.8) while a quartet (2H) at δ_H_ 2.33, a triplet at δ_H_ 1.16 and a carbonyl resonance at δ_C_ 173.0 were assigned to a propionate group. The NMR data of **6** were found to be similar to those of 12α-acetoxy-20-oxyspongian-16-one-20-propionate; however, there were some obvious differences in the location of substituents [7]. Oxymethylene signals at δ_H_ 4.56 (d) and 4.13 (m) corresponded to those of H_2_-20 in 20-acetoxyspongian-16-one [30]. These two signals, plus the acetate methyl signal at δ_H_ 2.03 (s), all correlated to the carbon signal at δ_C_ 170.8 and C-10 (δ_C_ 39.8), confirming the position of an acetate group at C-20. The chemical shift values, in particular those of H_2_-11 and H-13, as well as C-12, were comparable to those in the ^1^H and ^13^C NMR spectra of 12α,20-dioxyspongian-16-one-dipropionate [30] thereby establishing the propionate group at C-12. NOESY correlations between H-5/H-9, H-9/H-14, Hb-20/Me-17, H-20a/Me-19 and H-12/Me-17 placed H-12 and H_2_-20 on the same face as Me-17. Compound **6** was named (-)-20-acetoxy-12α-oxyspongian-16-one-12α-propionate.

Metabolite **7**, which was isolated as a colourless oil, exhibited an adduct ion at *m/z* 401.2291 [M + Na]^+^ from HRESIMS, which corresponded to the same molecular formula as **5**. The ^1^H and ^13^C NMR spectroscopic data again revealed an acetate group (δ_H_ 2.02, δ_C_ 21.2, 170.8) while oxymethylene signals at δ_H_ 4.55 (d, *J* = 13.1 Hz) and 4.14 (d, *J* = 13.1 Hz) were consistent with those of H_2_-20 in **6**. HMBC correlations from the signals at δ_H_ 4.55 and 4.14 as well as from δ_H_ 2.02 to the carbon at δ_C_ 170.8 and to C-10 (δ_C_ 40.4) confirmed the position of the acetate group at C-20. NOE correlations between H-20a/Me-19 and H-20b/Me-17 again placed the C-20 acetate on the same face as Me-17. The upfield chemical shift for H-14 (δ_H_ 1.94, dd, *J* = 5.6, 7.8 Hz), together with HMBC correlations from signals at δ_H_ 1.94 (H-14) and 4.13 (H-15ax) to the quaternary carbon at δ_C_ 83.7 (C-13), confirmed a hydroxy group at C-13. The configuration at C-13 was not explored further, owing to the small quantity (0.2 mg) of the sample, and was provisionally assigned by comparison with **1** and **3**. NOESY correlations between H-5/H-9 and H-9/H-14 confirmed the remaining stereochemistry. Compound **7** was assigned the systematic name (-)-20-acetoxy-13-hydroxyspongian-16-one.

Metabolite **8** was isolated as a colourless oil and displayed a sodiated ion at *m/z* 343.2245 [M + Na]^+^ from HRESIMS for C_20_H_32_O_3_ suggesting an extra hydroxy group compared to spongian-16-one. gCOSY correlations from the oxygenated methine proton signal at δ_H_ 4.52 (H-12, br s) to δ_H_ 1.63 (H_2_-11) and 2.66 (H-13) established the hydroxy group at the C-12 position, further confirmed by HMBC correlations. NOESY correlations observed between H-5/H-9, H-9/H-14, and H-12/Me-17 confirmed the overall stereochemistry. Compound **8** was named as (-)-12α-hydroxyspongian-16-one.

Diterpene **9** was isolated as a colourless oil and produced an adduct ion at *m/z* 415.2458 [M+Na]^+^ from HRESIMS for C_23_H_36_O_5_. The ^1^H NMR spectrum indicated a quartet (2H) at δ_H_ 2.31 and a triplet at δ_H_ 1.13, corresponding to propionate methylene and methyl signals. Oxymethylene signals at δ_H_ 4.59 (d, *J* = 12.1 Hz) and 4.17 (d, *J* = 12.1 Hz) corresponded to those of H_2_-20 in 20-oxyspongian-16-one-propionate [30]. HMBC correlations from the signals at δ_H_ 4.59 and 4.17, as well as from δ_H_ 2.31 (2H) and 1.13 to the carbon at δ_C_ 174.5 and C-10 (δ_C_ 40.2) confirmed the propionate group at C-20. The signals at δ_H_ 4.49 (H-12, br s) and 2.65 (H-13) were comparable to those in the ^1^H NMR spectrum of **8**, positioning a hydroxy group at C-12. NOE correlations between Me-17, H-12 and H_2_-20 positioned the propionate group on the same face and the 12-OH on the opposite face to Me-17. The name of compound **9** was assigned as (-)-12α-hydroxy-20-oxyspongian-16-one-20-propionate.

Metabolite **10**, a colourless oil, exhibited an adduct ion at *m/z* 487.2668 [M + Na]^+^ in the HRESIMS, corresponding to a molecular formula of C_26_H_40_O_7_, and 16 mass units larger than that of 12α, 20-dioxyspongian-16-one-dipropionate [30]. The ^1^H NMR spectrum revealed two multiplets at δ_H_ 2.34 (2H) and 2.46 (2H) and two methyl triplets at δ_H_ 1.15 (*J* = 7.7 Hz) and 1.18 (*J* = 7.7 Hz). The addition of two ester carbonyls (δ_C_ 173.6 and 174.2) and the lactone carbonyl (δ_C_ 180.3) located six of the oxygen atoms, with the seventh oxygen atom inferred to be an additional hydroxy group. Oxymethylene signals at δ_H_ 4.74 (d, *J* = 12.0 Hz) and 3.96 (dd, *J* = 1.9, 12.0 Hz) corresponded to those of H_2_-20 in **9** and 20-oxyspongian-16-one-propionate [30]. HMBC correlations from the signals at δ_H_ 4.74 and 3.96 as well as from the signals at δ_H_ 2.46 (2H) and 1.18 to the carbon at δ_C_ 174.2 and to C-10 (δ_C_ 41.2) confirmed a propionate group at C-20. The multiplicity of the H-9 signal at δ_H_ 1.35 was a doublet rather than the doublet of doublets observed for **8**. HMBC correlations from H-11 (δ_H_ 5.95) and 11-OCOCH_2_CH_3_ (δ_H_ 2.34 and 1.15) to the propionate carbonyl at δ_C_ 173.6 located the second propionate group at C-11. gCOSY correlations from H-11 and H-13 (δ_H_ 2.84) to H-12 (δ_H_ 2.79) confirmed the hydroxy group at C-12. The 9.4 Hz coupling between H-12ax and H-13 established a boat conformation for ring C [7]. NOESY correlations observed between H-5/H-9, H-9/H-14, H-20b/Me-17, and H-12/Me-17 confirmed the overall stereochemistry. Compound **10** was named systematically as (-)-12α-hydroxy-11β,20-dioxyspongian-16-one-11β,20-dipropionate.

The spongian-16-one analogue **11** was isolated as a colourless oil and produced an adduct ion at *m/z* 487.2667 [M + Na]^+^, giving the same molecular formula as **10**, implying two propionate groups and a hydroxy group. The ^1^H and ^13^C NMR spectroscopic data revealed signals for two propionate groups. Similar to **10**, two ester carbonyls (δ_C_ 172.6 and 175.5) and a lactone carbonyl (δ_C_ 178.2) were identified. HMBC correlations from the signals at δ_H_ 4.61 (d, *J* = 12.2 Hz) and 4.02 (dd, *J* = 1.8, 12.2 Hz) as well as from δ_H_ 2.50, 2.45 and 1.12 to the carbon at δ_C_ 175.5 and C-10 (δ_C_ 40.7) confirmed a propionate group at C-20. HMBC correlations from the signal at δ_H_ 5.54 (H-12, dd, *J* = 3.0, 9.2 Hz) to the signal at δ_C_ 172.6 located the second propionate group at C-12. Lastly, the occurrence of a signal at δ_H_ 1.34 (d, *J* = 3.0 Hz) for H-9, together with gCOSY correlations from H-12 and H-9 to H-11 (δ_H_ 4.46), established a hydroxy group at C-11. The 9.2 Hz coupling between H-12ax and H-13 again established a boat conformation for ring C [7]. The NOESY correlations observed between H-5/H-9, H-9/H-14, H-20b/Me-17, and H-12/Me-17 confirmed the overall stereochemistry. Compound **11** was named as (-)-11β-hydroxy-12α,20-dioxyspongian-16-one-12α,20-dipropionate.

### 2.3. Anatomical Distribution of Metabolites

Comparison of individual body parts by ^1^H NMR spectroscopy, together with subsequent isolation work, revealed that new metabolites **1**–**5** were solely isolated from the mantle tissue of *G. aureopurpureus*. Likewise, new metabolites **6**–**11** were isolated only from the mantle tissue of *Goniobranchus sp.* 1. We also found that both species had more chemical diversity of metabolites in the mantle relative to the viscera. A full list of metabolites found in each body part is provided in the Supplementary Material. This pattern of anatomical distribution matches that of four *Goniobranchus* species that we previously studied (*G. tinctorius*, *G. tasmaniensis*, *G. collingwoodi,* and *G. splendidus*) [7,35]. These species may accumulate compounds in the mantle as they feed on a variety of sponge species with different chemistry. Compounds in the mantle are thought to be used for defensive purposes, and complex defensive mixtures may provide protection from a range of predators [10]. In contrast, we previously found two species (*G. hunterae* and *G. verrieri)* with the same metabolites in the mantle and viscera tissue, and one species (*G. daphne*) with fewer compounds in the mantle compared to the viscera [35].

## 3. Conclusions

In conclusion, the isolation work was conducted on two *Goniobranchu*s species and afforded eleven new spongian diterpenes with oxidation at various positions, such as C-6, C-7, C-11, C-12, C-13, and/or C-20. The X-ray structure of **1** provided insight into the absolute configuration of the parent spongian-16-one [4,5]. Many of these highly oxygenated spongian diterpenes were only isolated from the mantle tissue, where they may play a role in deterring predators. 

## 4. Materials and Methods 

### 4.1. General Experimental Procedure

Specific rotations were measured at 23 °C on a Jasco P-2000 polarimeter for solutions in CHCl_3_ using a 1-millilitre cell (10-centimetre path length). NMR spectroscopic data were recorded on a Bruker Avance 500 spectrometer using a 5-millimetre SEI probe or a Bruker Avance DRX 700 MHz spectrometer with a 5-millimetre TXI Zgrad probe for solutions in CDCl_3_ at 298K. Heteronuclear single quantum correlation (HSQC) and heteronuclear multiple bond correlation (HMBC) data were acquired using a ^1^*J*_C-H_ of 145 Hz, while HMBC spectra were acquired using ^n^*J*_C-H_ of 8 Hz. Positive and negative ion electrospray mass spectra were determined using either a Bruker Esquire HCT 3D ion trap instrument for low-resolution electrospray ionization mass spectrometry (LRESIMS) or a MicrOTOF-Q or an Orbitrap Elite instrument for high-resolution electrospray ionization mass spectrometry (HRESIMS) with MeOH as solvent. Normal–phase high-performance liquid chromatography (NP-HPLC) was undertaken using a Waters 515 pump connected to a Gilson 132 series refractive index detector with a Phenomenex Luna (5 μm, 10 × 250 mm) column, using isocratic elution conditions at flow rates between 1–2 mL/ min. Silica gel 60 G and silica TLC plates F_254_ were purchased from Merck. Solvents were either distilled or were HPLC grade. 

### 4.2. Biological Material 

Six individuals of *Goniobranchus aureopurpureus* were collected from Nelson Bay (#1469-1474), New South Wales in March 2016. Three individuals of *Goniobranchus (Chromodoris)* sp. 1 were collected from Mudjimba (#1368 and #1563) and Gneerings Reefs (#1575) (Mooloolaba, Queensland) in October 2015 and October 2016. All collections were stored in individual containers at −20 °C until dissection into mantle and gut prior to extraction.

### 4.3. Extraction and Purification 

The mantle and viscera tissue of each specimen of *G. aureopurpureus* and *G. sp 1* were extracted in acetone (3 × 2 mL) and sonicated (5 min) separately. The extracts were reduced to aqueous suspensions, extracted with Et_2_O (3 × 3 mL), dried over anhydrous Na_2_SO_4_, and concentrated under N_2_ to give an orange oil (mantle tissue) or a green oil (viscera). The ^1^H NMR profile of the mantle and viscera extracts were compared between the specimens of each species and showed similar chemistry; for *G. aureopurpureus* (specimens #1469-1474) the mantle extracts were combined (51.9 mg) and the viscera extracts combined (56.1 mg) to produce two extracts. For *G. sp* 1 (specimens #1563, 1368 and 1575), the mantle extracts were combined (96.8 mg), as were the viscera extracts (71.2 mg). The extracts were further separated by NP-flash column chromatography with a stepwise solvent gradient from 100% hexanes to 100% MeOH.

Mantle fractions of *G. aureopurpureus* were further separated by NP-HPLC (25–30% EtOAc in hexanes) to yield 15-desacetoxy-12-acetoxydendrillolide A (0.4 mg), spongian-16-one (2.5 mg), macfarlandin E (1.8 mg), aplyviolene (1.6 mg), 7α-acetoxyspongian-16-one (0.6 mg), polyrhaphin B (0.1 mg), secoshahamin (0.1 mg), shahamin C (0.1 mg), 7α-acetoxy-6β-hydroxyspongian-16-one (**5**: 0.22 mg), 13-acetoxy-20-hydroxy-7α-oxyspongian-16-one-7α-(2-methyl)-butanoate (**1**: 1.2 mg), 6β-hydroxy-7α-oxyspongian-16-one-7α-(2-methyl)-butanoate (**3**: 0.06 mg), 20-acetoxy-6β-hydroxy-7α-oxyspongian-16-one-7α-(2-methyl)-butanoate (**2**: 0.07 mg), and 13-acetoxy-20-hydroxy-7α-oxyspongian-16-one-7α-(3-methyl)-butanoate (**4**: 0.8 mg). Viscera fractions of *G. aureopurpureus* were further separated by NP-HPLC (25-30% EtOAc in hexanes) to yield luffarin-X (0.22 mg), spongian-16-one (0.43 mg) macfarlandin E (0.46 mg), polyrhaphin B (0.1 mg), secoshahamin (0.12 mg), polyrhaphin A (0.32 mg), 12-desacetoxypolyrhaphin A (0.14 mg), 15,16-diacetoxyshahamin B (0.14 mg), aplyviolene (0.79 mg), and 7α-acetoxyspongian-16-one (0.44 mg).

The NP-flash column chromatography mantle fractions of *Goniobranchus sp 1* were separated by NP-HPLC (30% EtOAc in hexanes) to provide isoagatholactone (0.5 mg), 12α-acetoxyspongian-16-one (0.23 mg), 20-acetoxyspongian-16-one (7.94 mg), 20-oxyspongian-16-one-propionate (0.35 mg), 12α,20-diacetoxyspongian-16-one (0.43 mg), 12α,20-dioxyspongian-16-one-dipropionate (1.30 mg), 12α-acetoxy-20-oxyspongian-16-one-20-propionate (0.28 mg), 20-acetoxy-12α-oxyspongian-16-one-12α-propionate (**6**: 0.21 mg), and 20-acetoxy-13-hydroxyspongian-16-one (**7**: 0.17 mg). Mantle fractions eluting from DCM/EtOAc 4:1 and 1:1 were separated by NP-HPLC (30% EtOAc in hexanes) to yield 12-hydroxyspongian-16-one (**8**: 0.08 mg), 12-hydroxy-20-oxyspongian-16-one-20-propionate (**9**: 0.97 mg), 12-hydroxy-11,20-dioxyspongian-16-one-11,20-dipropionate (**10**: 0.17 mg), and 11-hydroxy-12,20-dioxyspongian-16-one-12,20-dipropionate (**11**: 0.11 mg). Viscera fractions eluting from hexanes/DCM (1:1 and 1:4), 100% DCM and DCM/EtOAc 4:1 were combined and separated by NP-HPLC (30% EtOAc in hexanes), providing isoagatholactone (0.16 mg), spongian-16-one (2.59 mg), 7α-acetoxyspongian-16-one (0.57 mg), 12α-acetoxyspongian-16-one (1.01 mg), 20-acetoxyspongian-16-one (0.30 mg), and 12α,20-diacetoxyspongian-16-one (0.83 mg).

(–)-13-Acetoxy-20-hydroxy-7α-oxyspongian-16-one-7α-(2-methyl)-butanoate (**1**): colourless oil (1.2 mg); ${\left[\alpha \right]}_{D}^{21}$ –12 (*c* 0.12, CHCl_3_); ^1^H NMR and ^13^C NMR (CDCl_3_, 700 MHz), Table 1 and Table 2; HRESIMS *m/z* 501.2829 [M + Na]^+^ (calculated for C_27_H_42_NaO_7_, 501.2823). 

(–)-20-Acetoxy-6β-hydroxy-7α-oxyspongian-16-one-7α-(2-methyl)-butanoate (**2**): colourless oil (0.07 mg); ${\left[\alpha \right]}_{D}^{21}$ –71 (*c* 0.007, CHCl_3_); ^1^H NMR and ^13^C NMR (CDCl_3_, 700 MHz), Table 1 and Table 2; HRESIMS *m/z* 501.2824 [M + Na]^+^ (calculated for C_27_H_42_NaO_7_, 501.2823). 

(–)-6β-Hydroxy-7α-oxyspongian-16-one-7α-(2-methyl)-butanoate (**3**): colourless oil (0.06 mg); ${\left[\alpha \right]}_{D}^{21}$ –167 (*c* 0.006, CHCl_3_); ^1^H NMR and ^13^C NMR (CDCl_3_, 700 MHz), Table 1 and Table 2; HRESIMS *m/z* 443.2779 [M + Na]^+^ (calculated for C_25_H_40_NaO_5_, 443.2768). 

(–)-13-Acetoxy-20-hydroxy-7α-oxyspongian-16-one-7α-(3-methyl)-butanoate (**4**): colourless oil (0.8 mg); ${\left[\alpha \right]}_{D}^{21}$ –19 (*c* 0.08, CHCl_3_); ^1^H NMR and ^13^C NMR (CDCl_3_, 700 MHz), Table 1 and Table 2; HRESIMS *m/z* 501.2831 [M + Na]^+^ (calculated for C_27_H_42_NaO_7_, 501.2823). 

(–)-7α-Acetoxy-6β-hydroxyspongian-16-one (**5**): colourless oil (0.22 mg); ${\left[\alpha \right]}_{D}^{21}$ –65 (*c* 0.022, CHCl_3_); ^1^H NMR and ^13^C NMR (CDCl_3_, 700 MHz), Table 1 and Table 2; HRESIMS *m/z* 401.2293 [M + Na]^+^ (calculated for C_22_H_34_NaO_5_, 401.2298). 

(–)-20-Acetoxy-12α-oxyspongian-16-one-12α-propionate (**6**): colourless oil (0.21 mg); ${\left[\alpha \right]}_{D}^{21}$ –29 (*c* 0.021, CHCl_3_); ^1^H NMR and ^13^C NMR (CDCl_3_, 700 MHz), Table 3 and Table 4; HRESIMS *m/z* 457.2566 [M + Na]^+^ (calculated for C_25_H_38_NaO_6_, 457.2561). 

(–)-20-Acetoxy-13-hydroxyspongian-16-one (**7**): colourless oil (0.17 mg); ${\left[\alpha \right]}_{D}^{21}$ –22 (*c* 0.017, CHCl_3_); ^1^H NMR and ^13^C NMR (CDCl_3_, 700 MHz), Table 3 and Table 4; HRESIMS *m/z* 401.2291 [M + Na]^+^ (calculated for C_22_H_34_NaO_5_, 401.2298). 

(–)-12-Hydroxyspongian-16-one (**8**): colourless oil (0.08 mg); ${\left[\alpha \right]}_{D}^{21}$ –58 (*c* 0.01, CHCl_3_); ^1^H NMR and ^13^C NMR (CDCl_3_, 500 MHz), Table 3 and Table 4; HRESIMS *m/z* 343.2245 [M + Na]^+^ (calculated for C_20_H_32_NaO_3_, 343.2244). 

(–)-12-Hydroxy-20-oxyspongian-16-one-20-propionate (**9**): colourless oil (0.97 mg); ${\left[\alpha \right]}_{D}^{21}$ –7 (*c* 0.097, CHCl_3_); ^1^H NMR and ^13^C NMR (CDCl_3_, 500 MHz), Table 3 and Table 4; HRESIMS *m/z* 415.2458 [M + Na]^+^ (calculated for C_23_H_36_NaO_5_, 415.2455). 

(–)-12-Hydroxy-11,20-dioxyspongian-16-one-11,20-dipropionate (**10**): colourless oil (0.17 mg); ${\left[\alpha \right]}_{D}^{21}$ –35 (*c* 0.017, CHCl_3_); ^1^H NMR and ^13^C NMR (CDCl_3_, 700 MHz), Table 3 and Table 4; HRESIMS *m/z* 487.2668 [M + Na]^+^ (calculatedfor C_26_H_40_NaO_7_, 487.2666). 

(–)-11-Hydroxy-12,20-dioxyspongian-16-one-12,20-dipropionate (**11**): colourless oil (0.11 mg); ${\left[\alpha \right]}_{D}^{21}$ –64 (*c* 0.011, CHCl_3_); ^1^H NMR and ^13^C NMR (CDCl_3_, 700 MHz), Table 3 and Table 4; HRESIMS *m/z* 487.2667 [M + Na]^+^ (calculated for C_26_H_40_NaO_7_, 487.2666).

### 4.4. X-ray Crystallographic Structure Determination

Full details of X-ray crystallography methods and data are available in the Supplementary Materials. 

*Crystallographic data for **1******:*** C_27_H_42_O_7_ (*M* =478.60 g/mol): orthorhombic, space group P2_1_2_1_2_1_ (no. 19), *a* = 7.9081(2) Å, *b* = 11.0710(3) Å, *c* = 30.2192(8) Å, *V* = 2645.73(12) Å^3^, *Z* = 4, *T* = 100.01(10) K, μ(MoKα) = 0.085 mm^-1^, *Dcalc* = 1.202 g/cm^3^, 34647 reflections measured (4.562° ≤ 2Θ ≤ 56.56°), 6562 unique (*R*_int_ = 0.0495, R_sigma_ = 0.0347) which were used in all calculations. The final *R*_1_ was 0.0416 (I > 2σ(I)) and *wR*_2_ was 0.1191 (all data).

*Crystallographic data for****4****:* C_27_H_42_O_7_ (*M* =478.60 g/mol): orthorhombic, space group P2_1_2_1_2_1_ (no. 19), *a* = 7.9438(3) Å, *b* = 11.2958(8) Å, *c* = 28.897(2) Å, *V* = 2593.0(3) Å^3^, *Z* = 4, *T* = 99.99(10) K, μ(Mo Kα) = 0.087 mm^−1^, *Dcalc* = 1.226 g/cm^3^, 24810 reflections measured (4.578° ≤ 2Θ ≤ 50.246°), 4642 unique (*R*_int_ = 0.0727, R_sigma_ = 0.0429) which were used in all calculations. The final *R*_1_ was 0.0804 (I > 2σ(I)) and *wR*_2_ was 0.2256 (all data).

## Figures and Tables

**Table 1:** ^1^H NMR assignments for spongian-16-one analogues **1–5***^a^*.

Position	δ_H_, mult., *J* (Hz)				
	1 *^b^*	2 *^c^*	3 *^c,e^*	4 *^c^*	5 *^b^*
1 eq 1ax	2.22, br d (12.5) 0.89, m	2.34, m 0.78, m	1.75, m 0.86, m	2.22, m 0.89, m	1.74, m 0.86, m
2 eq 2ax	1.60, m 1.51, m	1.61, m 1.46, m	1.73, m 1.46, m	1.61, m 1.51, m	1.71, m 1.46, m
3eq 3ax	1.49, m 1.23, m	1.45, m 1.24, m	1.39, m 1.19, m	1.48, m 1.24, m	1.39, m 1.20, m
4	-	-	-	-	-
5	1.45, m	1.38, br s	1.16,	1.50, m	1.16, d (2.0)
6eq 6ax	1.81, m 1.63, m	4.17, br s -	4.18, br s -	1.81, m 1.63, m	4.19, br s -
7eq 7ax	4.85, t (2.8) -	4.87, d (3.2) -	4.84, d (2.6) -	4.86, t (2.6) -	4.84, d (3.1) -
8	-	-	-	-	-
9	1.49, m	1.14, m	1.05, m	1.44, m	1.05, dd(2.2, 12.5)
10	-	-	-	-	-
11eq 11ax	1.92, m 1.89, m	1.80, m 1.58, m	1.58, m 1.47, m	1.88, m 1.88, m	1.57, m 1.45, m
12eq 12ax	2.28, dt(13.9, 5.8) 2.02, m	2.31, m 1.55, m	2.32, m 1.60, m	2.32, dt(14.1, 5.6) 1.99, m	2.32, m 1.60, m
13	-	2.57, t (7.9)	2.57, t (7.8)	-	2.60, t (8.2)
14	2.92, dd (1.5, 6.5)	2.49, m	2.44, dd (5.6, 7.8)	2.91, dd (1.2, 6.3)	2.43, dd (5.6, 8.2)
15eq 15ax	4.22, dd (6.5, 9.9) 4.20, dd(1.5, 9.9)	4.25, d (10.2) 3.94, dd (5.5, 10.2)	4.27, d (10.1) 3.95, dd (5.6, 10.1)	4.25, dd (6.3, 9.9) 4.20, dd (1.2, 9.9)	4.26, d (10.2) 3.98, dd (5.6, 10.2)
16	-	-	-	-	-
17	1.10, s	1.27, s	1.22, s	1.09, s	1.21, s
18eq	0.81, s	0.90, s	0.90, s	0.81, s	0.90, s
19ax	0.81, s	1.18, s	1.19, s	0.80, s	1.19, s
20a 20b	4.05, d (11.8) 3.92, d (11.8)	4.79, m 4.73, m	1.19, s	4.04, d (11.8) 3.91, d (11.8)	1.19, s
6-OH	-	*^d^*	*^d^*	-	*^d^*
7-CO_2_CH_3_	-	-	-	-	2.09, s
7-CO_2_*CH_2_*CH(CH_3_)_2_	-	-	-	2.22, m 2.22, m	-
7-CO_2_CH_2_*CH*(CH_3_)_2_	-	-	-	2.11, m	-
7-CO_2_CH_2_CH(*CH_3_*)_2_	-	-	-	0.95, d (6.6) 0.96, d (6.6)	-
7-CO_2_*CH*CH_3_CH_2_CH_3_	2.40, m (7.1)	2.41, q (6.9)	2.45, m	-	-
7-CO_2_CH*CH_3_*CH_2_CH_3_	1.15, d (6.9)	1.15, d (6.9)	1.15, d (7.2)	-	-
7-CO_2_CHCH_3_*CH_2_*CH_3_	1.68, dt (13.6, 7.4) 1.48, m	1.68, dt (13.6, 7.4) 1.50, m	1.68, m 1.49, m	-	-
7-CO_2_CHCH_3_CH_2_*CH_3_*	0.89, t (7.4)	0.91, t (7.4)	0.90, t (7.2)	-	-
13- CO_2_*CH_3_*	2.04, s	-	-	2.04, s	-
20-CO_2_*CH_3_*	-	2.03, s	-	-	-
20-OH	*^d^*	-	-	*^d^*	-

*^a^* Chemical shifts (ppm) referenced to CHCl_3_ (δ_H_ 7.26, δ_C_ 77.16). *^b^* At 500 MHz. *^c^* At 700 MHz. *^d^* Not observed. *^e^* Data acquired using a Shigemi NMR tube.

**Table 2:** ^13^C NMR assignments for spongian-16-one analogues **1–5***^a^*.

Position	δ_C_, mult.				
	1 *^b^*	2 *^c^*	3 *^c,d^*	4 *^c^*	5 *^b^*
1	34.6, CH_2_	36.6, CH_2_	42.4, CH_2_	34.9, CH_2_	42.4, CH_2_
2	18.7, CH_2_	18.6, CH_2_	18.7, CH_2_	18.8, CH_2_	18.7, CH_2_
3	41.7, CH_2_	43.4, CH_2_	44.1, CH_2_	41.7, CH_2_	43.9, CH_2_
4	32.2, C	33.5, C	33.7, C	32.3, C	33.2, C
5	48.1, CH	51.8, CH	51.3, CH	47.9, CH	51.2, CH
6	23.1, CH_2_	69.6, CH	70.6, CH	23.1, CH_2_	70.5, CH
7	73.4, CH	75.2, CH	75.7, CH	73.6, CH	76.1, CH
8	39.5, C	37.9, C	37.7, C	39.3, C	37.7, C
9	50.1, CH	52.4, CH	51.9, CH	50.3, CH	51.8, CH
10	39.5, C	40.8, C	36.8, C	41.5, C	36.7, C
11	18.7, CH_2_	19.1, CH_2_	17.4, CH_2_	18.8, CH_2_	17.4, CH_2_
12	27.3, CH_2_	22.5, CH_2_	21.8, CH_2_	27.5, CH_2_	22.0, CH_2_
13	81.1, C	36.9, CH	37.0, CH	81.0, CH	37.0, CH
14	45.9, CH	41.9, CH	41.6, CH	45.9, CH	41.9, CH
15	66.9, CH_2_	67.1, CH_2_	67.2, CH_2_	66.9, CH_2_	67.4, CH_2_
16	174.0, C	178.5, C	178.9, C	173.7, C	178.9, C
17	16.1, CH_3_	15.1, CH_3_	15.0, CH_3_	15.9, CH_3_	14.9, CH_3_
18eq	33.4, CH_3_	33.8, CH_3_	33.2, CH_3_	33.6, CH_3_	33.4, CH_3_
19ax	21.8, CH_3_	24.6, CH_3_	24.3, CH_3_	21.9, CH_3_	24.5, CH_3_
20	62.1, CH_2_	64.1, CH_2_	17.6, CH_3_	62.1, CH_2_	18.0, CH_3_
	-			-	
7-*C*O_2_CH_3_	-	-	-	-	169.7, C
7-CO_2_*CH_3_*	-	-	-		21.4, CH_3_
7-*C*O_2_CH_2_CH(CH_3_)_2_	-	-	-	171.9, C	-
7-CO_2_*CH_2_*CH(CH_3_)_2_	-	-	-	44.0, CH_2_	-
7-CO_2_CH_2_*CH*(CH_3_)_2_	-	-	-	25.7, CH	
7-CO_2_CH_2_CH(*CH_3_*)_2_	-	-	-	22.5, CH_3_ 22.5, CH_3_	-
7-*C*O_2_CHCH_3_CH_2_CH_3_	175.4, C	174.9, C	175.3, C	-	-
7-CO_2_*CH*CH_3_CH_2_CH_3_	41.8, CH	41.2, CH	41.6, CH	-	-
7-CO_2_CH*CH_3_*CH_2_CH_3_	16.9, CH_3_	16.9, CH_3_	16.9, CH_3_	-	-
7-CO_2_CHCH_3_*CH_2_*CH_3_	26.8, CH_2_	26.8, CH_2_	26.9, CH_2_	-	-
7-CO_2_CHCH_3_CH_2_*CH_3_*	11.8, CH_3_	11.8, CH_3_	11.7, CH_3_	-	-
13-*C*O_2_CH_3_	169.7, C	-	-	169.6, C	-
13-CO_2_*CH_3_*	21.5, CH_3_	-	-	21.6, CH_3_	-
20-*C*O_2_CH_3_	-	170.6, C	-	-	-
20-CO_2_*CH_3_*	-	21.2, CH_3_	-	-	-

*^a^* Chemical shifts (ppm) referenced to CHCl_3_ (δ_H_ 7.26, δ_C_ 77.16). *^b^* At 500 MHz. *^c^* At 700 MHz. *^d^* Data acquired using a Shigemi NMR tube.

**Table 3:** ^1^H NMR assignments for spongian-16-one analogues **6–11***^a^*.

Position	δ_H_, mult., *J* (Hz)					
	6 *^c^*	7 *^c^*	8 *^b^*	9 *^b^*	10 *^c^*	11 *^c^*
1 eq 1ax	2.03, m 0.62, m	2.12, m 0.80, m	1.68, br d (12.8) 0.83, m	2.07, br d (13.2) 0.79, td (13.2, 2.3)	2.03, br d (13.4) 0.75, m	2.02, br d (13.6) 1.15, m
2 eq 2ax	1.54, m 1.43, m	1.56, m 1.45, m	1.61, m 1.42, m	1.57, m 1.46, m	1.61, m 1.47, m	1.62, m 1.50, m
3eq 3ax	1.45, m 1.16, m	1.45, m 1.17, m	1.38, m 1.15, td (13.2, 3.7)	1.45, m 1.19, m	1.45, m 1.16, m	1.45, br d (12.9) 1.18, m
4	-	-	-	-	-	-
5	1.03, m	1.01, dd (12.4, 2.1)	0.89, m	1.08, dd (12.3, 1.7)	1.05, dd (12.7, 2.4)	1.11, m
6eq 6ax	1.57, m 1.40, m	1.56, m 1.38, m	1.55, m 1.35, m	1.58, m 1.39, m	1.66, m 1.45, m	1.63, m 1.49, m
7eq 7ax	1.92, m 1.16, m	1.88, m 1.16, m	1.82, dt (12.8, 3.3) 1.09, dt (12.8, 3.5)	1.91, dt (12.8, 3.3) 1.17, m	1.76, dt (12.6, 3.1) 1.06, m	1.76, dt (12.6, 3.2) 1.06, td (12.6, 3.7)
8	-	-	-	-	-	-
9	1.33, m	1.04, m	1.32, dd (9.1, 6.3)	1.51, m	1.35, d (2.9)	1.34, d (3.0)
10	-	-	-	-	-	-
11eq 11ax	2.00, m 1.80, dd (13.2, 3.4)	1.88, m 1.49, m	1.63, m 1.63, m	1.87, m 1.85, m	5.95, t (3.4) -	4.46, t (3.0) -
12eq 12ax	5.44, br s -	2.63, m 1.62, m	4.52, br s -	4.49, br s -	4.36, m -	5.54, dd (9.2, 3.0) -
13	2.67, dt (8.0, 1.5)	-	2.66, d (8.0)	2.65, d (7.9)	2.84, dd (10.9, 9.4)	3.00, dd (10.6, 9.2)
14	2.29, dd (8.0, 5.2)	1.94, dd (7.8, 5.6)	2.33, dd (8.0, 5.4)	2.37, dd (7.9, 5.4)	2.44, m	2.44, m
15eq 15ax	4.26, d (9.9) 4.12, m	4.42, dd (9.4, 5.6) 4.13, dd (9.4, 7.8)	4.23, d (9.7) 4.11, dd (9.7, 5.4)	4.26, d (9.9) 4.13, dd (9.9, 5.4)	4.33, m 4.33, m	4.28, m 4.28, m
16	-	-	-	-	-	-
17	0.90, s	0.88, s	0.82, s	0.89, s	0.95, s	0.94, s
18eq	0.89, s	0.89, s	0.86, s	0.90, s	0.87, s	0.87, s
19ax	0.83, s	0.83, s	0.81, s	0.85, s	0.81, s	0.82, s
20a 20b	4.56, d (12.4) 4.13, m	4.55, d (13.1) 4.14, d (13.1)	0.82, s	4.59, d (12.1) 4.17, d (12.1)	4.74, d (12.0) 3.96, dd (12.0, 1.9)	4.61, d (12.2) 4.02, dd (12.2, 1.8)
11-OH	-	-	-	-	-	2.08, br s
11-CO_2_*CH_2_*CH_3_	-	-	-	-	2.34, m 2.34, m	-
11-CO_2_CH_2_*CH_3_*	-	-	-	-	1.15, t (7.7)	-
12-OH	-	-	*^d^*	*^d^*	2.79, br s	-
12-OCO*CH_3_*	-	-	-	-	-	-
12-CO_2_*CH_2_*CH_3_	2.33, q (7.6) 2.33, q (7.6)	-	-	-	-	2.46, m 2.40, m
12-CO_2_CH_2_*CH_3_*	1.16, t (7.7)	-	-	-	-	1.18, t (7.6)
13-OH	-	*^d^*	-	-	-	-
20-OCO*CH_3_*	2.03, s	2.02, s	-	-	-	-
20-CO_2_*CH_2_*CH_3_	-	-	-	2.31, q (7.7) 2.31, q (7.7)	2.46, m 2.46, m	2.50, m 2.45, m
20-CO_2_CH_2_*CH_3_*	-	-	-	1.13, t (7.7)	1.18, t (7.7)	1.12, t (7.5)

*^a^* Chemical shifts (ppm) referenced to CHCl_3_ (δ_H_ 7.26, δ_C_ 77.16). *^b^* At 500 MHz. *^c^* At 700 MHz. *^d^* Not observed.

**Table 4:** ^13^C NMR assignments for spongian-16-one analogues **6–11***^a^*.

Position	δ_C_, mult.					
	6 *^c^*	7 *^c^*	8 *^b^*	9 *^b^*	10 *^c^*	11 *^c^*
1	35.1, CH_2_	35.4, CH_2_	39.9, CH_2_	35.1, CH_2_	33.8, CH_2_	33.8, CH_2_
2	18.3, CH_2_	18.5, CH_2_	18.5, CH_2_	18.4, CH_2_	18.2, CH_2_	18.2, CH_2_
3	41.5, CH_2_	41.6, CH_2_	42.1, CH_2_	41.7, CH_2_	41.3, CH_2_	41.5, CH_2_
4	32.8, C	33.0, C	33.1, C	33.1, C	33.0, C	32.9, C
5	57.1, CH	57.0, CH	56.8, CH	56.9, CH	58.0, CH	58.2, CH
6	17.8, CH_2_	17.9, CH_2_	18.1, CH_2_	17.9, CH_2_	17.6, CH_2_	18.1, CH_2_
7	42.2, CH_2_	42.8, CH_2_	42.0, CH_2_	42.2, CH_2_	42.1, CH_2_	41.9, CH_2_
8	35.6, C	36.3, C	35.5, C	36.0, C	35.1, C	33.2, C
9	50.0, CH	56.7, CH	48.8, CH	49.0, CH	62.6, CH	64.2, CH
10	39.8, C	40.4, C	36.1, C	40.2, C	41.2, C	40.7, C
11	25.6, CH_2_	19.4, CH_2_	27.1, CH_2_	29.2, CH_2_	70.4, CH	67.7, CH
12	67.9, CH	28.1, CH_2_	65.1, CH	64.9, CH	66.4, CH	69.5, CH
13	43.1, CH	83.7, C	45.5, CH	45.6, CH	41.6, CH	38.7, CH
14	49.1, CH	54.5, CH	48.3, CH	48.6, CH	47.6, CH	47.7, CH
15	67.7, CH_2_	67.1, CH_2_	67.9, CH_2_	68.0, CH_2_	67.7, CH_2_	66.9, CH_2_
16	174.9, C	173.6, C	176.3, C	176.4, C	180.3, C	178.2, C
17	15.3, CH_3_	15.5, CH_3_	15.4, CH_3_	15.2, CH_3_	17.7, CH_3_	17.6, CH_3_
18eq	33.9, CH_3_	33.9, CH_3_	33.5, CH_3_	33.9, CH_3_	33.6, CH_3_	33.6, CH_3_
19ax	22.1, CH_3_	22.0, CH_3_	21.6, CH_3_	21.9, CH_3_	21.9, CH_3_	21.7, CH_3_
20	64.3, CH_2_	64.3, CH_2_	16.5, CH_3_	64.5, CH_2_	64.4, CH_2_	64.7, CH_2_
11-*C*O_2_CH_2_CH_3_	-	-	-	-	173.6, C	-
11-CO_2_*CH_2_*CH_3_	-	-	-	-	27.9, CH_2_	-
11-CO_2_CH_2_*CH_3_*	-	-	-	-	9.2, CH_3_	-
12-*C*O_2_CH_2_CH_3_	173.0, C	-	-	174.5, C	-	172.6, C
12-CO_2_*CH_2_*CH_3_	28.0, CH_2_	-	-	27.9, CH_2_	-	27.7, CH_2_
12-CO_2_CH_2_*CH_3_*	9.4, CH_3_	-	-	9.3, CH_3_	-	9.1, CH_3_
20-*C*O_2_CH_3_	170.8, C	170.8, C	-	-	-	-
20-CO_2_*CH_3_*	21.2, CH_3_	21.2, CH_3_	-	-	-	-
20-*C*O_2_CH_2_CH_3_	-	-	-	-	174.2, C	175.5, C
20-CO_2_*CH_2_*CH_3_	-	-	-	-	27.6, CH_2_	27.4, CH_2_
20-CO_2_CH_2_*CH_3_*	-	-	-	-	9.0, CH_3_	9.4, CH_3_

*^a^* Chemical shifts (ppm) referenced to CHCl_3_ (δ_H_ 7.26, δ_C_ 77.16). *^b^* At 500 MHz. *^c^* At 700 MHz.

## Data Availability

Raw NMR data files are available from the authors on request. All other data is contained within this manuscript.

## Supplementary Materials

The following are available online at https://www.mdpi.com/article/10.3390/md19120680/s1. Figure S1: An image of *G. aureopurpureus* and *Giniobranchus* sp. 1; Figure S2: X-ray crystallography image of diterpene **4**; Figures S3–S56: NMR and 2D NMR spectra of diterpenes **1**–**11** (^1^H, COSY, HSQC, HMBC, and NOESY). Tables S1 and S2 and Figures S57–S62: Anatomical distribution of metabolites.
