# Supplementary material for: Expanding the Repertoire of Spongian-16-One Derivatives in Australian Nudibranchs of the Genus Goniobranchus and Evaluation of Their Anatomical Distribution
Source: Mar Drugs. 2021 Nov 29;19(12):680. doi: 10.3390/md19120680 (PMC8706817; doi:10.3390/md19120680)

Expanding the repertoire of spongian-16-one derivatives by evaluation of their anatomical distribution in Australian nudibranchs of the genus *Goniobranchus*.

Louise C. Forster[1], Jack K. Clegg[1], Karen L. Cheney[2], and Mary J. Garson[1,*]

[1] School of Chemistry and Molecular Biosciences, The University of Queensland, St. Lucia, QLD 4072, Australia; l.forster1@uq.edu.au; j.clegg@uq.edu.au; m.garson@uq.edu.au.

[2] School of Biological Sciences, The University of Queensland, St. Lucia, QLD 4072, Australia; k.cheney@uq.edu.au.

Contents

Anatomical distribution....4
Anatomical distribution of metabolites in *G. aureopurpureus*....4
Table S1 Distribution of diterpenes in *G. aureopurpureus* organs....4
Anatomical distribution of metabolites in *Goniobranchus* sp. 1....5
Table S2 Distribution of diterpenes in *Goniobranchus* sp. 1 organs....5
X-Ray crystallography....5
X-ray crystallographic structure determination....5
Figure S2. Oak Ridge Thermal Ellipsoid Plot (ORTEP) representation of the crystal structure of (-)-13-acetoxy-20-hydroxy-7α-oxyspongian-16-one-7α-(3-methyl)-butanoate 4 shown with only one component of each region of disorder....6
Assorted spectra....7
Figure S3. ^{1}H NMR spectrum of 1 (700 MHz, $CDCl_3$)....7
Figure S4. HSQC spectrum of 1 (700 MHz, $CDCl_3$)....7
Figure S5. HMBC spectrum of 1 (700 MHz, $CDCl_3$)....8
Figure S6. COSY spectrum of 1 (700 MHz, $CDCl_3$)....8
Figure S7. NOESY spectrum of 1 (700 MHz, $CDCl_3$)....9
Figure S8. ^{1}H NMR spectrum of 2 (700 MHz, $CDCl_3$)....9
Figure S9. HSQC spectrum of 2 (700 MHz, $CDCl_3$)....10
Figure S10. HMBC spectrum of 2 (700 MHz, $CDCl_3$)....10
Figure S11. COSY spectrum of 2 (700 MHz, $CDCl_3$)....11
Figure S12. NOESY spectrum of 2 (700 MHz, $CDCl_3$)....11
Figure S13. ^{1}H NMR spectrum of 3 (700 MHz, $CDCl_3$), collected in a Shigemi NMR tube....12
Figure S14. HSQC spectrum of 3 (700 MHz, $CDCl_3$), collected in a Shigemi NMR tube....12
Figure S15. HMBC spectrum of 3 (700 MHz, $CDCl_3$), collected in a Shigemi NMR tube....13
Figure S16. COSY spectrum of 3 (700 MHz, $CDCl_3$), collected in a Shigemi NMR tube....13
Figure S17. ^{1}H NMR spectrum of 4 (700 MHz, $CDCl_3$)....14
Figure S18. HSQC spectrum of 4 (700 MHz, $CDCl_3$)....14
Figure S19. HMBC spectrum of 4 (700 MHz, $CDCl_3$)....15
Figure S20. COSY spectrum of 4 (700 MHz, $CDCl_3$)....15
Figure S21. NOESY spectrum of 4 (700 MHz, $CDCl_3$)....16
Figure S22. ^{1}H NMR spectrum of 5 (700 MHz, $CDCl_3$)....16
Figure S23. HSQC spectrum of 5 (700 MHz, $CDCl_3$)....17
Figure S24. HMBC spectrum of 5 (700 MHz, $CDCl_3$)....17
Figure S25. COSY spectrum of 5 (700 MHz, $CDCl_3$)....18
Figure S26. NOESY spectrum of 5 (700 MHz, $CDCl_3$)....18
Figure S27. ^{1}H NMR spectrum of 6 (700 MHz, $CDCl_3$)....19
Figure S28. HSQC spectrum of 6 (700 MHz, $CDCl_3$)....19
Figure S29. HMBC spectrum of 6 (700 MHz, $CDCl_3$)....20

Figure S30. COSY spectrum of 6 (700 MHz, $CDCl_3$). 20
Figure S31. NOESY spectrum of 6 (700 MHz, $CDCl_3$). 21
Figure S32. ^{1}H NMR spectrum of 7 (700 MHz, $CDCl_3$). 21
Figure S33. HSQC spectrum of 7 (700 MHz, $CDCl_3$). 22
Figure S34. HMBC spectrum of 7 (700 MHz, $CDCl_3$). 22
Figure S35. COSY spectrum of 7 (700 MHz, $CDCl_3$). 23
Figure S36. NOSEY spectrum of 7 (700 MHz, $CDCl_3$). 23
Figure S37. ^{1}H NMR spectrum of 8 (700 MHz, $CDCl_3$). 24
Figure S38. HSQC spectrum of 8 (500 MHz, $CDCl_3$). 24
Figure S39. HMBC spectrum of 8 (700 MHz, $CDCl_3$). 25
Figure S40. COSY spectrum of 8 (500 MHz, $CDCl_3$). 25
Figure S41. NOESY spectrum of 8 (700 MHz, $CDCl_3$). 26
Figure S42. ^{1}H NMR spectrum of 9 (500 MHz, $CDCl_3$). 26
Figure S43. HSQC spectrum of 9 (500 MHz, $CDCl_3$). 27
Figure S44. HMBC spectrum of 9 (500 MHz, $CDCl_3$). 27
Figure S45. COSY spectrum of 9 (500 MHz, $CDCl_3$). 28
Figure S46. NOESY spectrum of 9 (500 MHz, $CDCl_3$). 28
Figure S47. ^{1}H NMR spectrum of 10 (700 MHz, $CDCl_3$). 29
Figure S48. HSQC spectrum of 10 (700 MHz, $CDCl_3$). 29
Figure S49. HMBC spectrum of 10 (700 MHz, $CDCl_3$). 30
Figure S50. COSY spectrum of 10 (700 MHz, $CDCl_3$). 30
Figure S51. NOESY spectrum of 10 (700 MHz, $CDCl_3$). 31
Figure S52. ^{1}H NMR spectrum of 11 (700 MHz, $CDCl_3$). 31
Figure S53. HSQC spectrum of 11 (700 MHz, $CDCl_3$). 32
Figure S54. HMBC spectrum of 11 (700 MHz, $CDCl_3$). 32
Figure S55. COSY spectrum of 11 (700 MHz, $CDCl_3$). 33
Figure S56. NOESY spectrum of 11 (700 MHz, $CDCl_3$). 33
Figure S57. Overlay of ^{1}H NMR spectra for the mantle of six specimens of *G. aureopurpureus* (500 MHz, $CDCl_3$). 34
Figure S58. Overlay of ^{1}H NMR spectra for the viscera of six specimens of *G. aureopurpureus* (500 MHz, $CDCl_3$). 35
Figure S59. Overlay of ^{1}H NMR spectra for the mantle and viscera of *G. aureopurpureus* (500 MHz, $CDCl_3$). 36
Figure S60. Overlay of ^{1}H NMR spectra for the mantle of three specimens of *Goniobranchus sp.1* (500 MHz, $CDCl_3$). 37
Figure S61. Overlay of ^{1}H NMR spectra for the viscera of three specimens of *Goniobranchus sp.1* (500 MHz, $CDCl_3$). 38
Figure S62. Overlay of ^{1}H NMR spectra for the mantle and viscera of *Goniobranchus sp.1* (500 MHz, $CDCl_3$). 39

Figure S1. Image of the specimen of *Goniobranchus aureopurpureus* and *Goniobranchus* sp. 1.

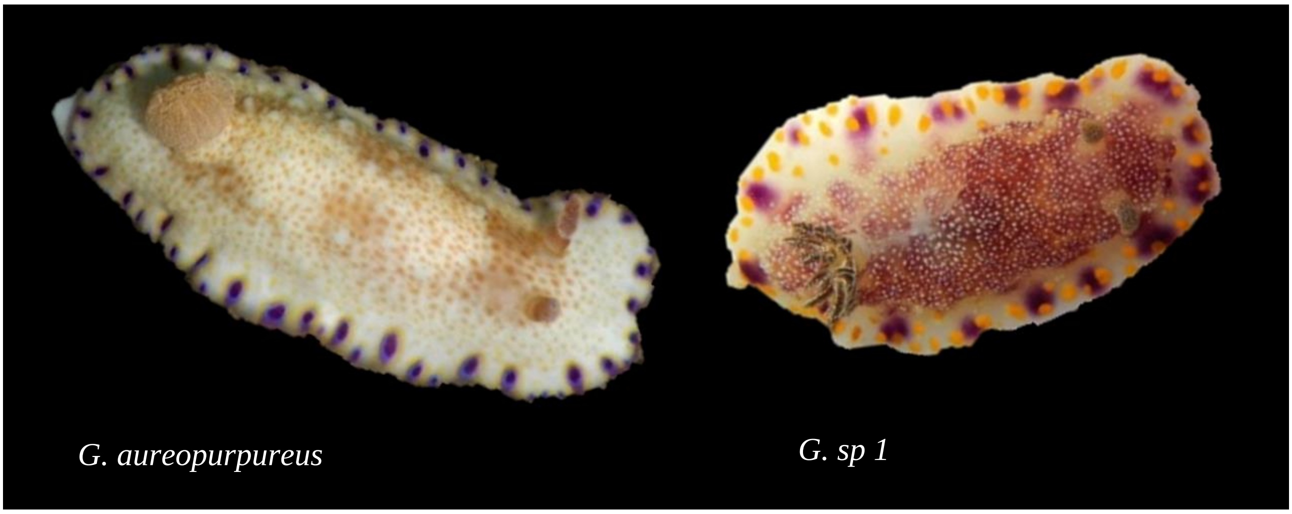

Anatomical distribution

Anatomical distribution of metabolites in *G. aureopurpureus*

The specimens of *G. aureopurpureus* were dissected into their mantle and viscera. The body parts were extracted separately, and the extracts compared by ^{1}H NMR spectroscopy (**Table S1**). The new metabolites (**1-5**) were only isolated from the mantle tissue.

Table S1 Distribution of diterpenes in *G. aureopurpureus* organs.

Compound	Present in
15-desacetoxy-12-acetoxydendrillolide A	Mantle
7α-acetoxy-6β-hydroxyspongian-16-one (**5**)	Mantle
6β-hydroxy-7α-oxyspongian-16-one-7α-(2-methyl)-butanoate (**3**)	Mantle
20-acetoxy-6β-hydroxy-7α-oxyspongian-16-one-7α-(2-methyl)-butanoate (**2**)	Mantle
13-acetoxy-20-hydroxy-7α-oxyspongian-16-one-7α-(2-methyl)-butanoate (**1**)	Mantle
13-acetoxy-20-hydroxy-7α-oxyspongian-16-one-7α-(3-methyl)-butanoate (**4**)	Mantle
shahamin C	Mantle
luffarin-X	Viscera
polyrhaphin A	Viscera
15,16-diacetoxyshahamin B	Viscera
12-desacetoxypolyrhaphin A	Viscera
spongian-16-one	Both tissues
7α-acetoxyspongian-16-one	Both tissues
macfarlandin E	Both tissues
aplyviolene	Both tissues
polyrhaphin B	Both tissues
secoshahamin	Both tissues

Anatomical distribution of metabolites in *Goniobranchus* sp. 1

The specimens of *Goniobranchus* sp. 1 were dissected into the mantle and viscera. The anatomical distribution was explored through the comparison of the mantle and viscera chemical profiles (**Table S2**). Like *G. aureopurpureus* only the mantle was found to have the newly elucidated metabolites (**6-11**).

Table S2 Distribution of diterpenes in *Goniobranchus* sp. 1 organs.

Compound	Present in
20-acetoxy,12α-oxyspongian-16-one-12α-propionate (**6**)	Mantle
20-acetoxy-13-hydroxy-spongian-16-one (**7**)	Mantle
12-hydroxyspongian-16-one (**8**)	Mantle
12-hydroxy-20-oxyspongian-16-one-20-propionate (**9**)	Mantle
12-hydroxy-11,20-dioxyspongian-16-one-11,20-dipropionate (**10**)	Mantle
11-hydroxy-12,20-dioxyspongian-16-one-12,20-dipropionate (**11**)	Mantle
20-oxyspongian-16-one-propoionate	Mantle
12α,20-dioxyspongian-16-one-dipropionate	Mantle
12α-acetoxy,20-oxyspongian-16-one-20-propionate	Mantle
spongian-16-one	Viscera
7α-acetoxyspongian-16-one	Viscera
isoagatholactone	Both tissues
12α-acetoxyspongian-16-one	Both tissues
20-acetoxyspongian-16-one	Both tissues
12α,20-diacetoxyspongian-16-one	Both tissues

X-Ray crystallography

X-ray crystallographic structure determination

Data were collected using an Oxford Rigaku Synergy-S employing confocal mirror monochromated Mo-K_α radiation generated from a microfocus source (0.71073 Å) with ω and ψ scans at 100(2) K. Data integration and reduction were undertaken with CrysAlisPro [1]. Subsequent computations were carried out using Olex2 [2]. Structures were solved with ShelXT [3] and refined and extended with ShelXL [4]. While the Flack parameters had an error associated with them, the relative configurations of each structure are unambiguous. The refinement of **1** was unremarkable, while the refinement of **4** was affected by disorder present in the structure for the 3-methylbutanoate side chain. The model employed indicated that the crystal was actually a 3:1 mixture of **4** and **1**. The ester side-chains were modelled isotropically. Crystallographic data are summarized below and the CIF has been deposited at the Cambridge Crystallographic Data Centre with CCDC 2117159-2117160. It is available free of charge from the Cambridge Crystallographic Data Centre, 12 16 Union Road, Cambridge CB2 1 EZ, UK; fax: (+44) 1223-336-033; or e-mail: deposit@ccdc.cam.ac.uk.

References:

1. Rigaku Oxford Diffraciton *CrysAlisPro* Rigaku Oxford Diffraction Ltd: Yarton, Oxfordshire, UK, **2009-2021**

2. Dolomanov, O. V.; Bourhis, L. J.; Gildea, R. J.; Howard, J. A. K.; Puschmann, H., *OLEX2*: a complete structure solution, refinement and analysis program, *J. Appl. Cryst.* **2009**, *42*, 339

3. Sheldrick, G. M., SHELXT - Integrated space-group and crystal-structure determination, *Acta. Cryst.* **2015**, *A71*, 3-8.

4. Sheldrick, G. M., Crystal structure refinement with SHELXL, *Acta. Cryst.* **2015**, *C71*, 3-8.

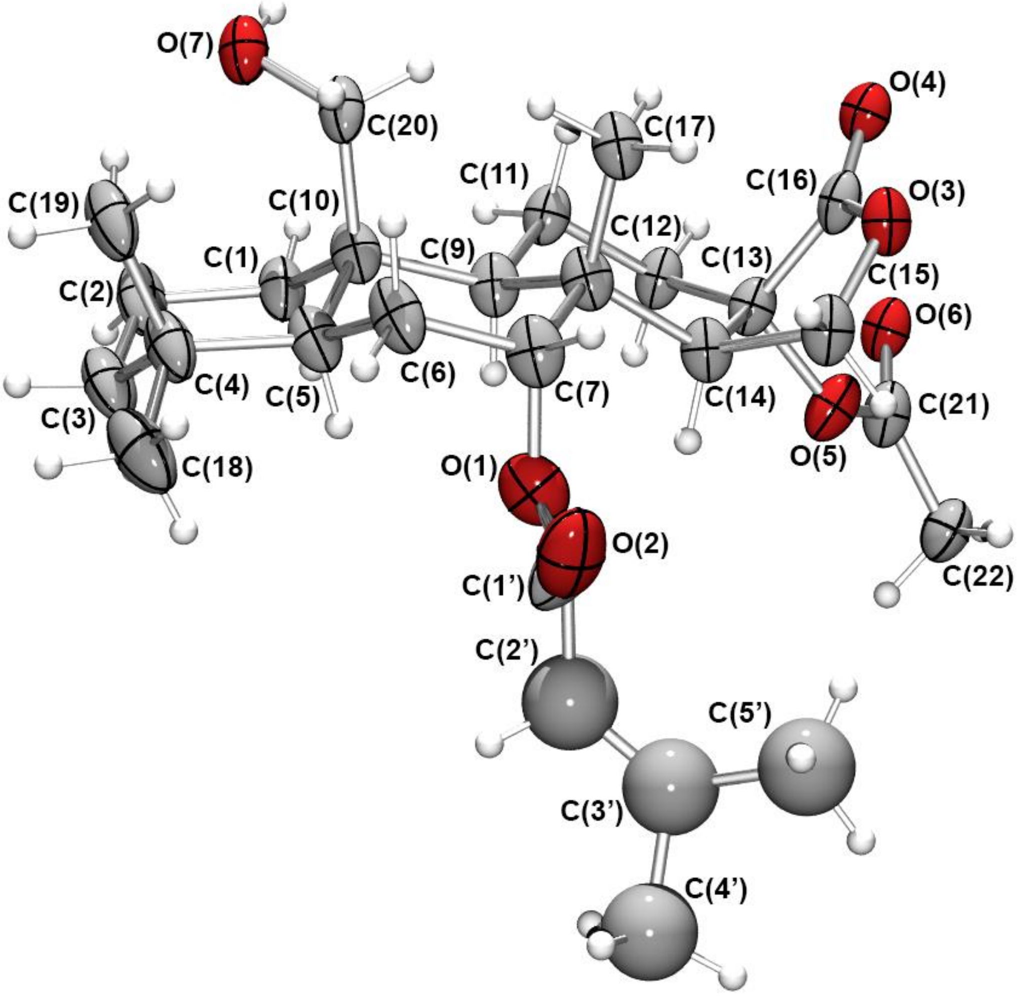

Figure S2. Oak Ridge Thermal Ellipsoid Plot (ORTEP) representation of the crystal structure of (-)-13-acetoxy-20-hydroxy-7α-oxyspongian-16-one-7α-(3-methyl)-butanoate **4** shown with only one component of each region of disorder.

Assorted spectra

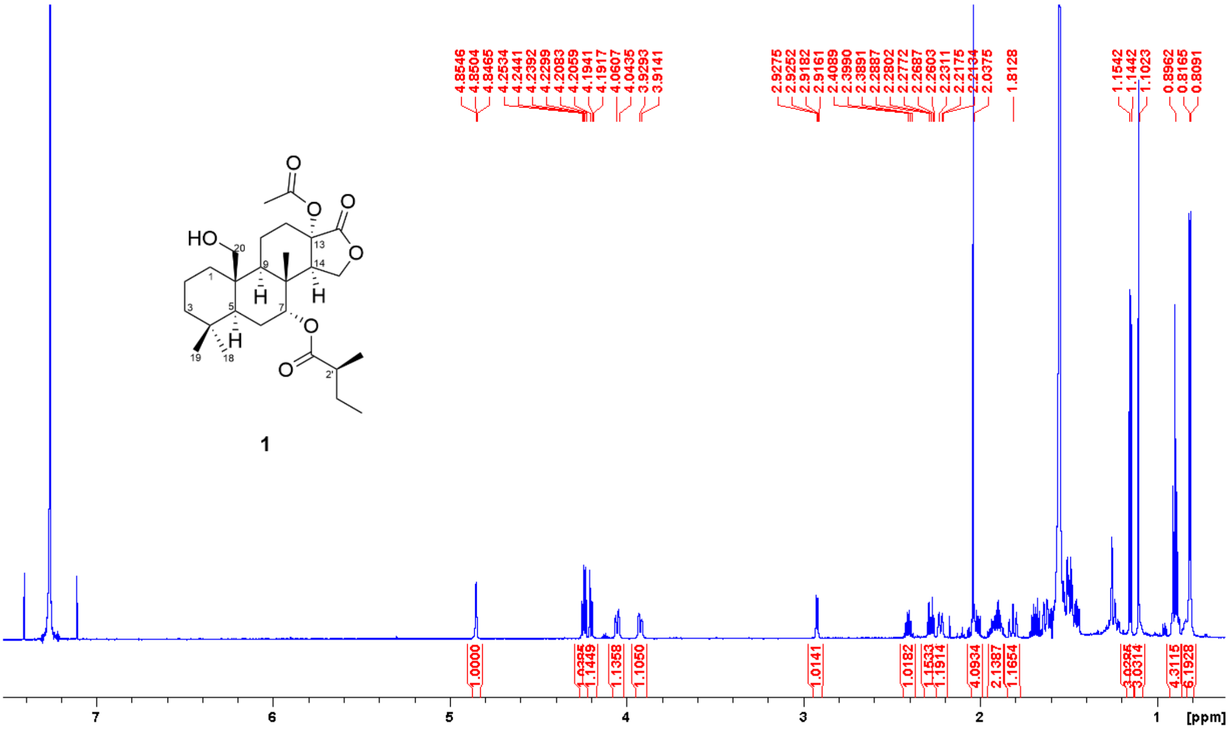

Figure S3. ^{1}H NMR spectrum of **1** (700 MHz, $CDCl_3$).

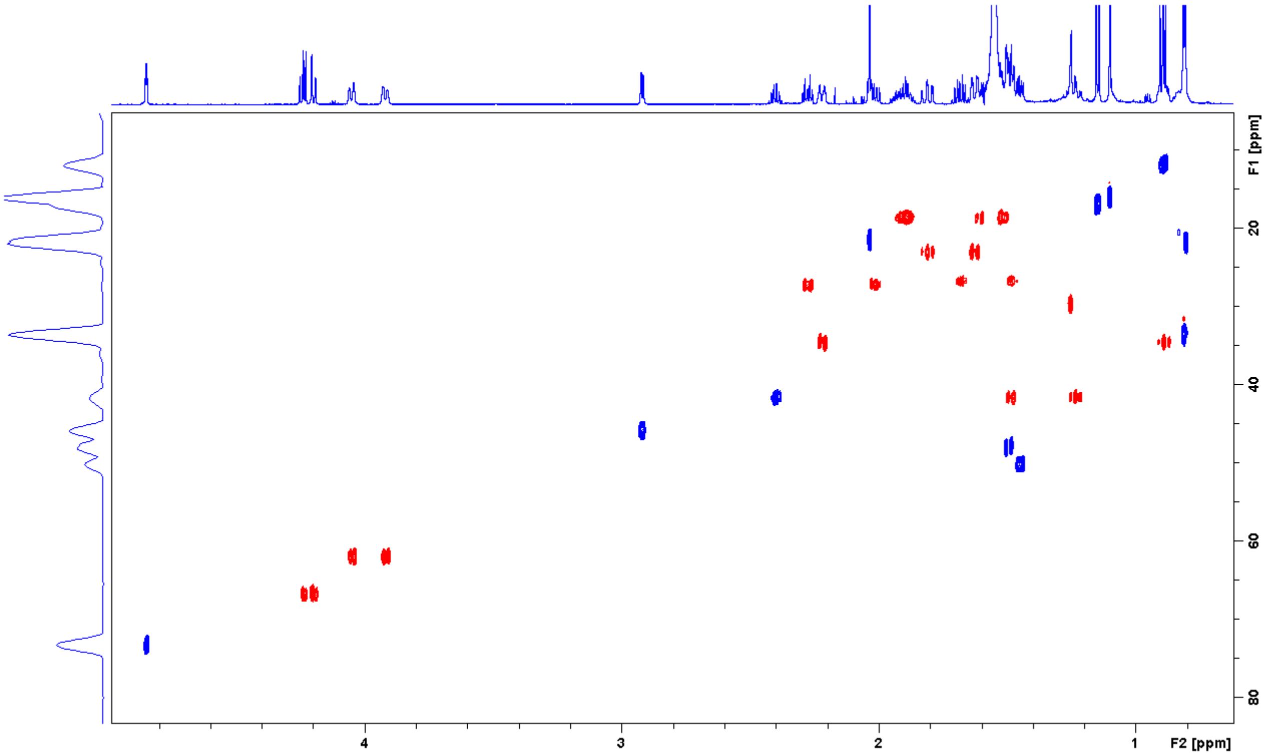

Figure S4. HSQC spectrum of **1** (700 MHz, $CDCl_3$).

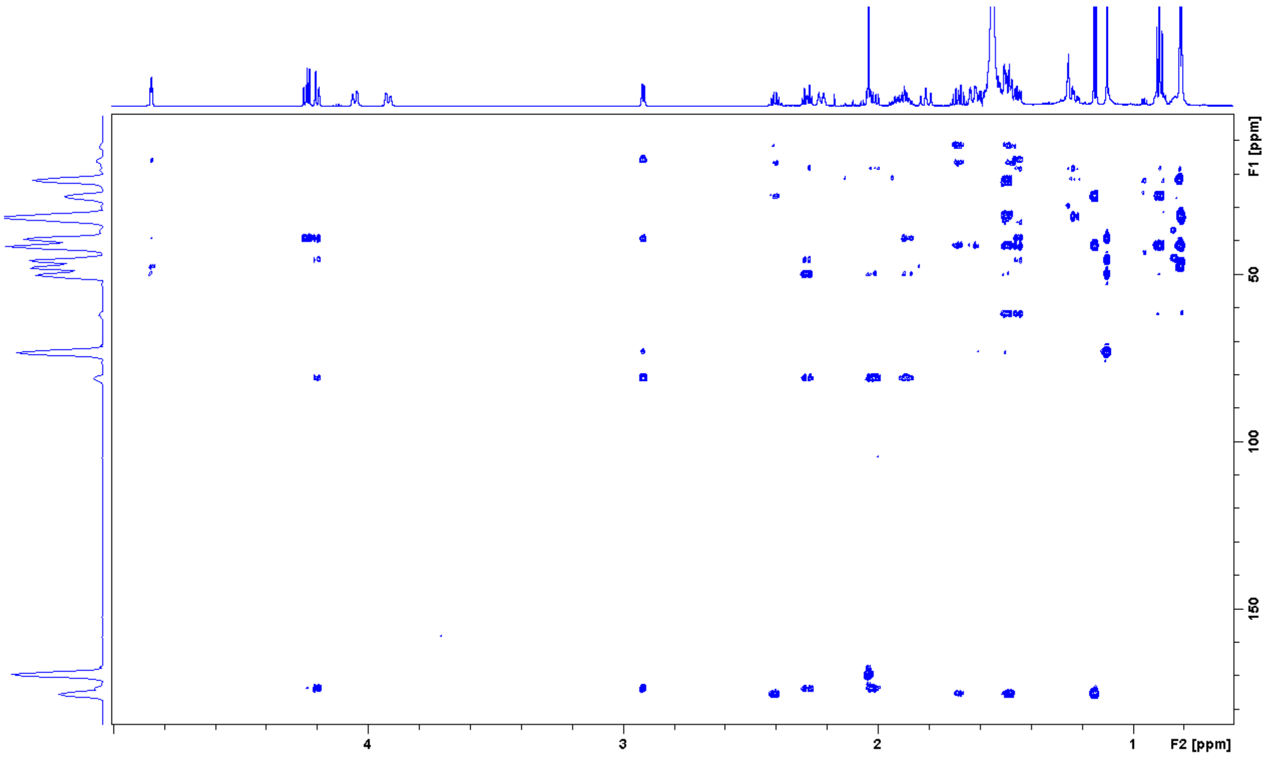

Figure S5. HMBC spectrum of **1** (700 MHz, $CDCl_3$).

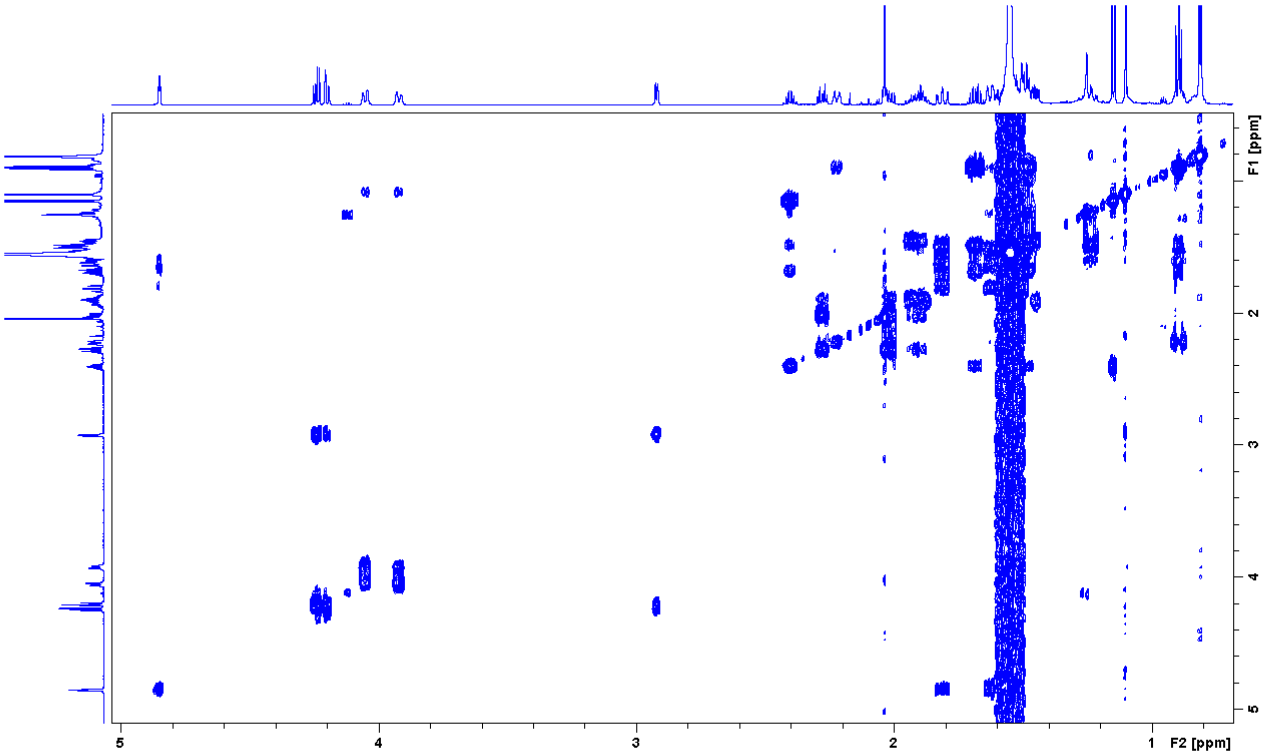

Figure S6. COSY spectrum of **1** (700 MHz, $CDCl_3$).

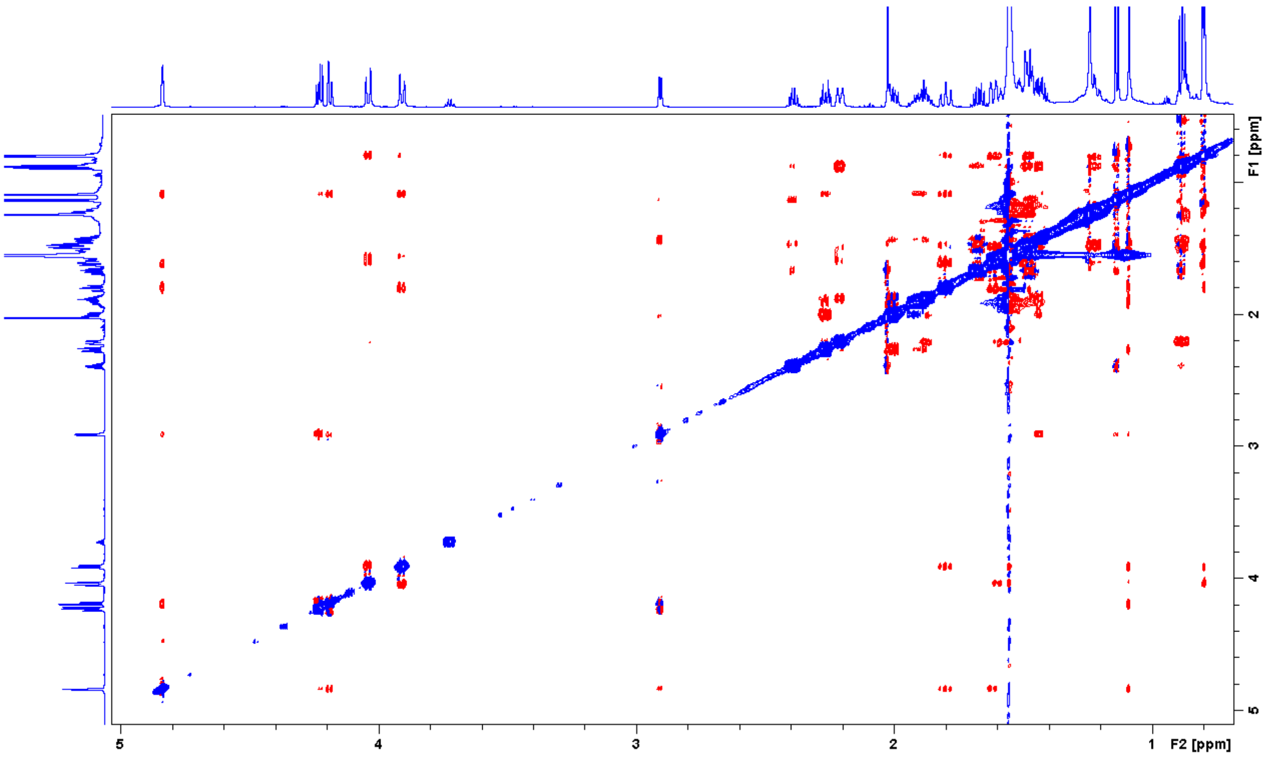

Figure S7. NOESY spectrum of **1** (700 MHz, $CDCl_3$).

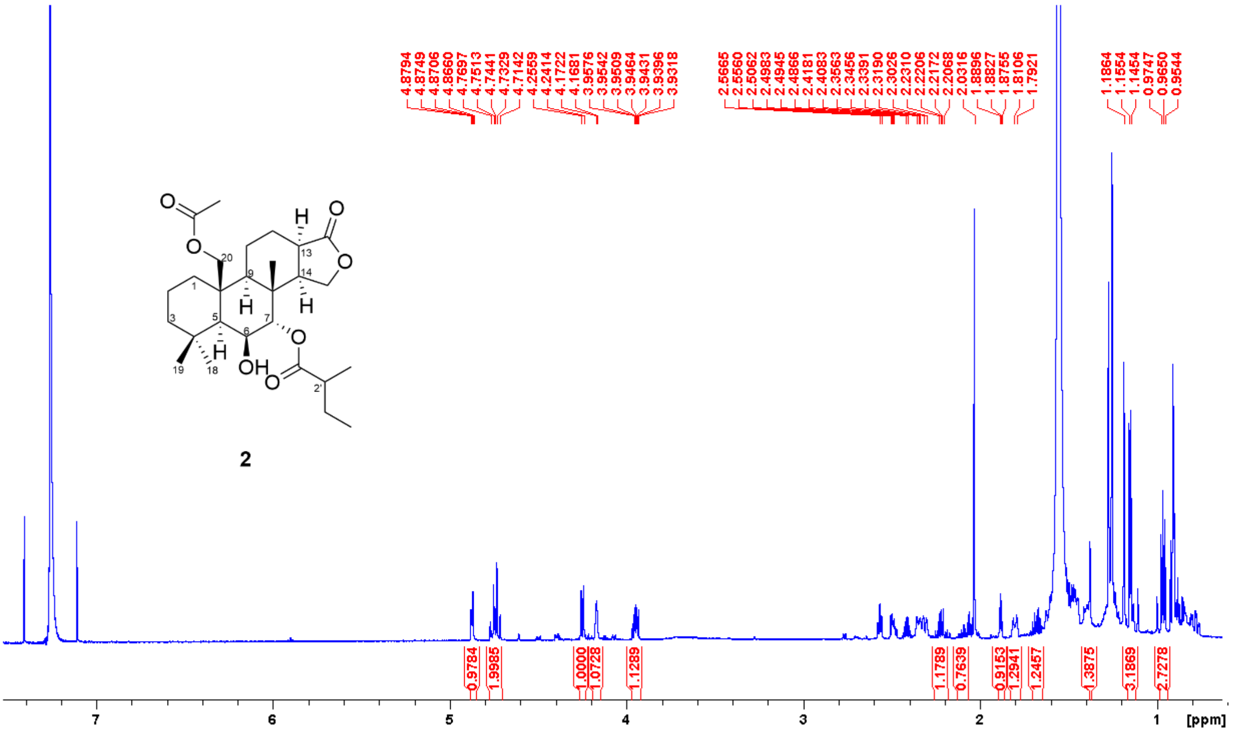

Figure S8. ^{1}H NMR spectrum of **2** (700 MHz, $CDCl_3$).

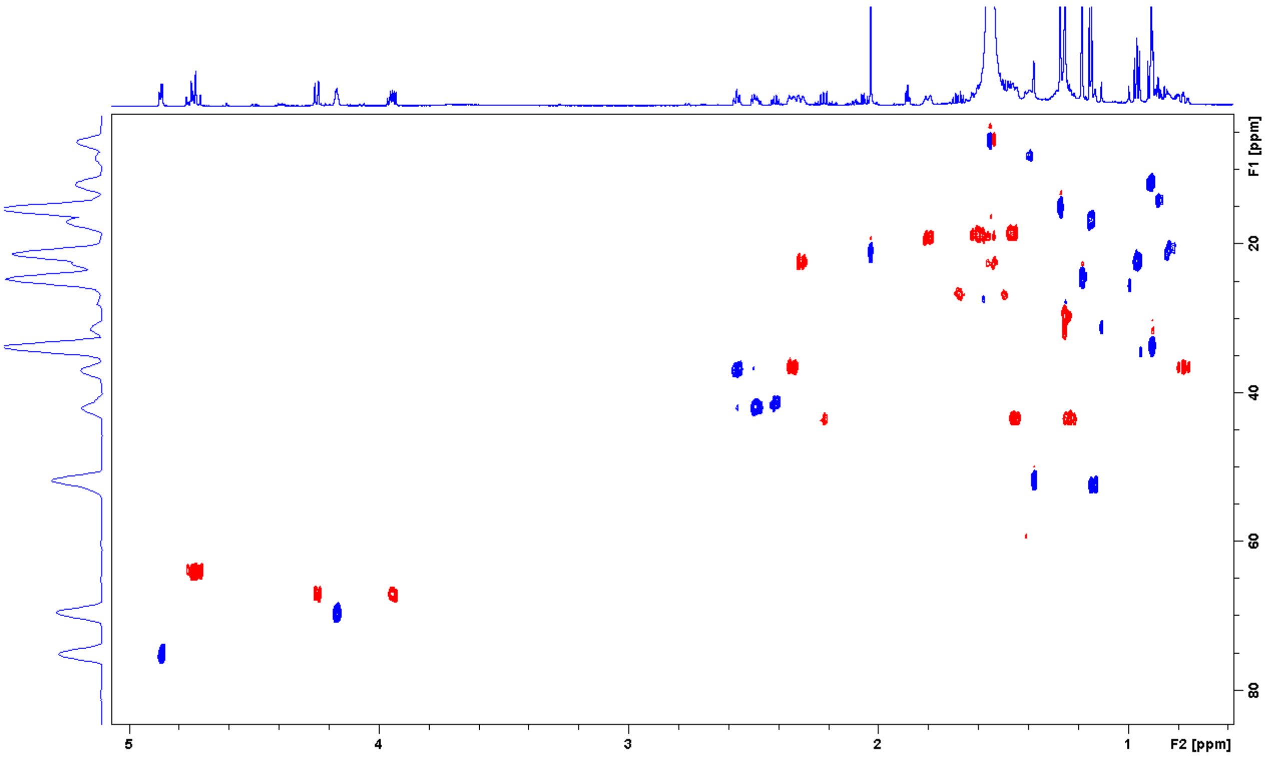

Figure S9. HSQC spectrum of **2** (700 MHz, $CDCl_3$).

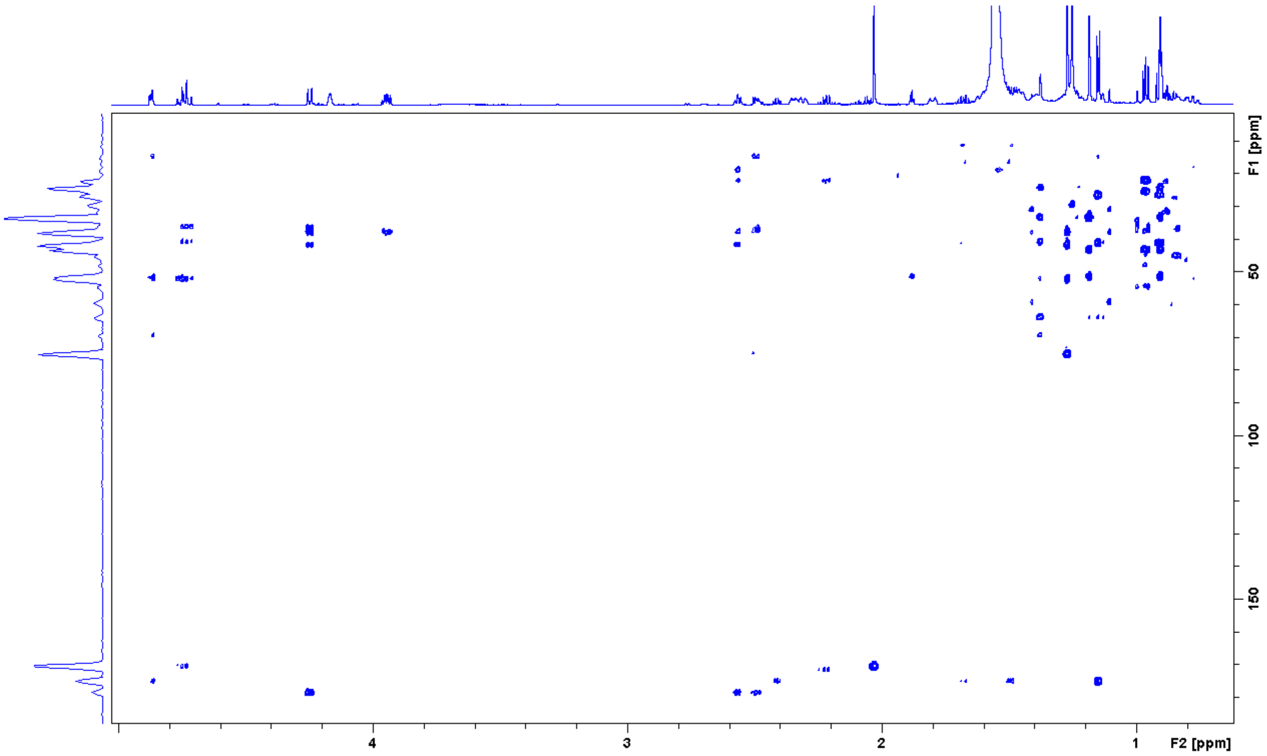

Figure S10. HMBC spectrum of **2** (700 MHz, $CDCl_3$).

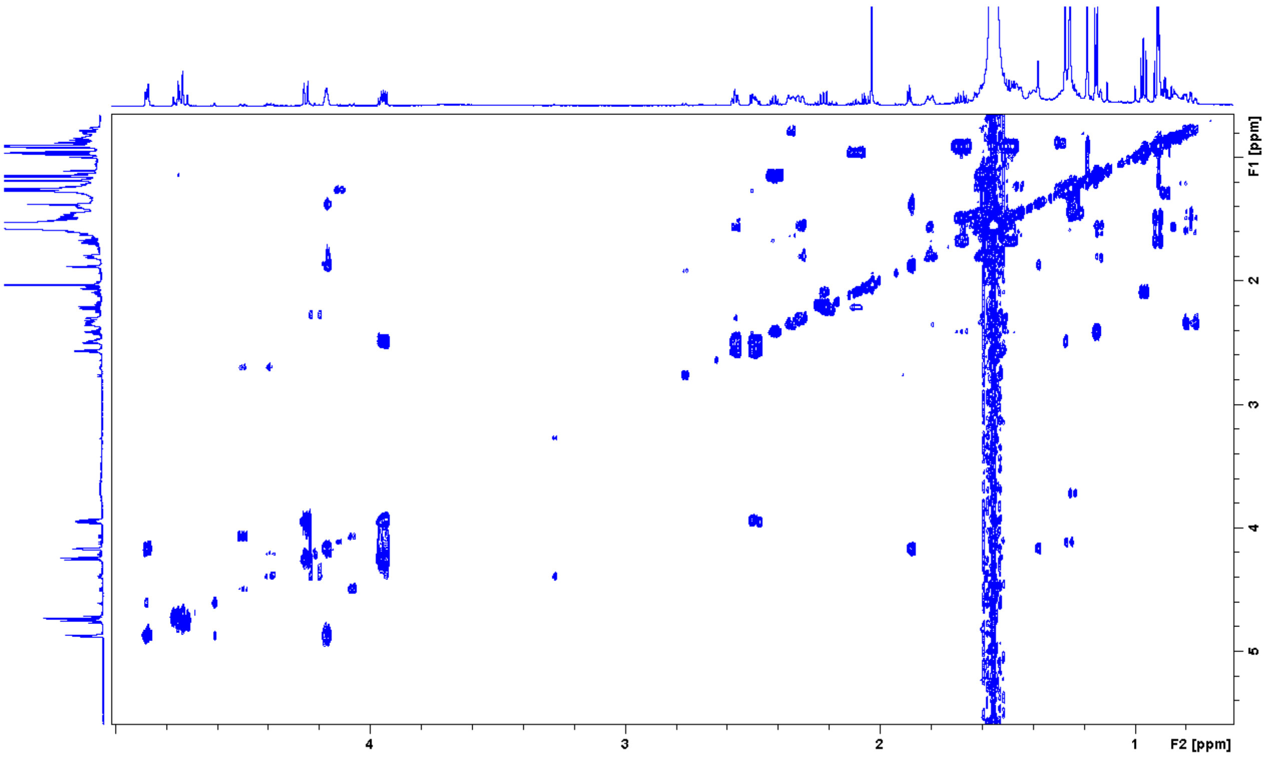

Figure S11. COSY spectrum of **2** (700 MHz, $CDCl_3$).

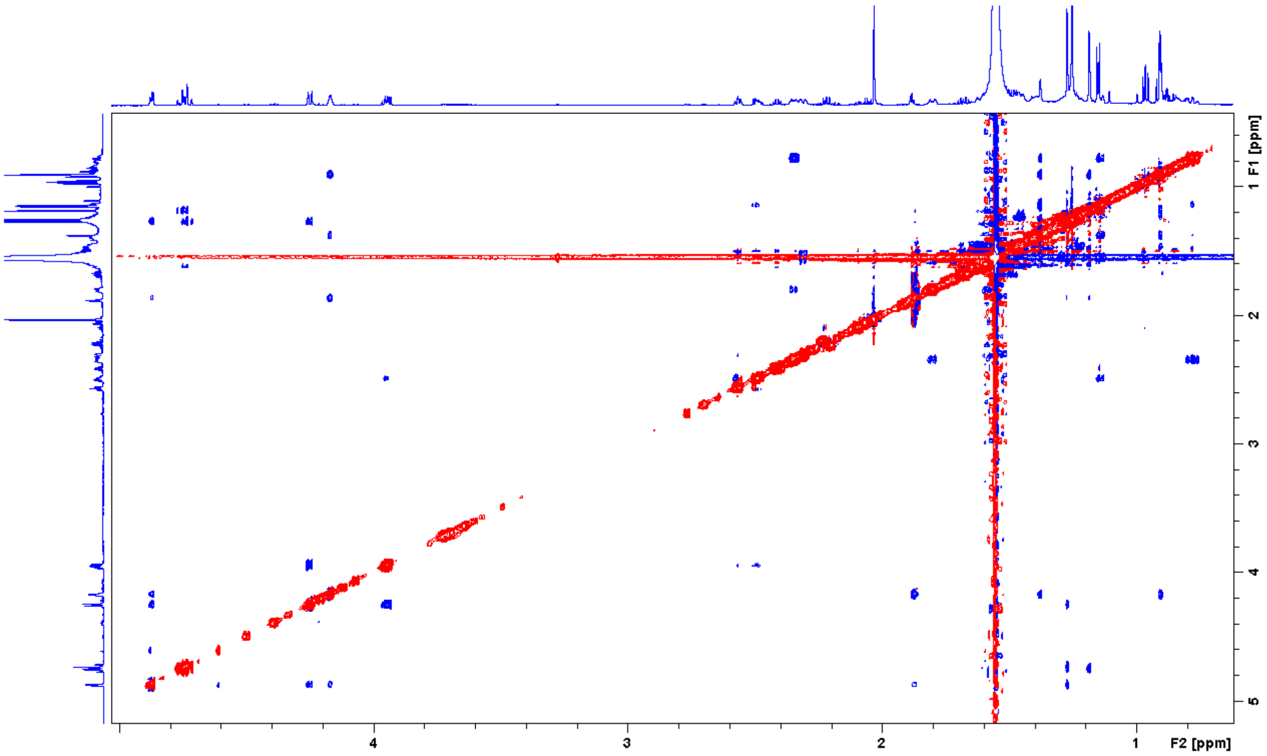

Figure S12. NOESY spectrum of **2** (700 MHz, $CDCl_3$).

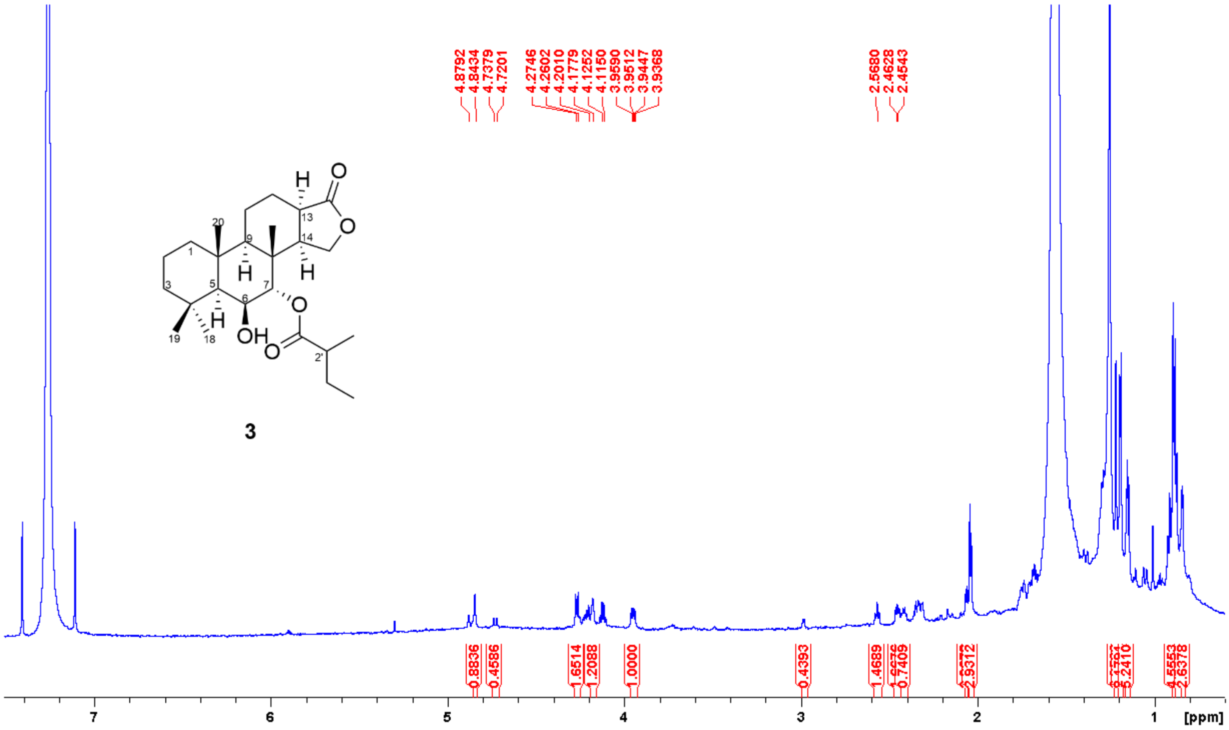

Figure S13. ^{1}H NMR spectrum of **3** (700 MHz, $CDCl_3$), collected in a Shigemi NMR tube.

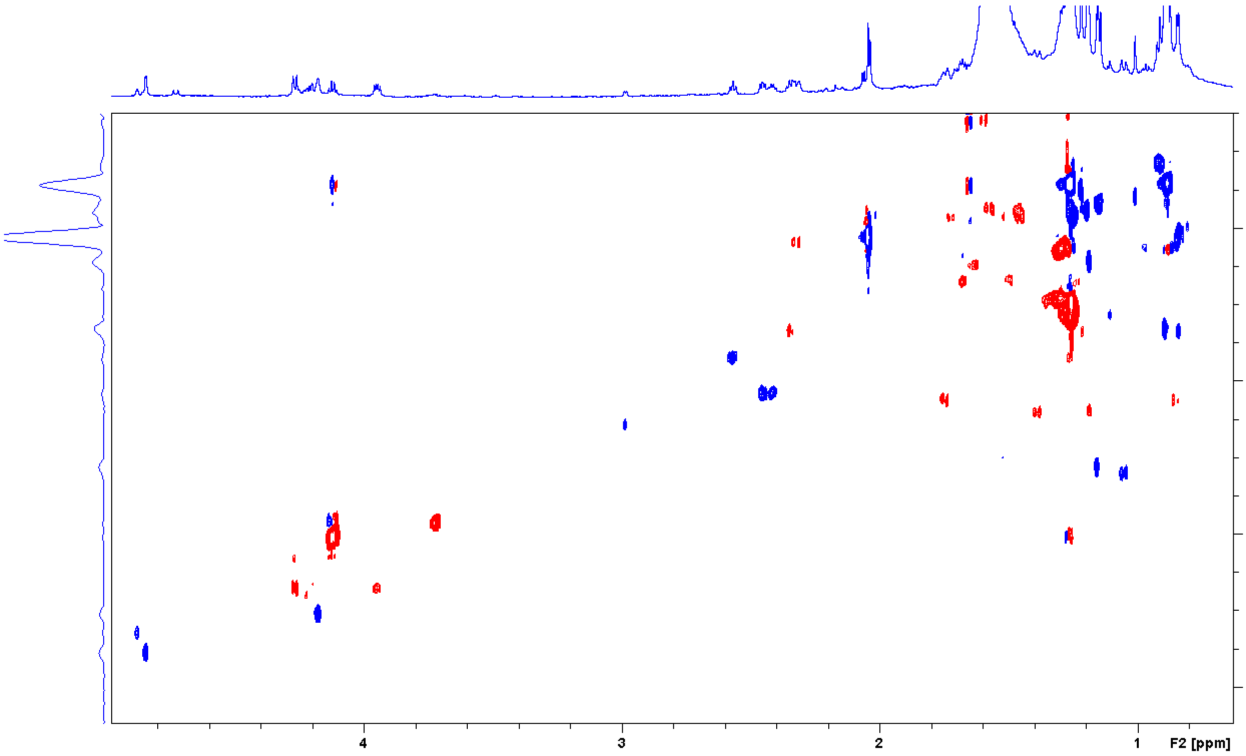

Figure S14. HSQC spectrum of **3** (700 MHz, $CDCl_3$), collected in a Shigemi NMR tube.

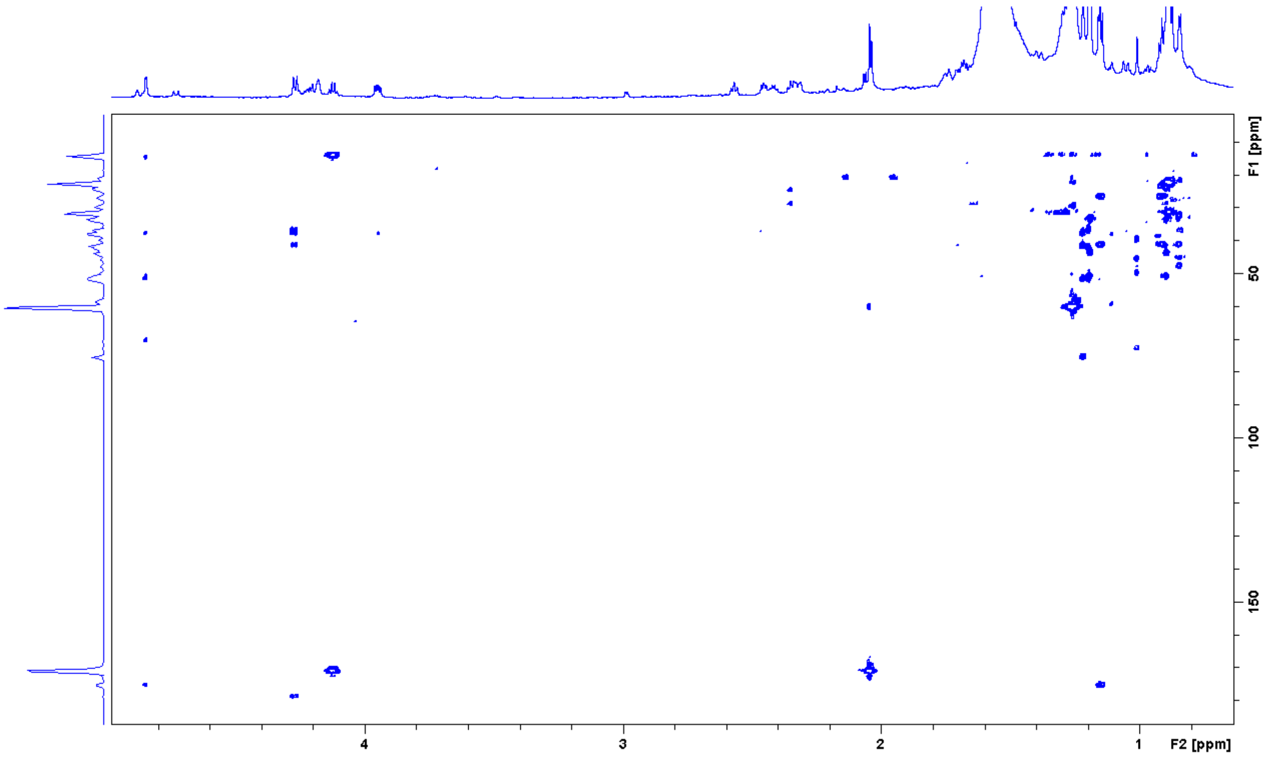

Figure S15. HMBC spectrum of **3** (700 MHz, $CDCl_3$), collected in a Shigemi NMR tube.

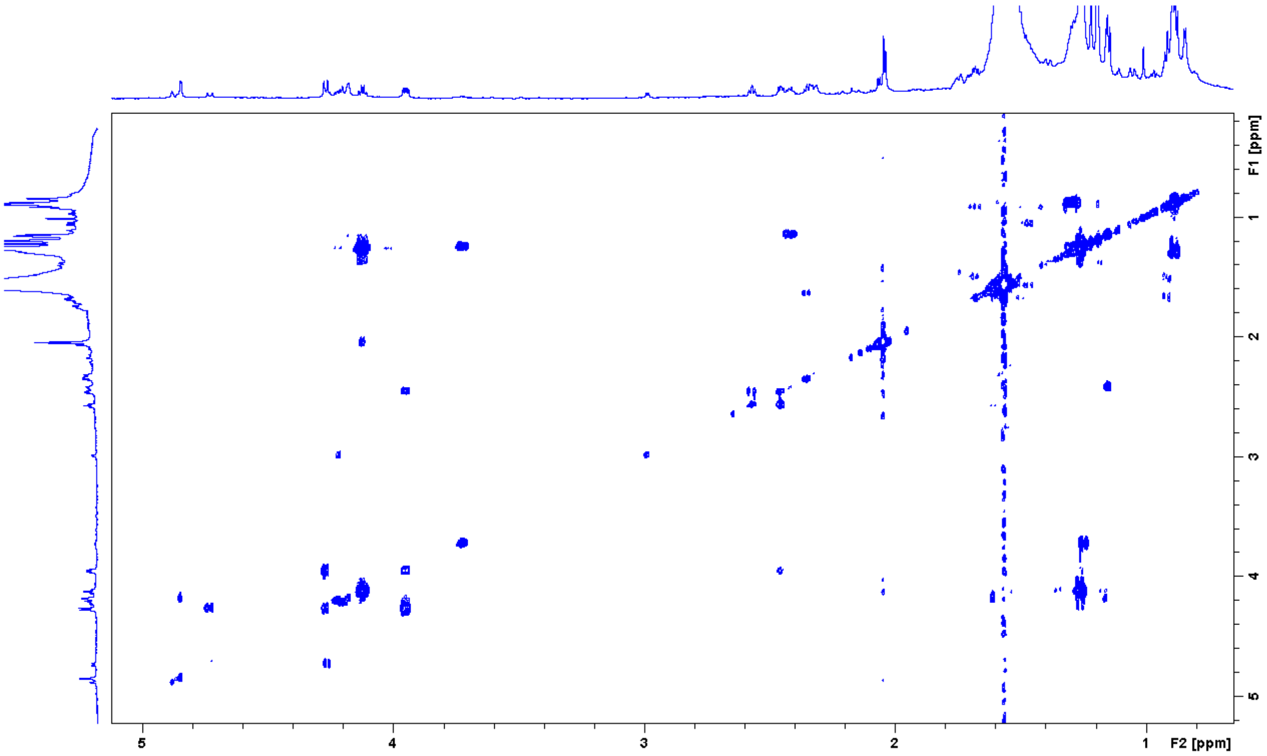

Figure S16. COSY spectrum of **3** (700 MHz, $CDCl_3$), collected in a Shigemi NMR tube.

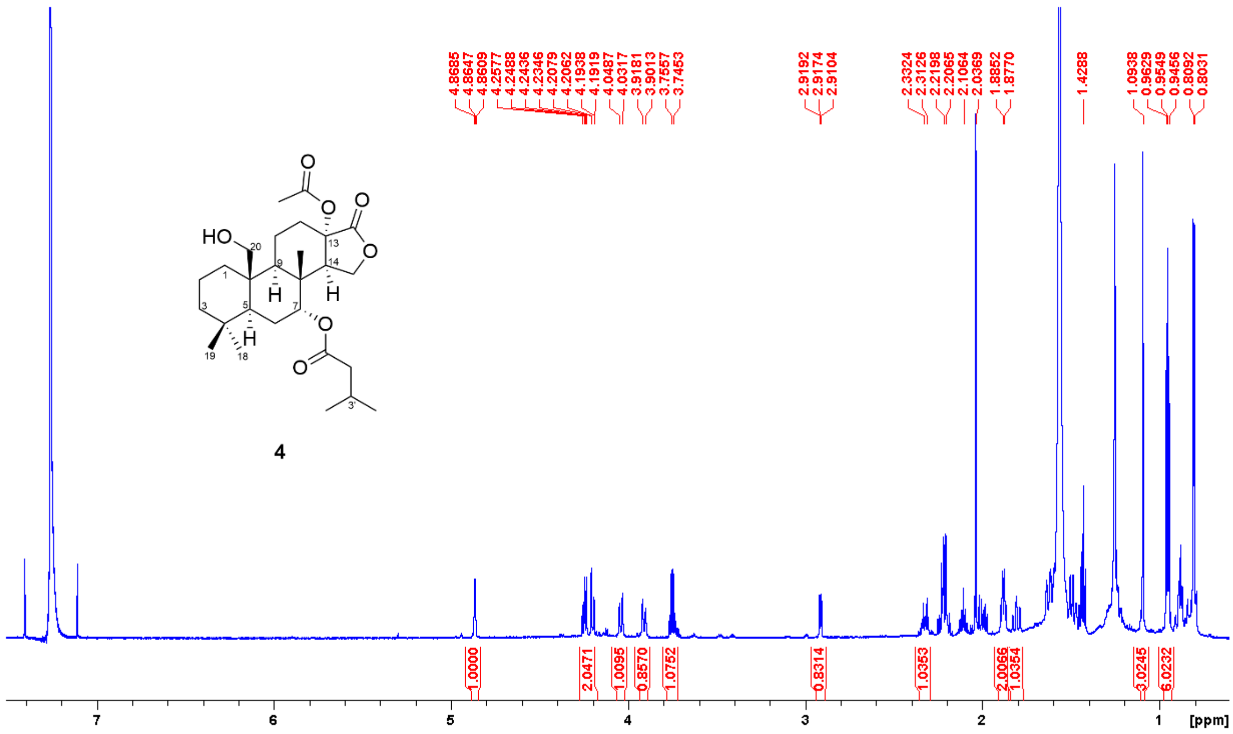

Figure S17. ^{1}H NMR spectrum of **4** (700 MHz, $CDCl_3$).

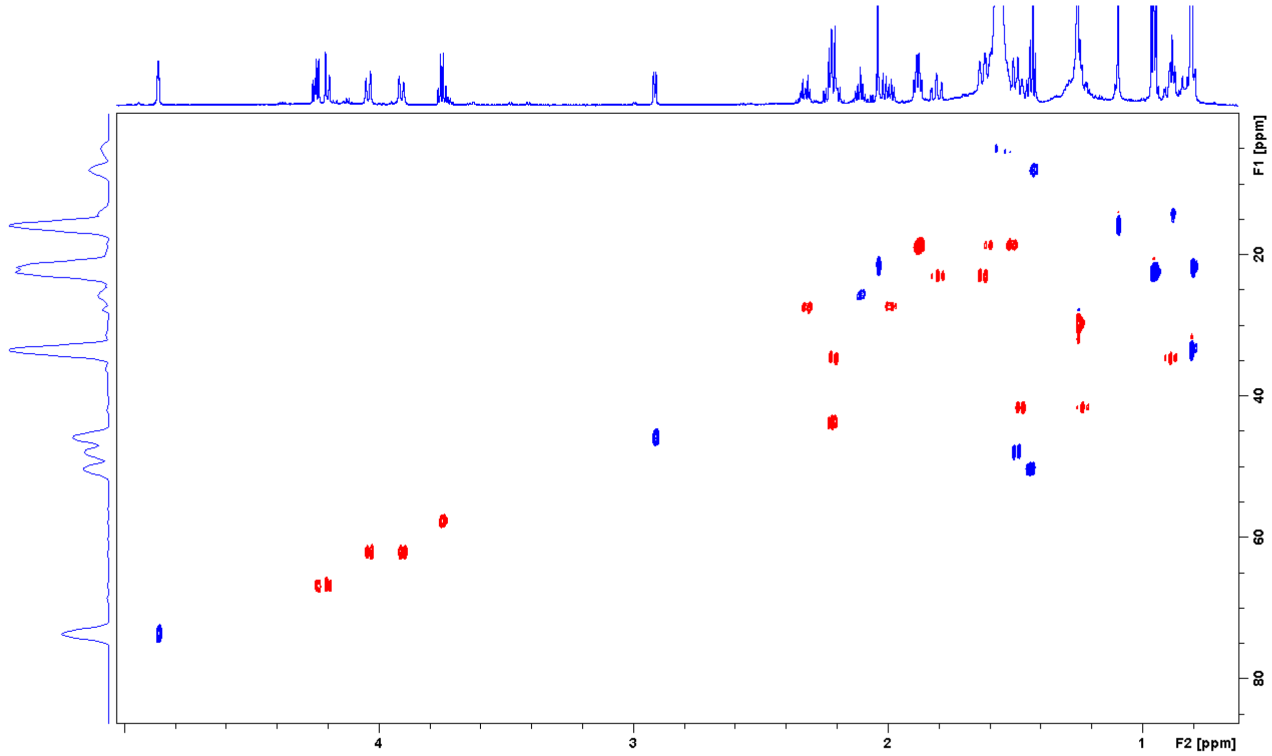

Figure S18. HSQC spectrum of **4** (700 MHz, $CDCl_3$).

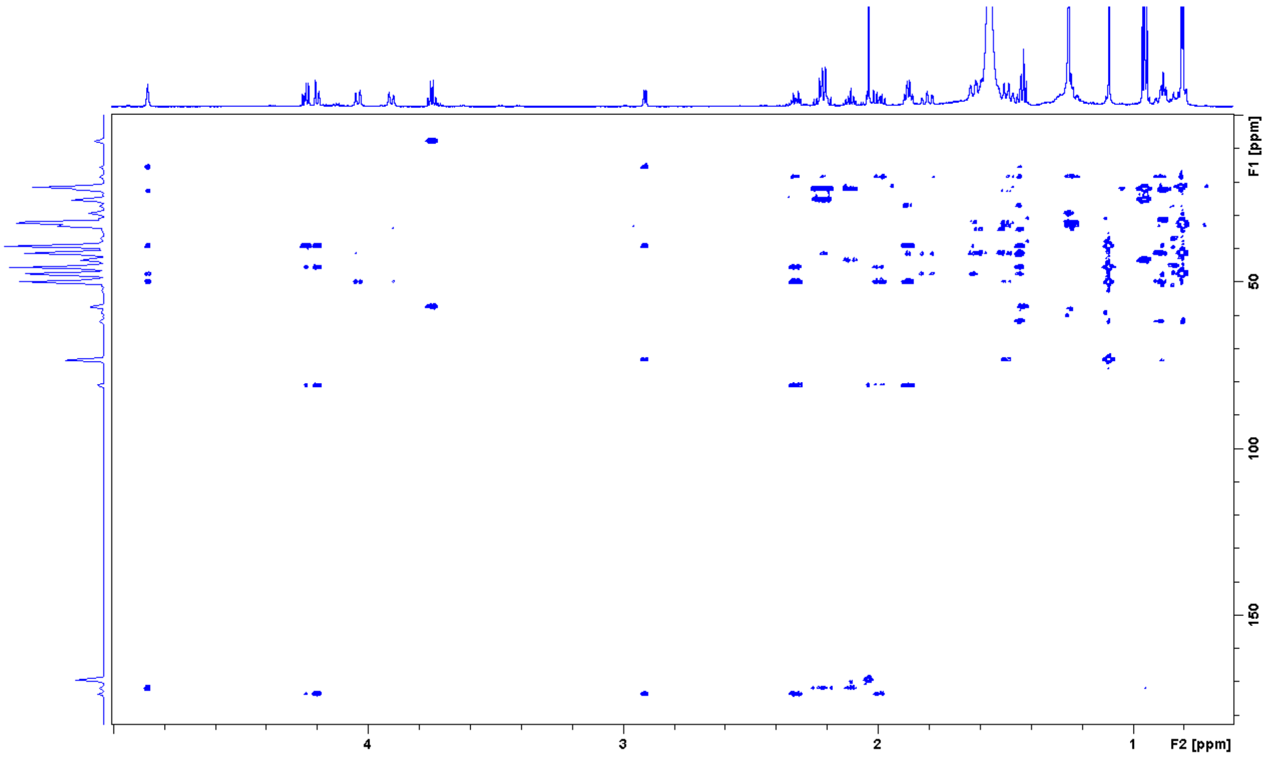

Figure S19. HMBC spectrum of **4** (700 MHz, $CDCl_3$).

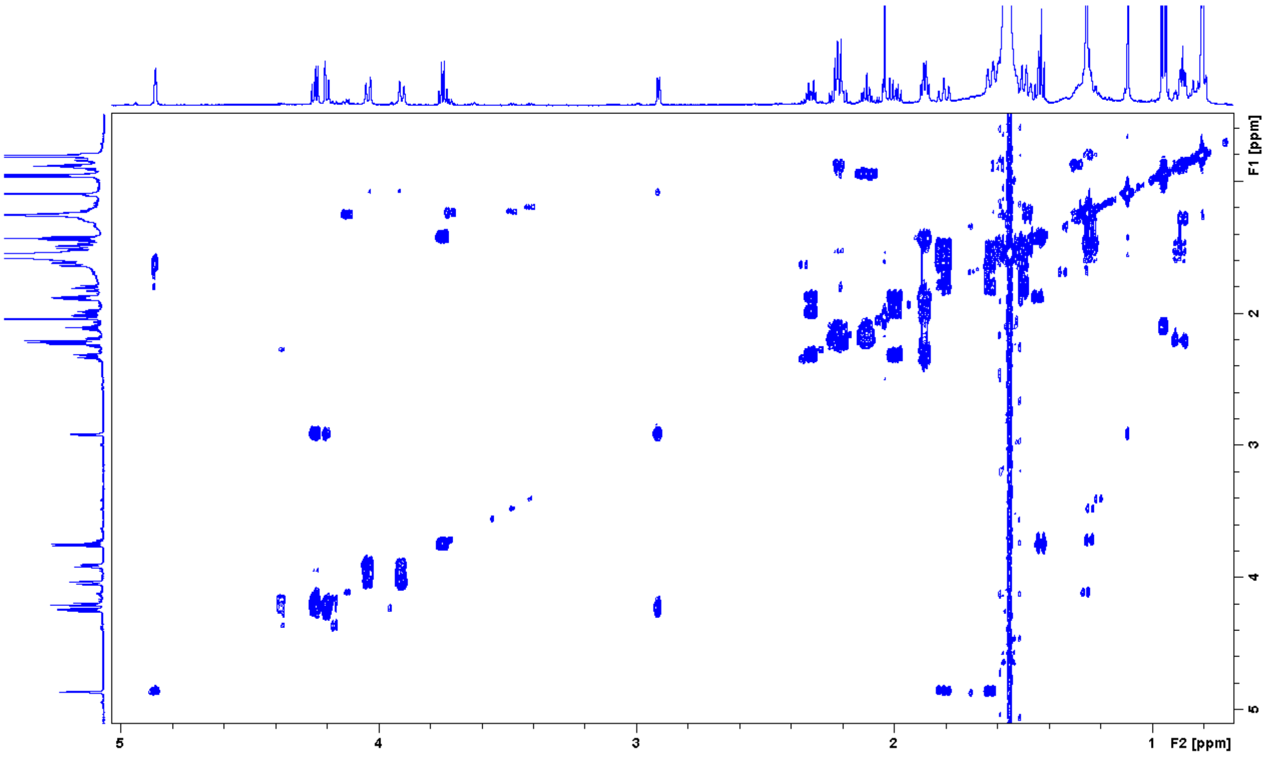

Figure S20. COSY spectrum of **4** (700 MHz, $CDCl_3$).

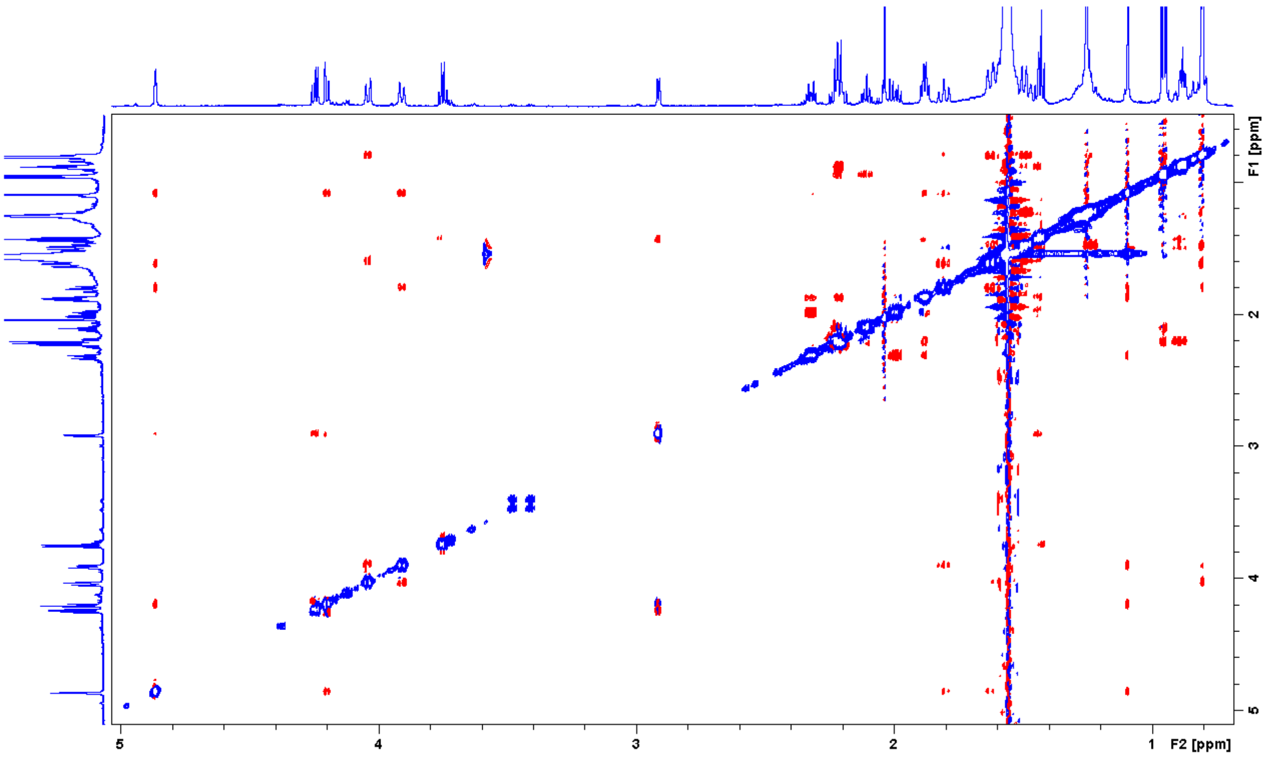

Figure S21. NOESY spectrum of **4** (700 MHz, $CDCl_3$).

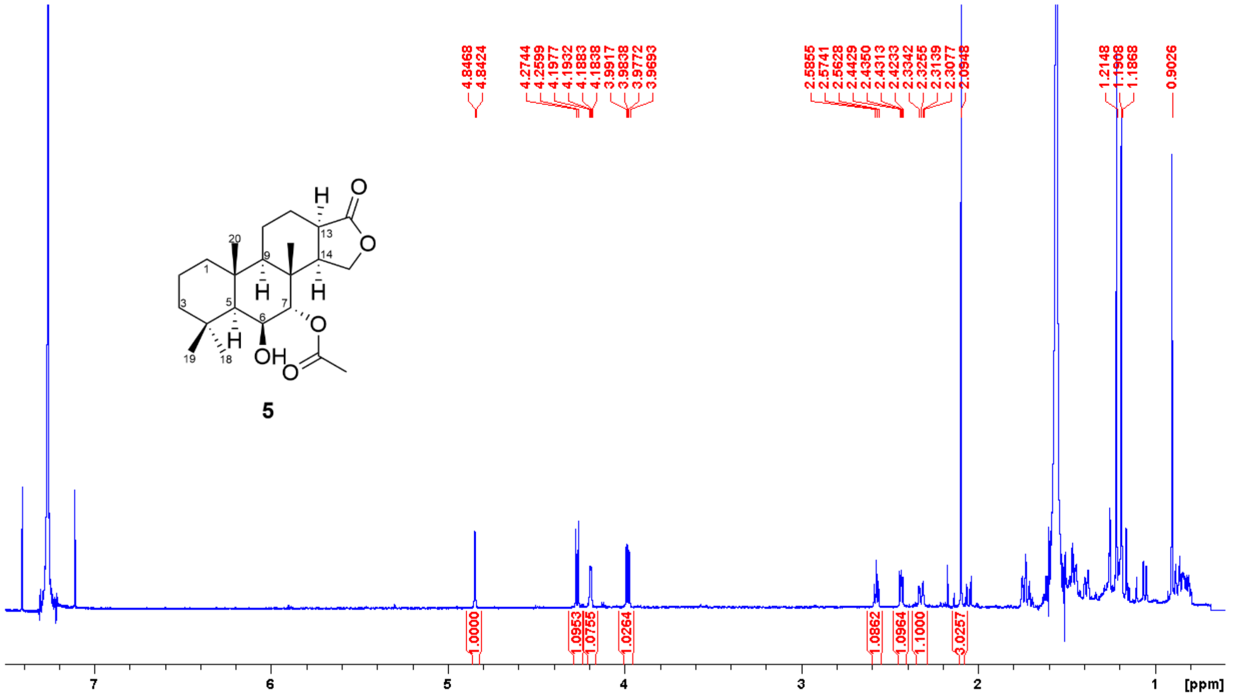

Figure S22. ^{1}H NMR spectrum of **5** (700 MHz, $CDCl_3$).

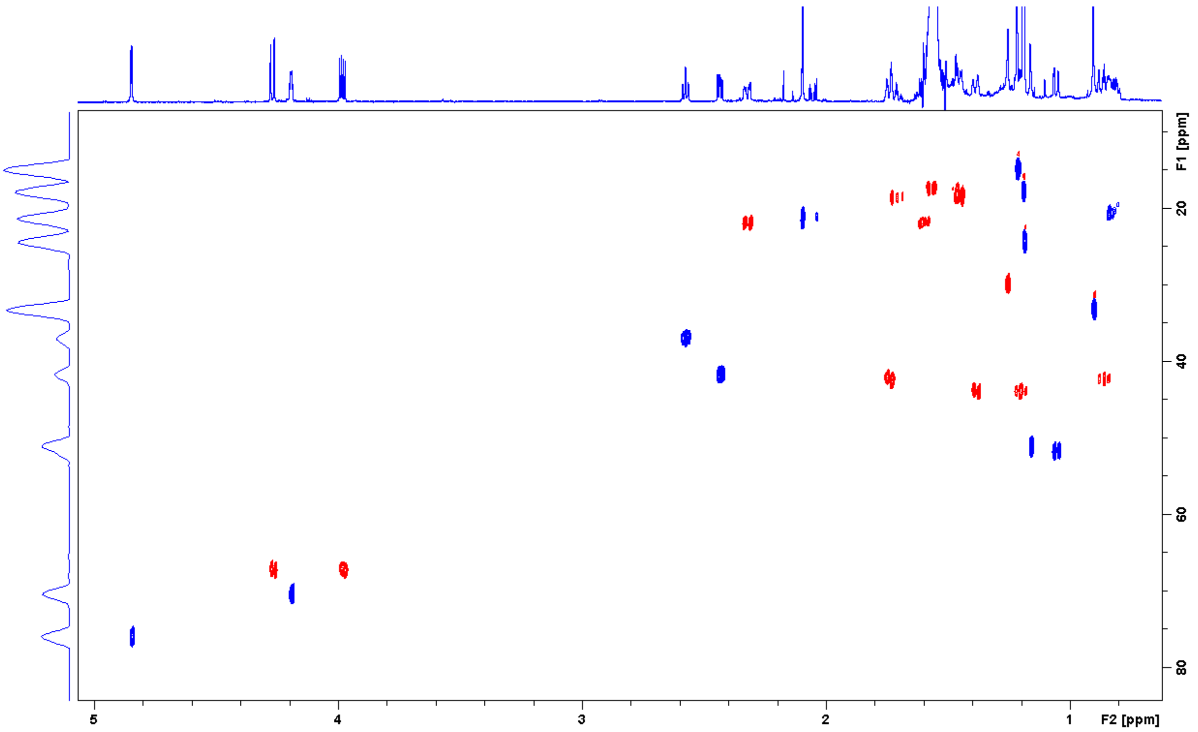

Figure S23. HSQC spectrum of **5** (700 MHz, $CDCl_3$).

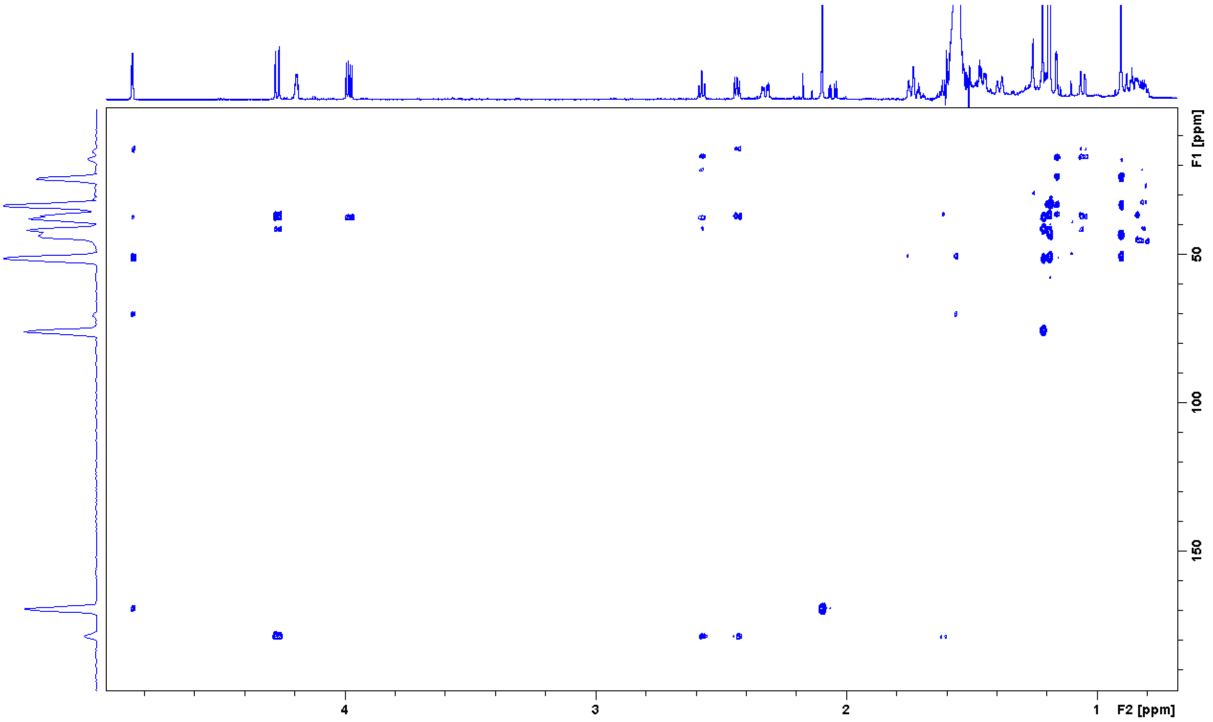

Figure S24. HMBC spectrum of **5** (700 MHz, $CDCl_3$).

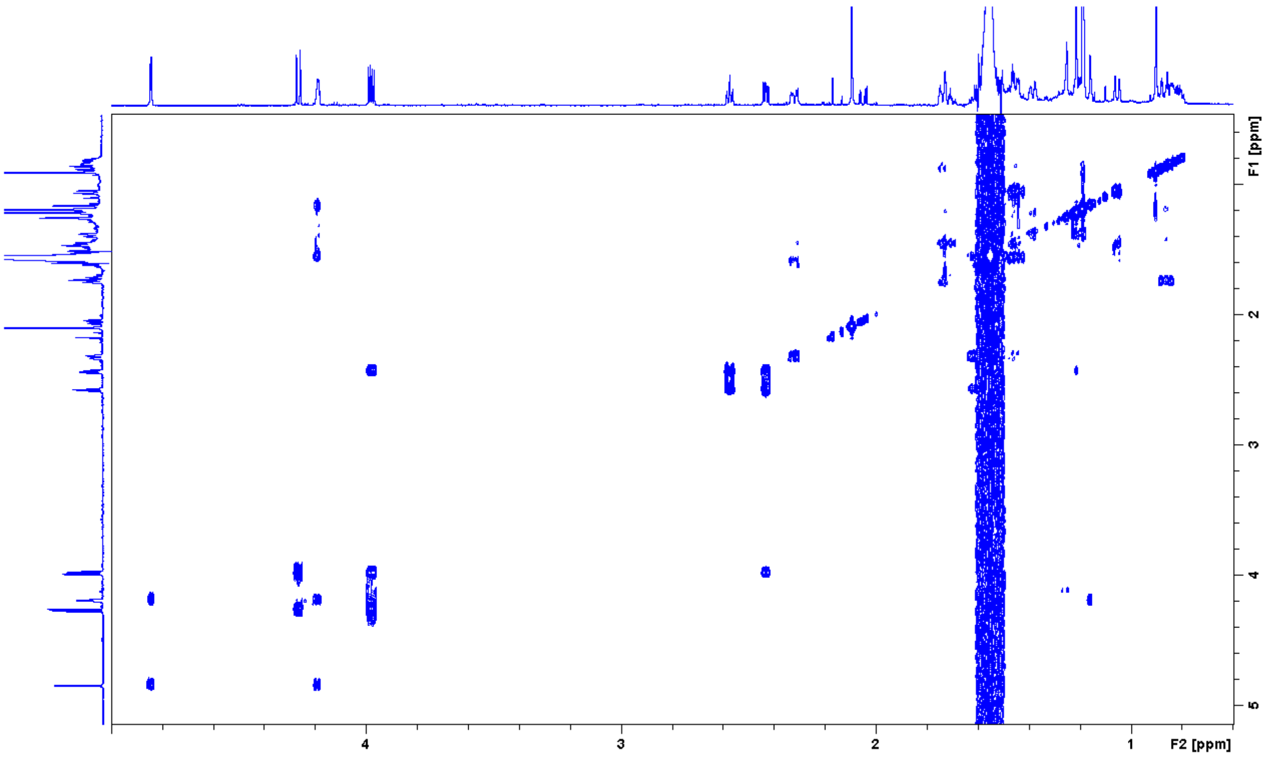

Figure S25. COSY spectrum of **5** (700 MHz, $CDCl_3$).

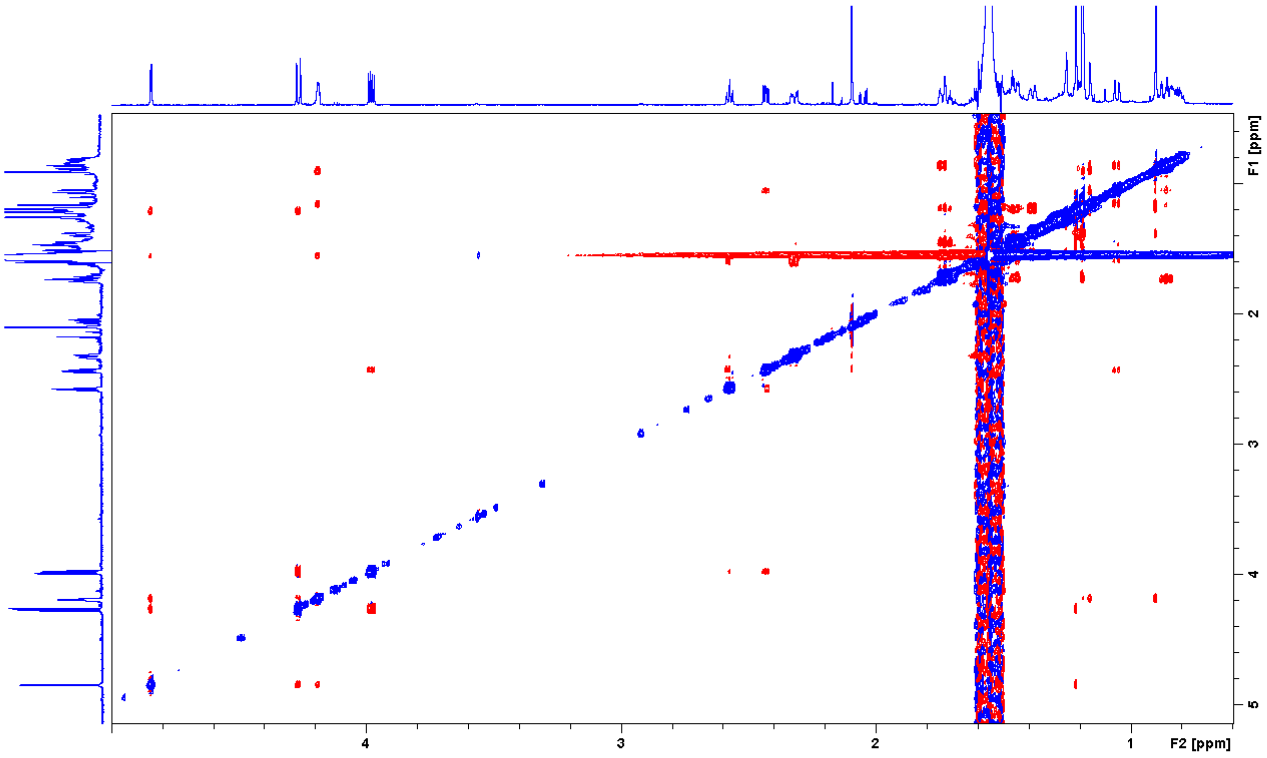

Figure S26. NOESY spectrum of **5** (700 MHz, $CDCl_3$).

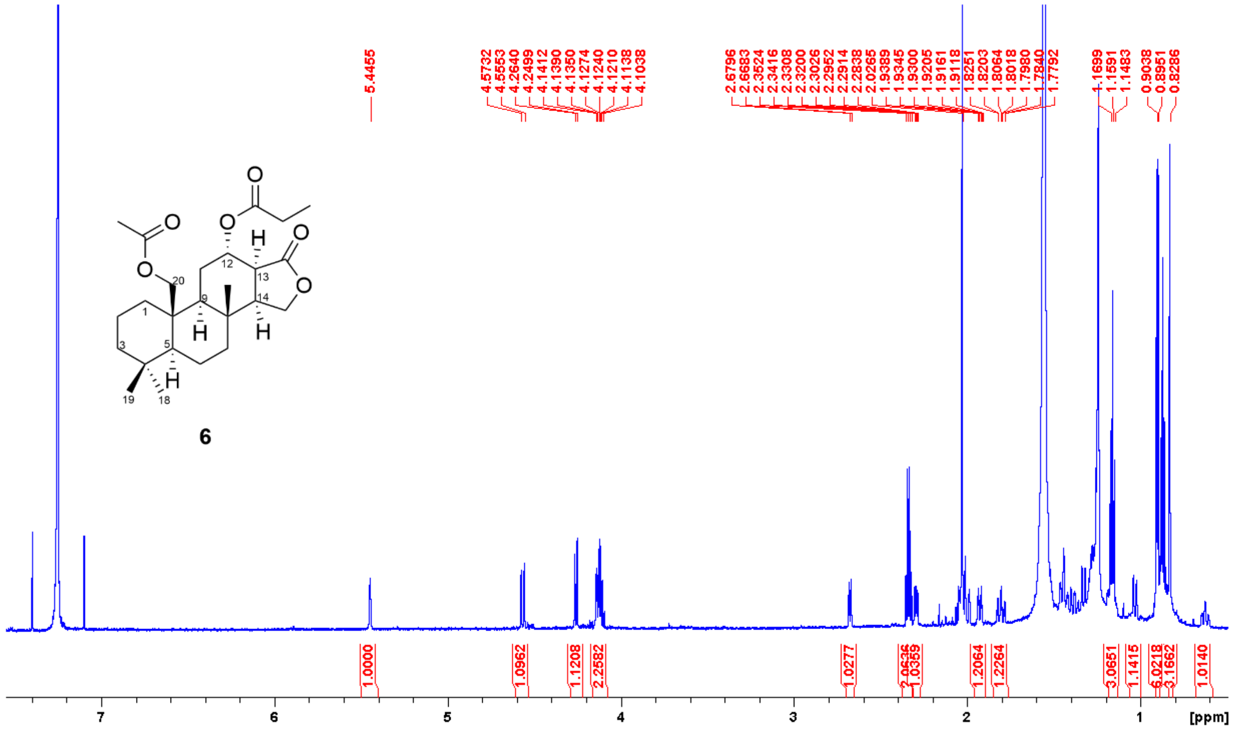

Figure S27. ^{1}H NMR spectrum of **6** (700 MHz, $CDCl_3$).

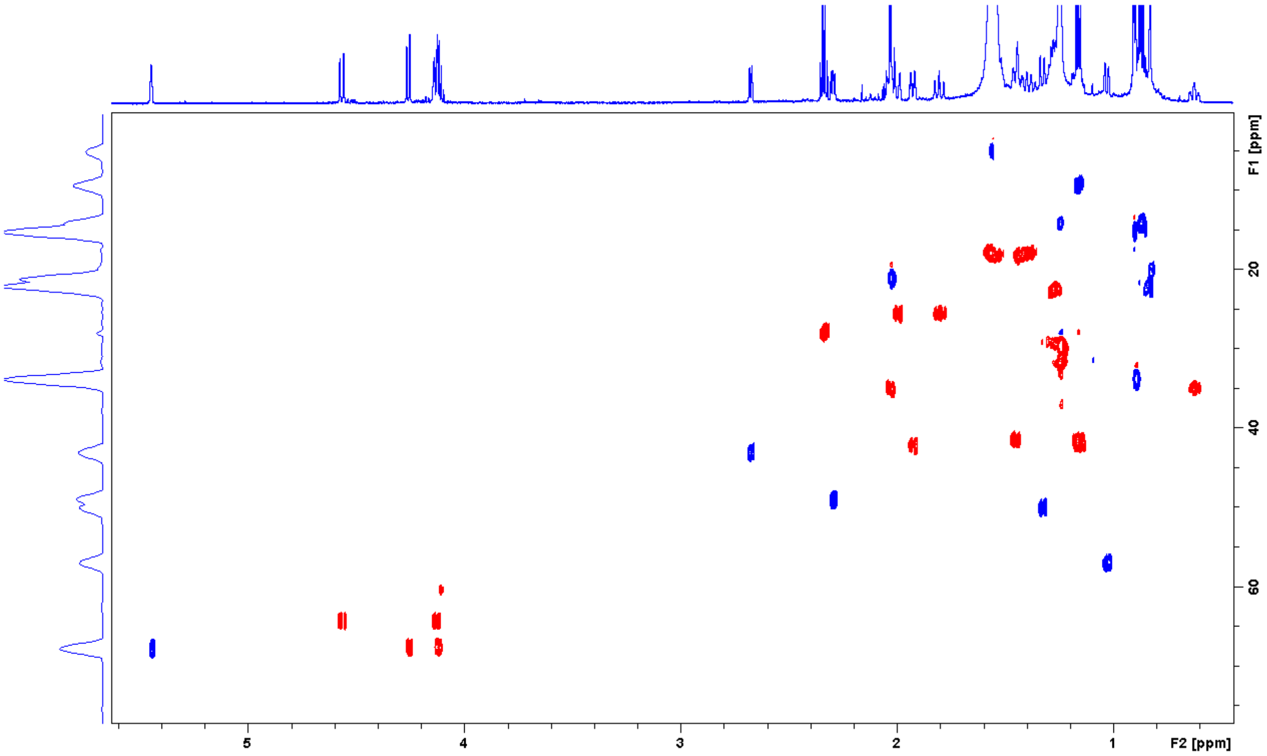

Figure S28. HSQC spectrum of **6** (700 MHz, $CDCl_3$).

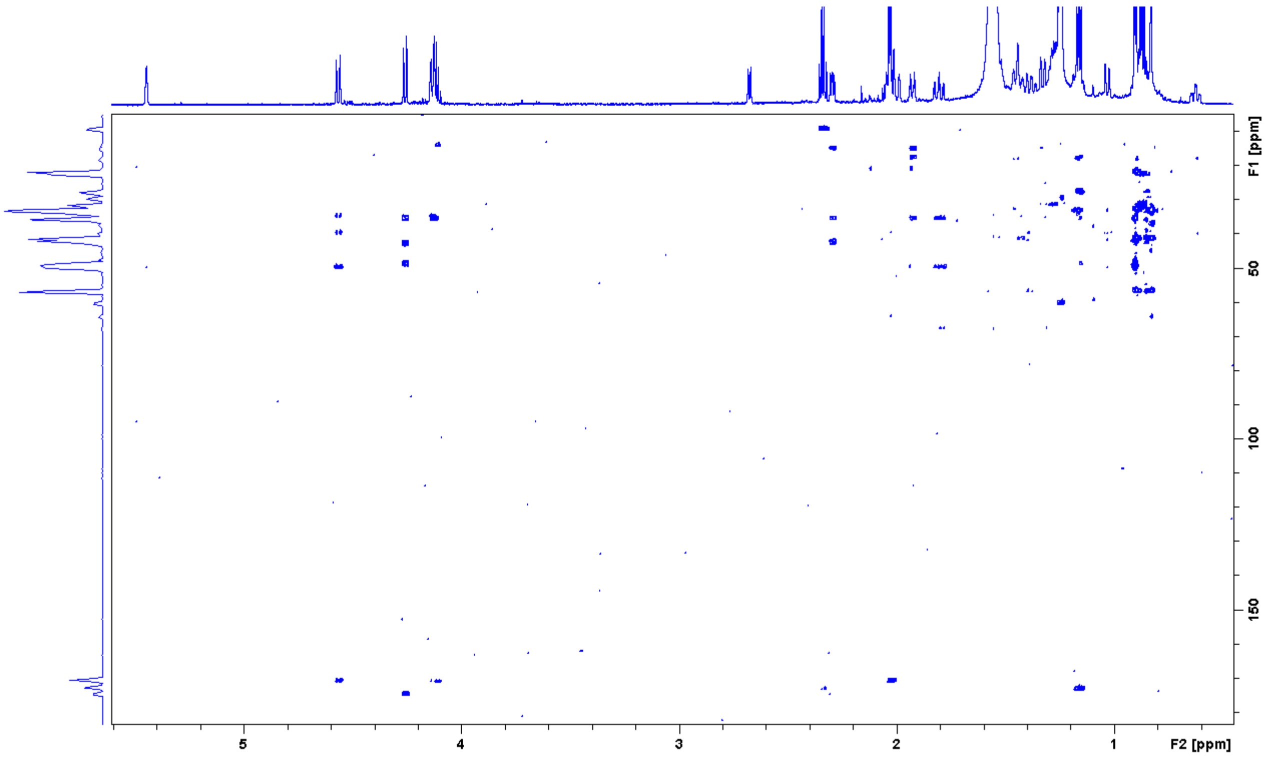

Figure S29. HMBC spectrum of **6** (700 MHz, $CDCl_3$).

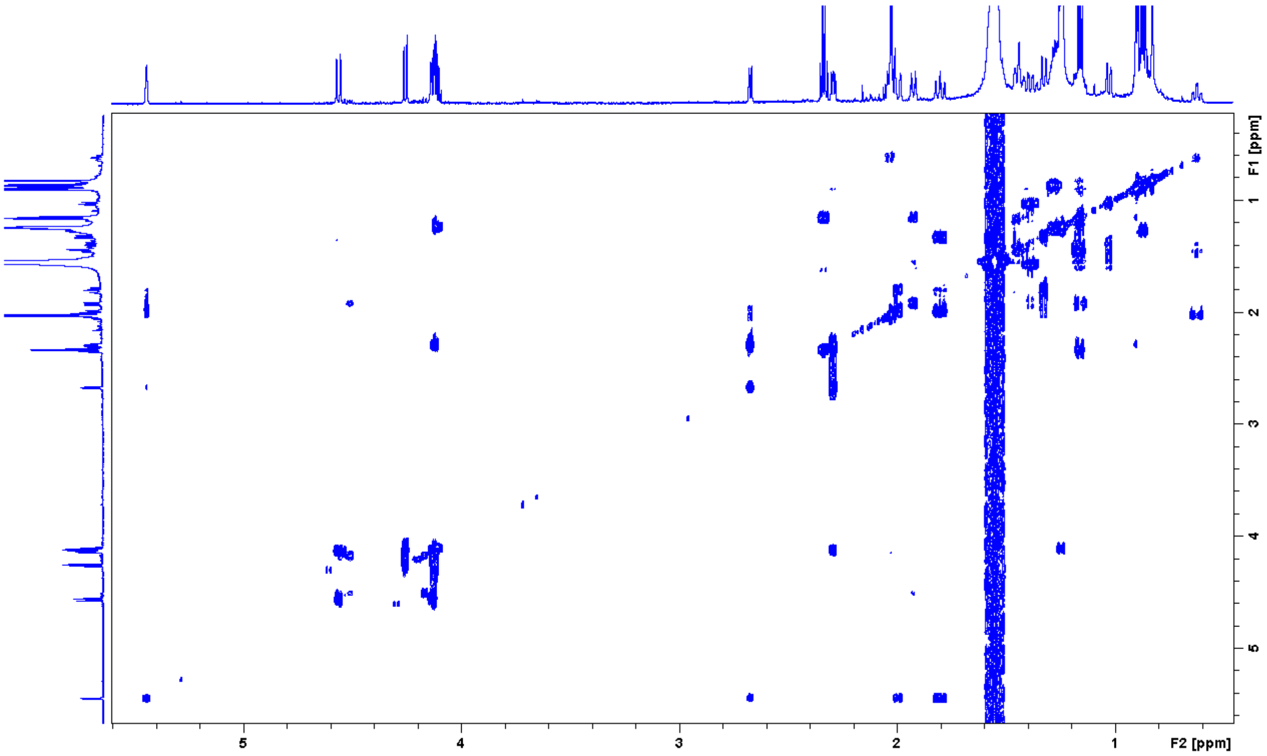

Figure S30. COSY spectrum of **6** (700 MHz, $CDCl_3$).

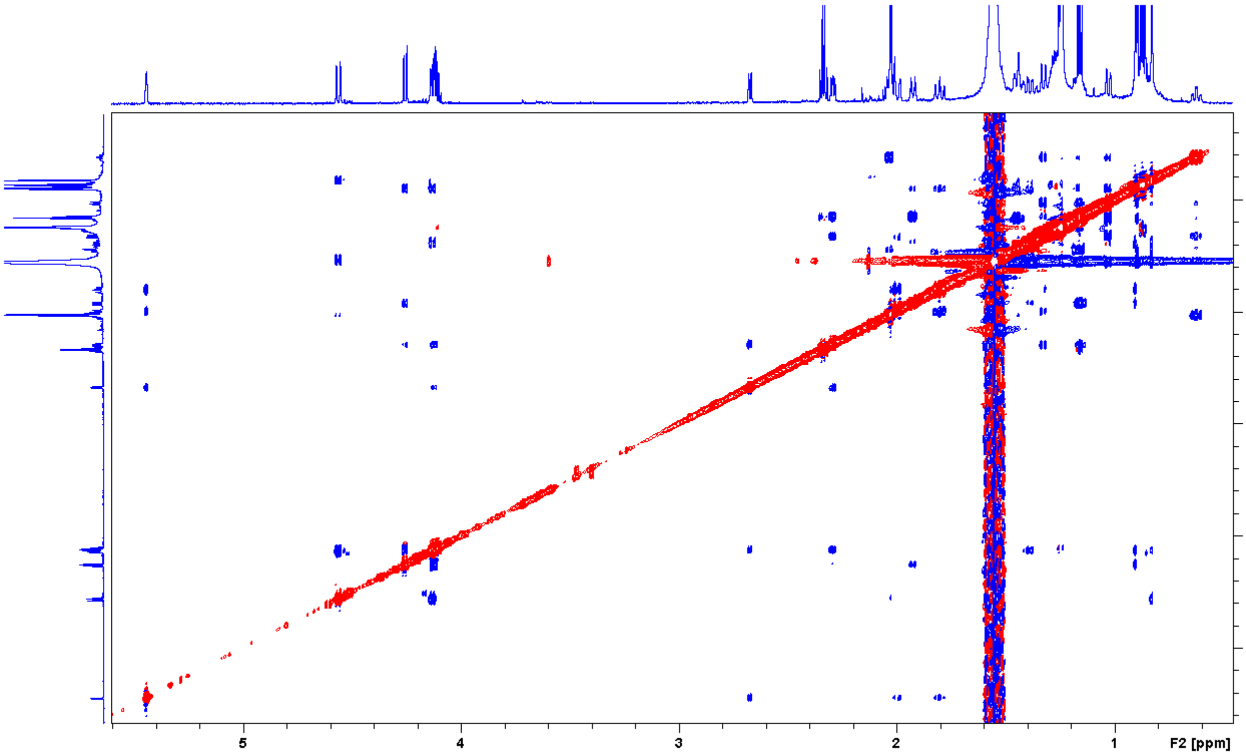

Figure S31. NOESY spectrum of **6** (700 MHz, $CDCl_3$).

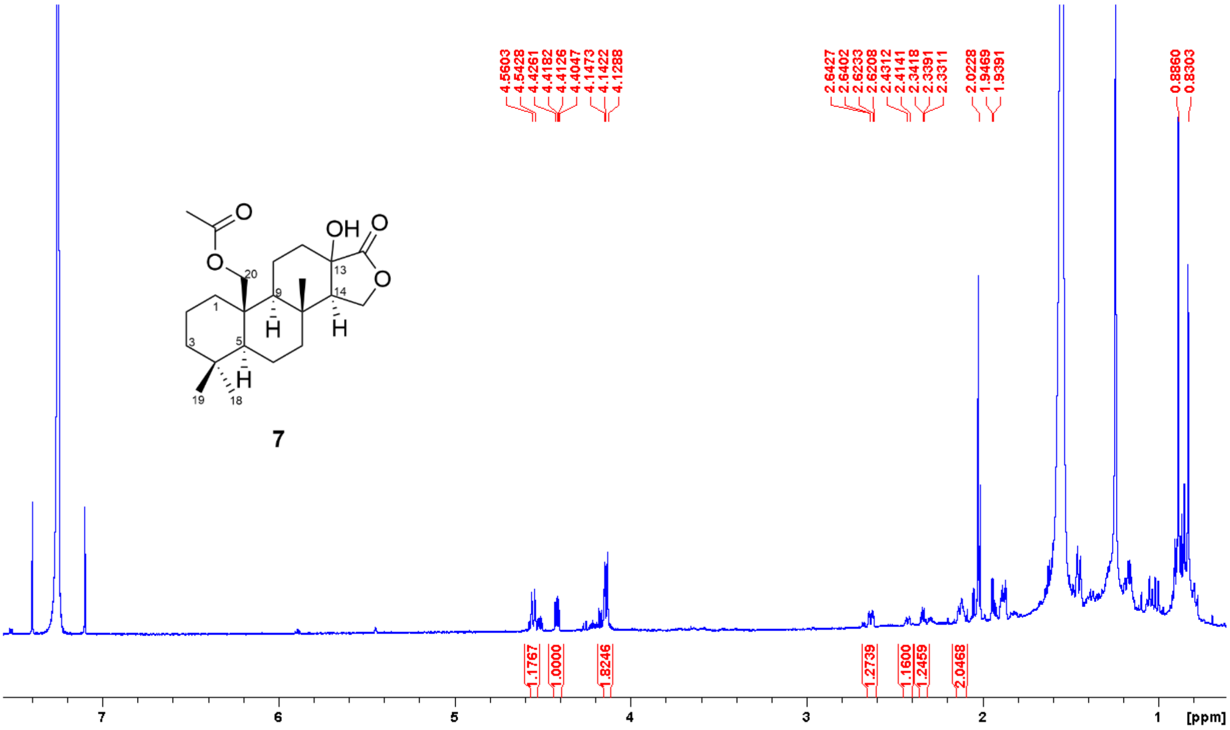

Figure S32. ^{1}H NMR spectrum of **7** (700 MHz, $CDCl_3$).

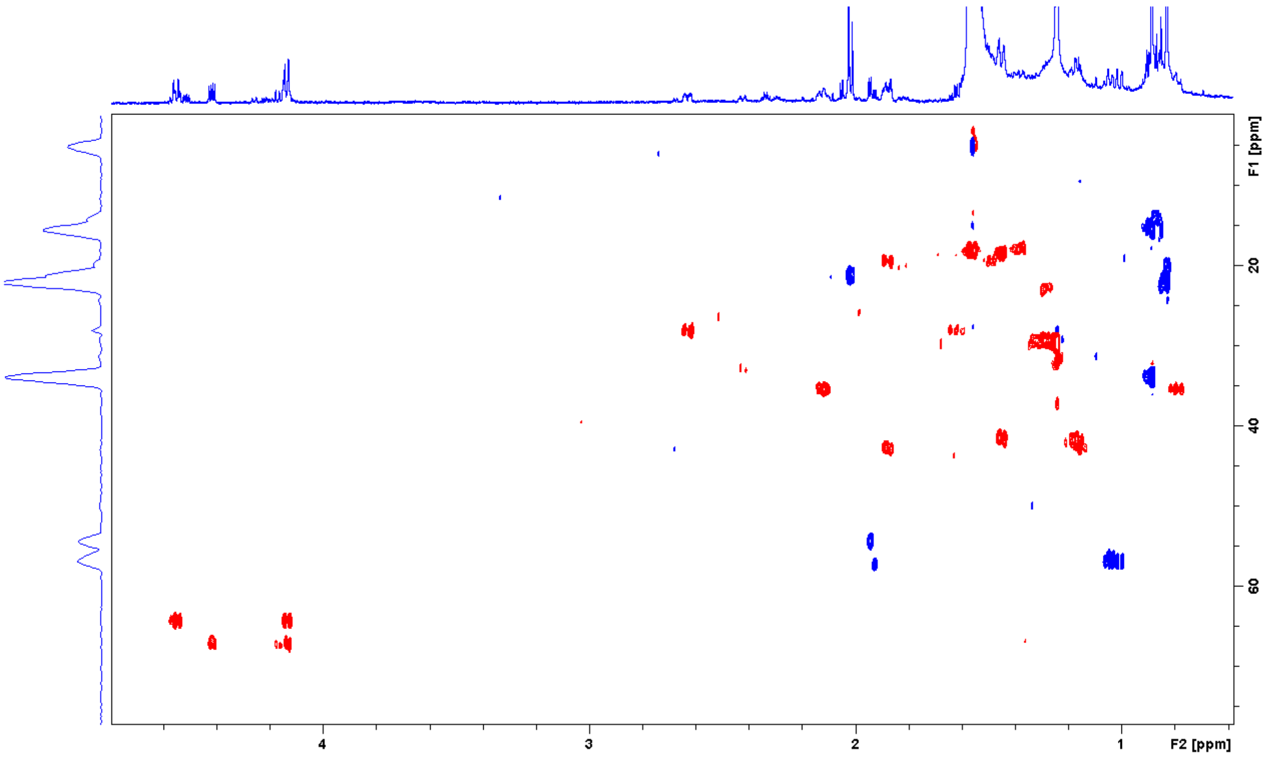

Figure S33. HSQC spectrum of **7** (700 MHz, $CDCl_3$).

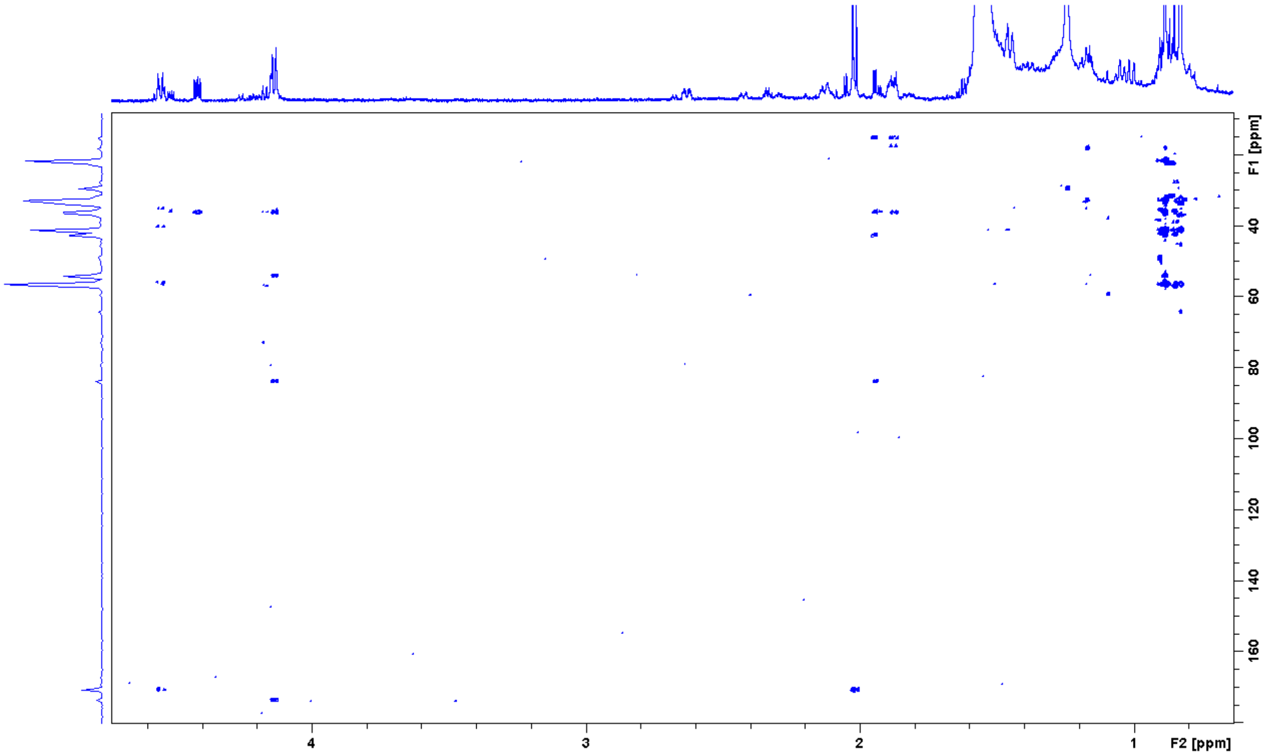

Figure S34. HMBC spectrum of **7** (700 MHz, $CDCl_3$).

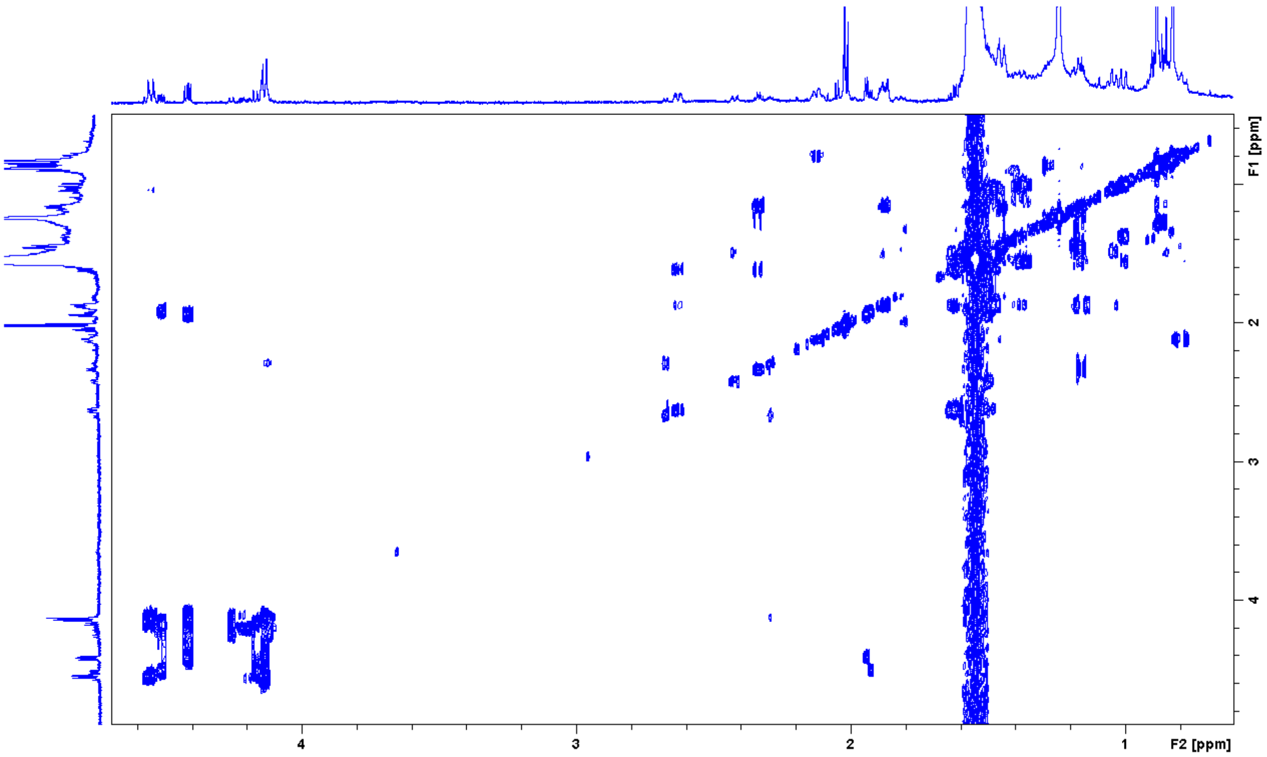

Figure S35. COSY spectrum of **7** (700 MHz, $CDCl_3$).

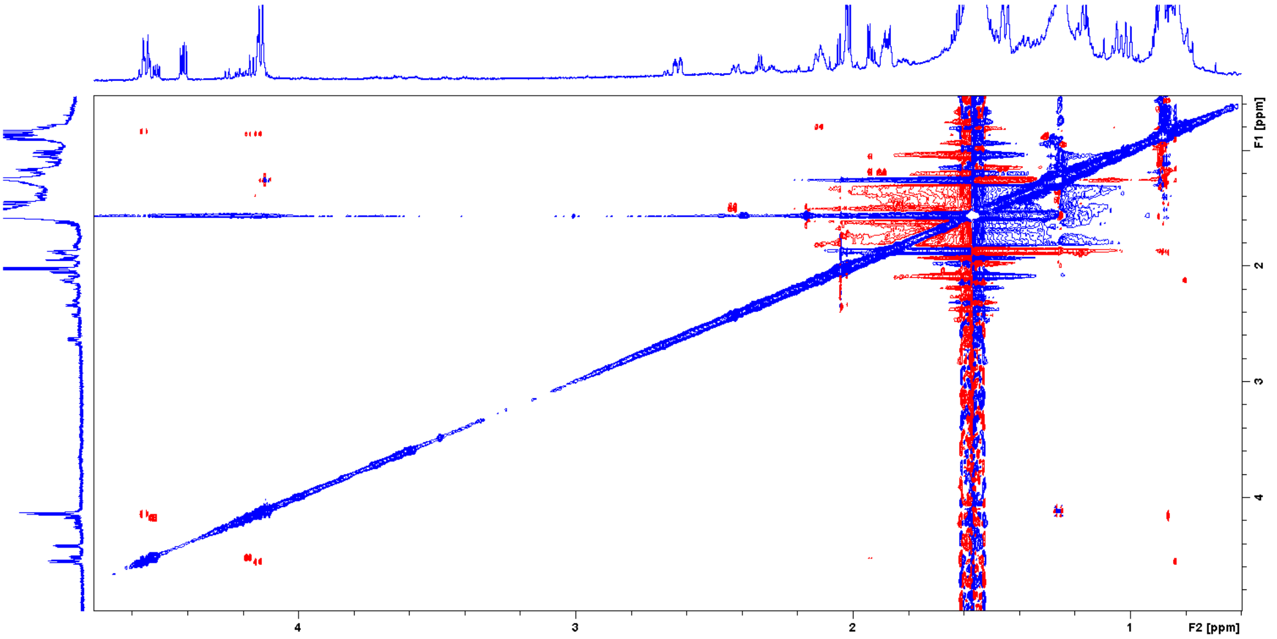

Figure S36. NOSEY spectrum of **7** (700 MHz, $CDCl_3$).

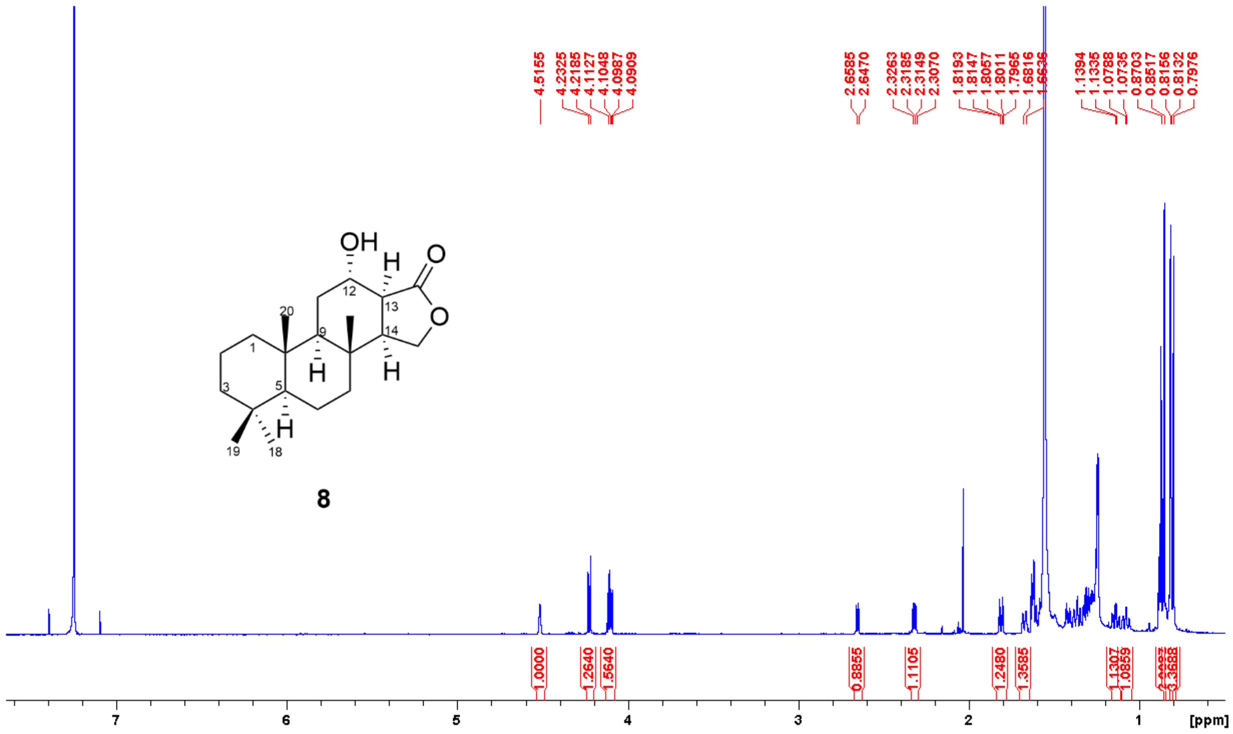

Figure S37. ^{1}H NMR spectrum of **8** (700 MHz, $CDCl_3$).

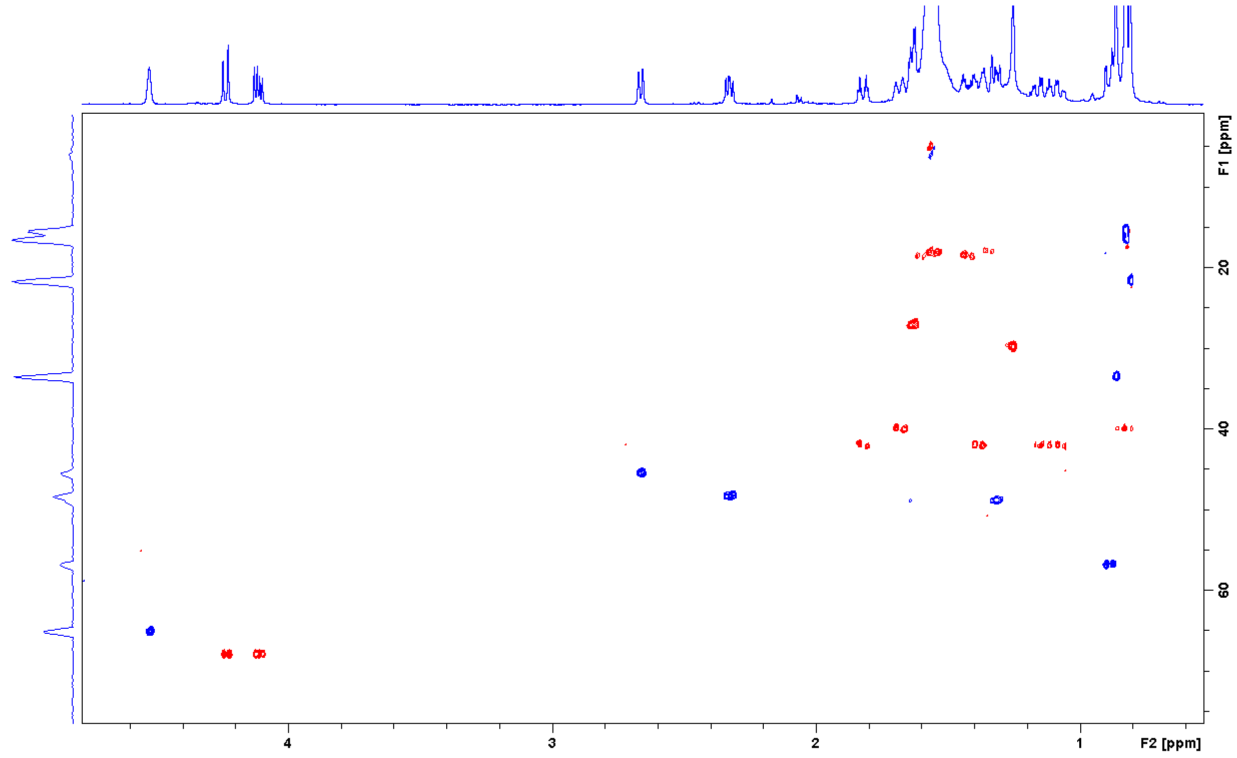

Figure S38. HSQC spectrum of **8** (500 MHz, $CDCl_3$).

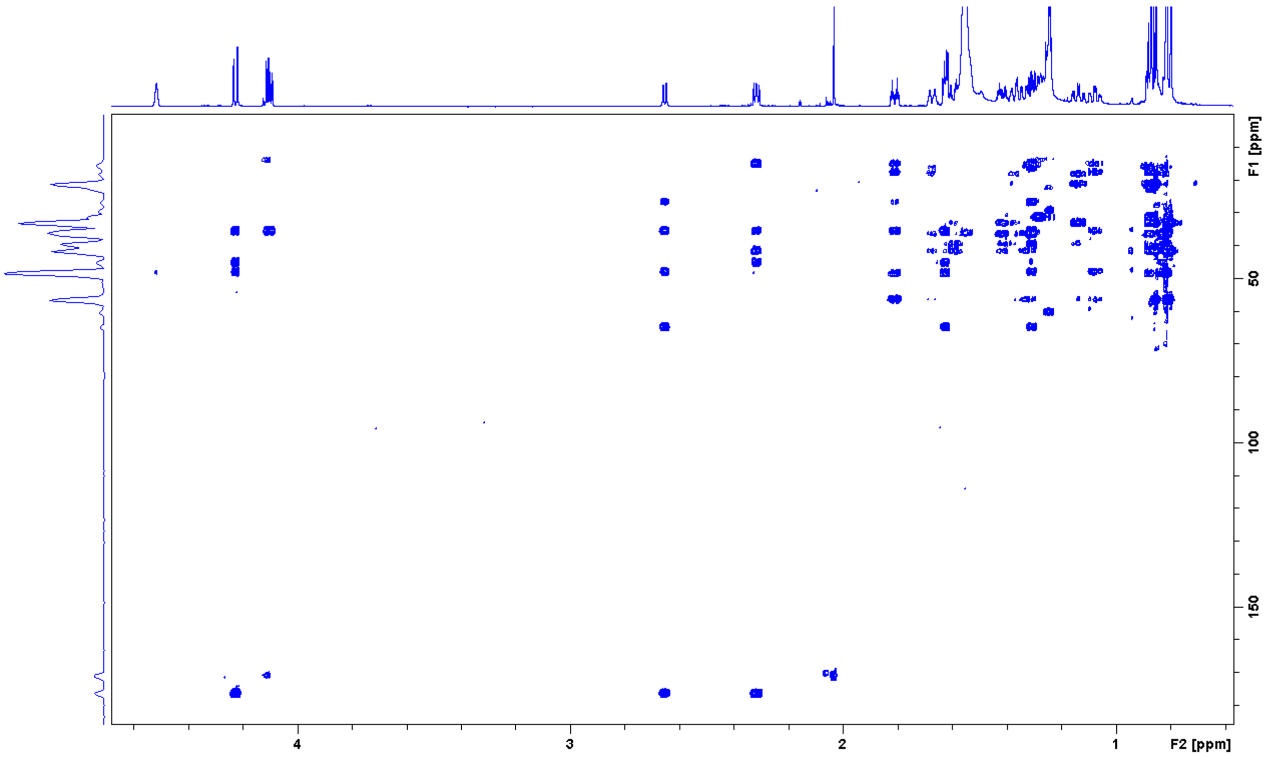

Figure S39. HMBC spectrum of **8** (700 MHz, $CDCl_3$).

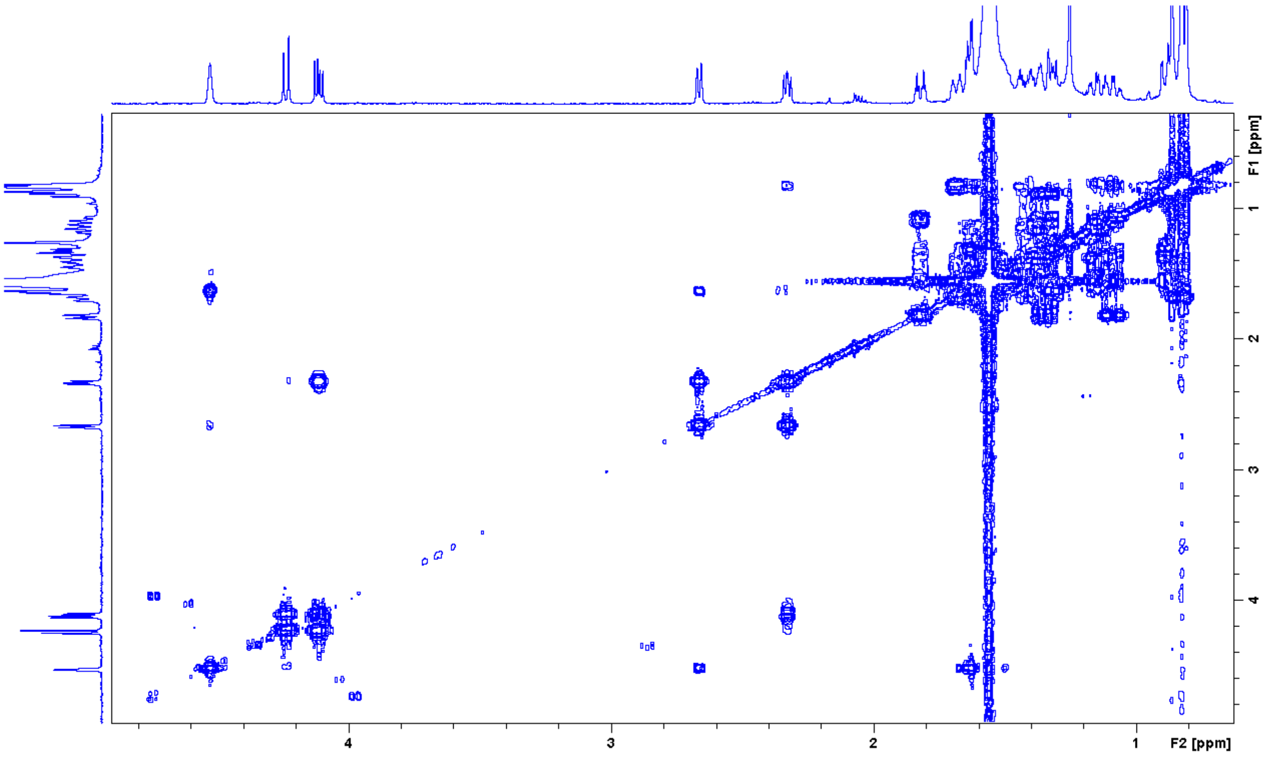

Figure S40. COSY spectrum of **8** (500 MHz, $CDCl_3$).

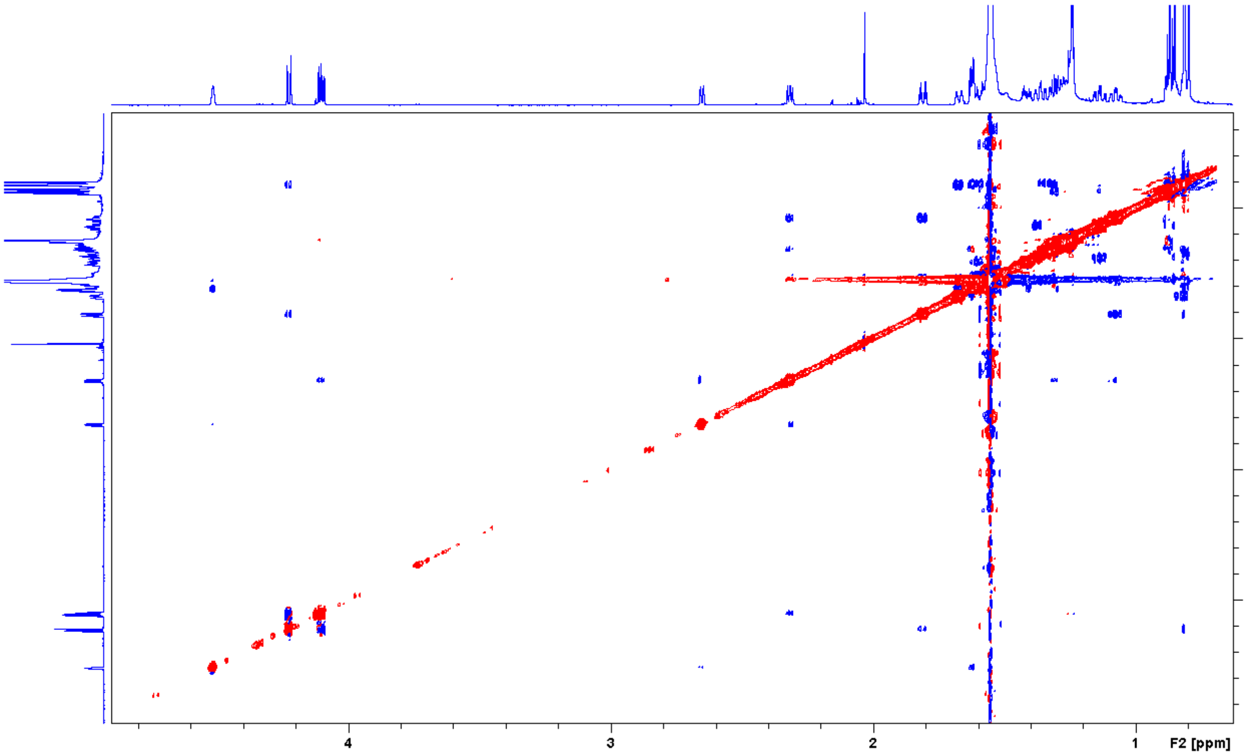

Figure S41. NOESY spectrum of **8** (700 MHz, $CDCl_3$).

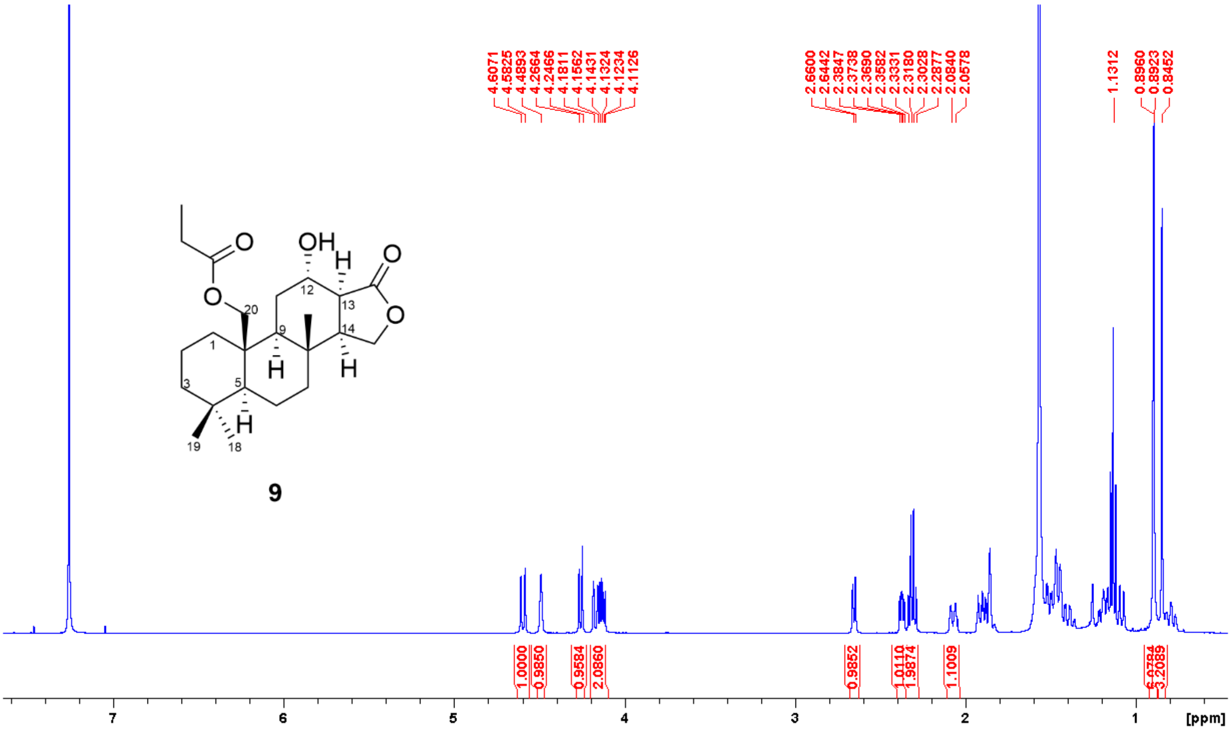

Figure S42. ^{1}H NMR spectrum of **9** (500 MHz, $CDCl_3$).

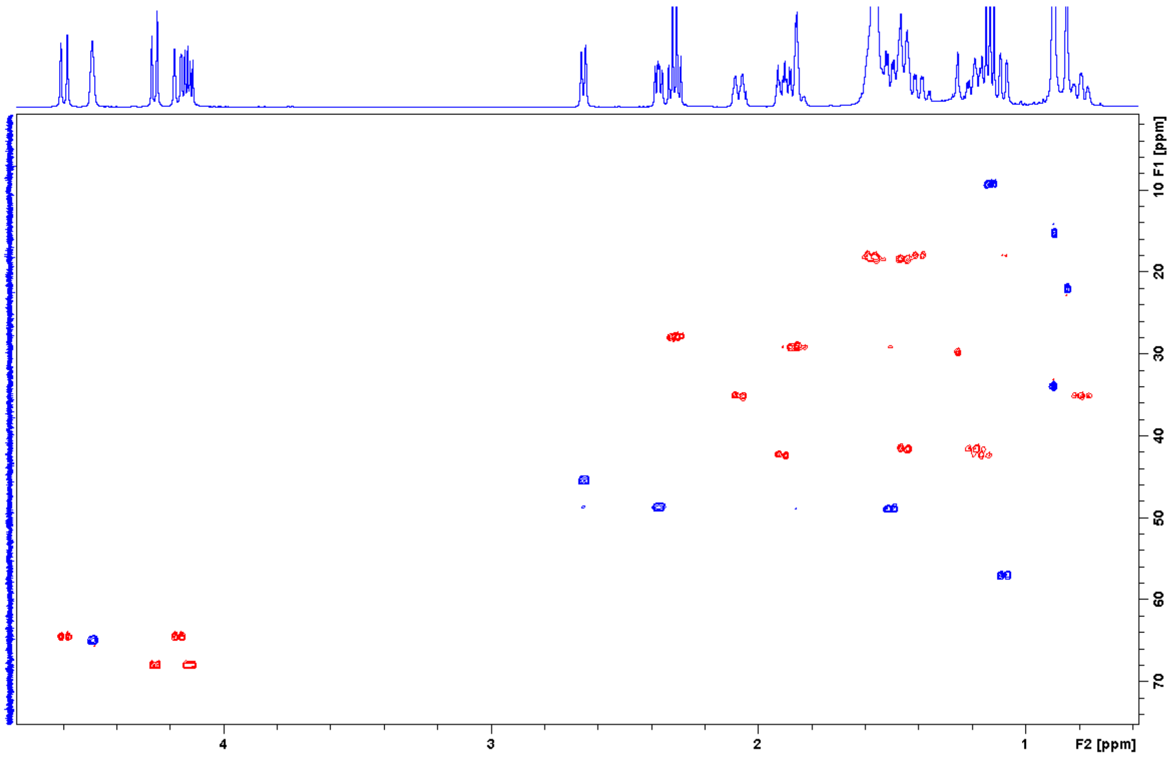

Figure S43. HSQC spectrum of **9** (500 MHz, $CDCl_3$).

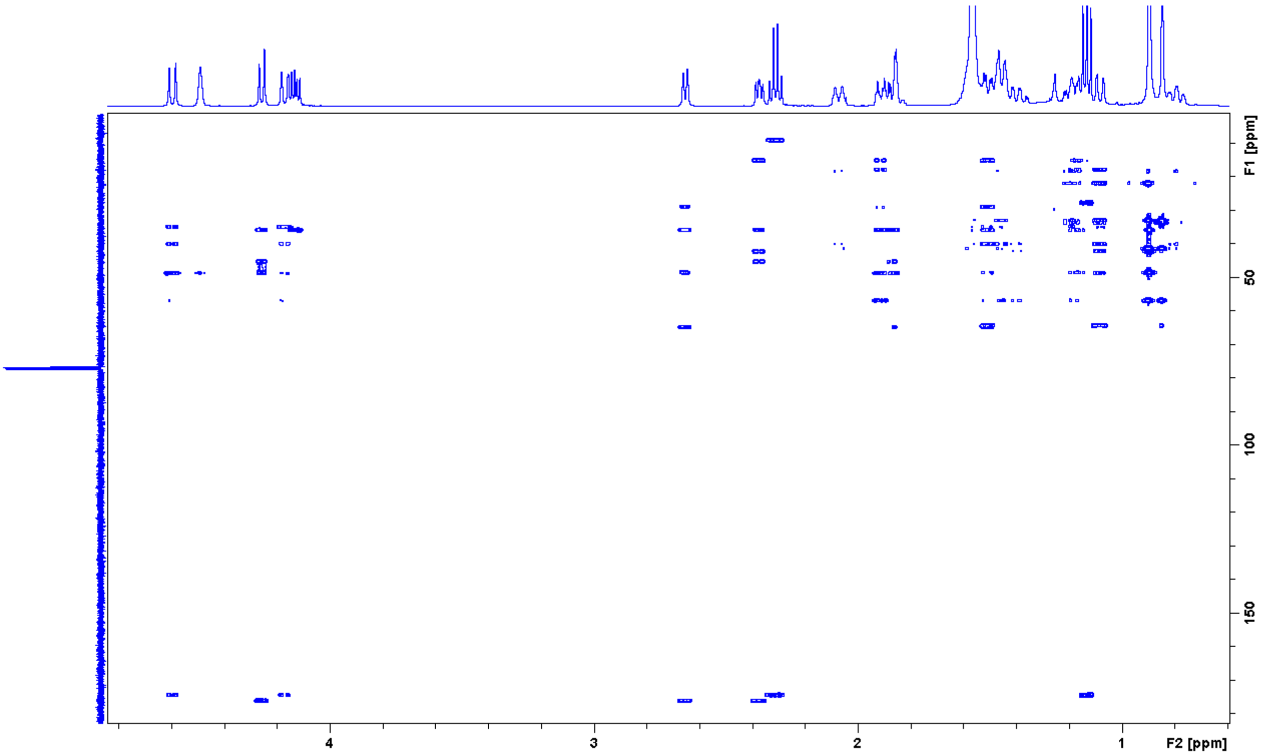

Figure S44. HMBC spectrum of **9** (500 MHz, $CDCl_3$).

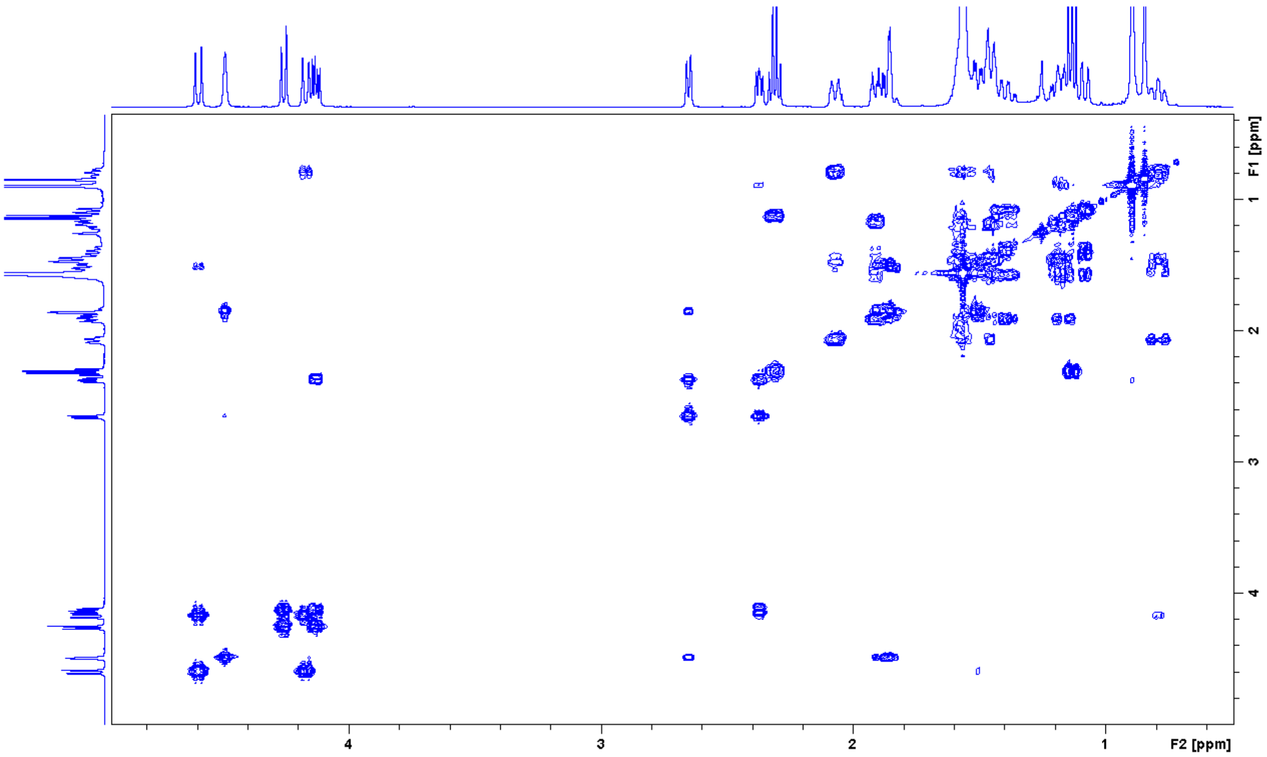

Figure S45. COSY spectrum of **9** (500 MHz, $CDCl_3$).

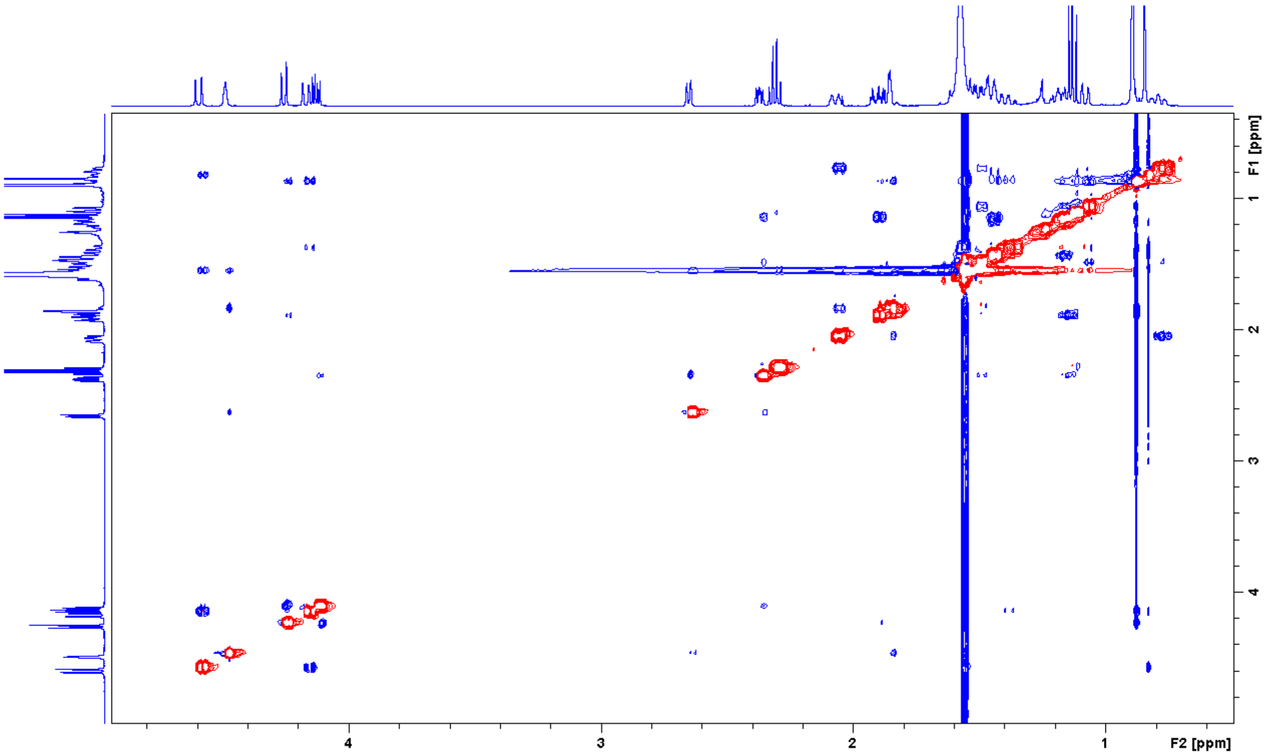

Figure S46. NOESY spectrum of **9** (500 MHz, $CDCl_3$).

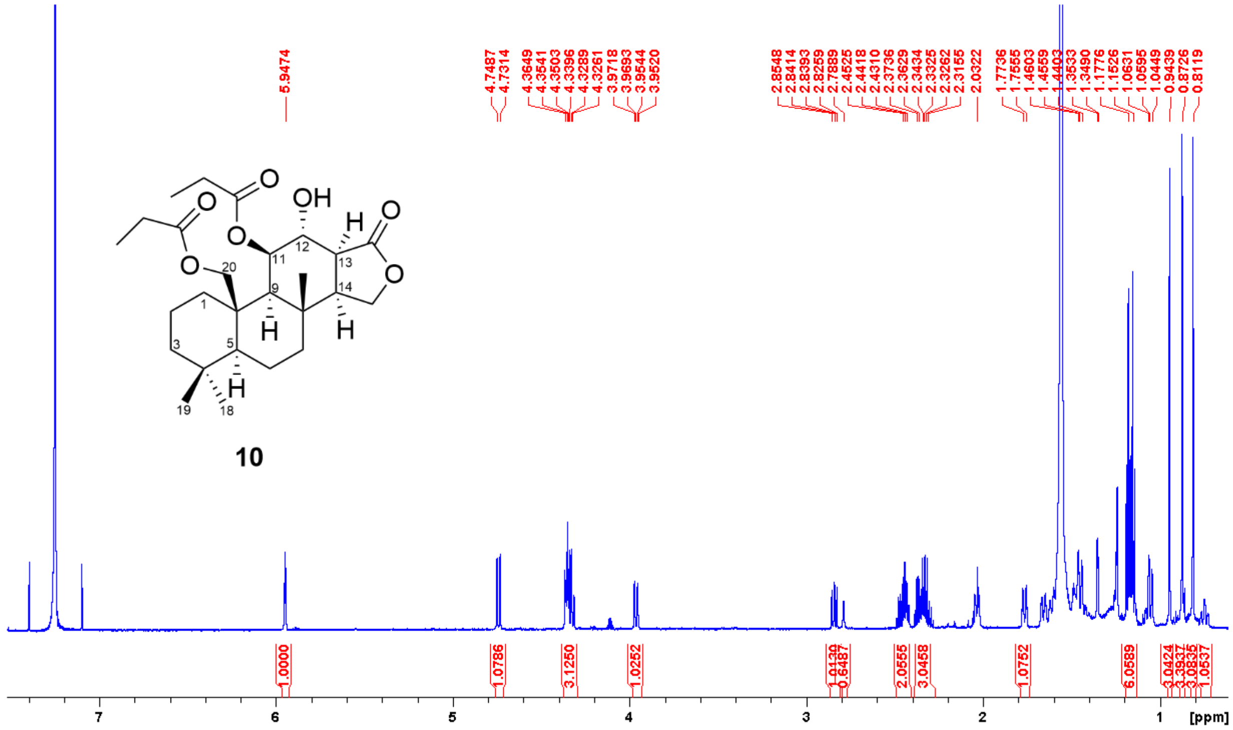

Figure S47. ^{1}H NMR spectrum of **10** (700 MHz, $CDCl_3$).

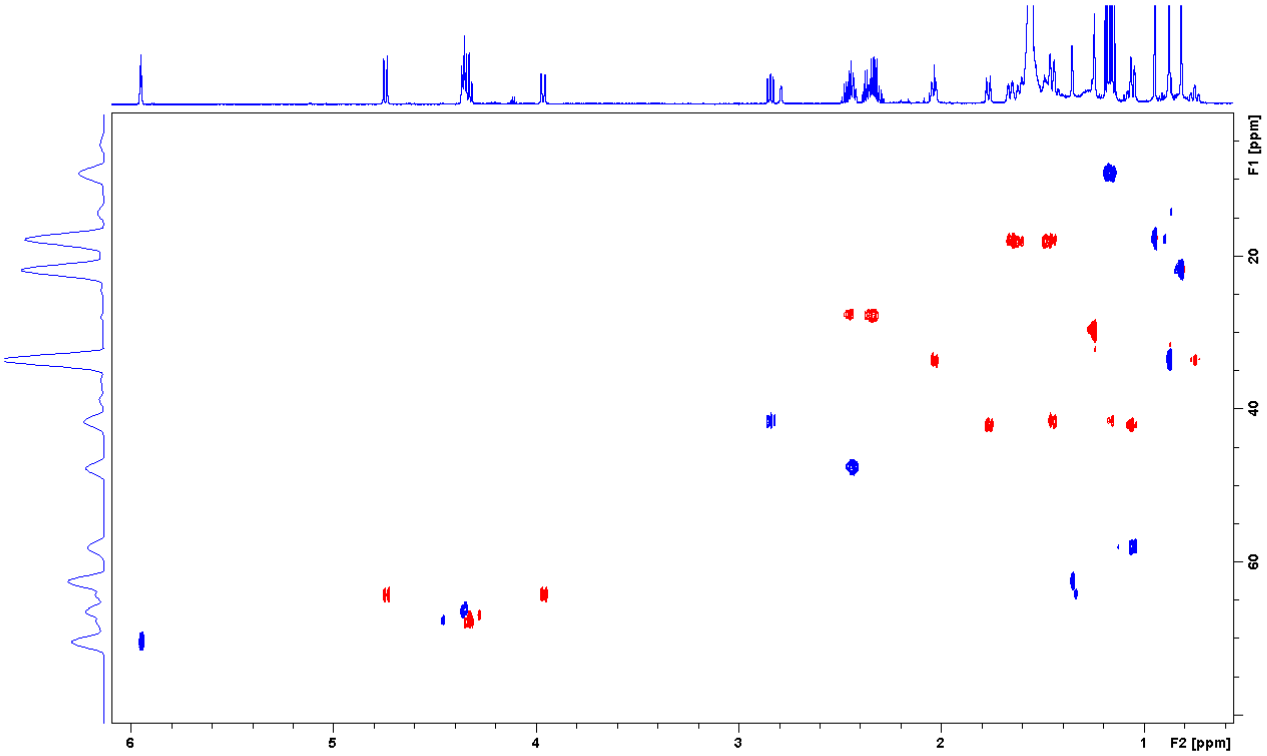

Figure S48. HSQC spectrum of **10** (700 MHz, $CDCl_3$).

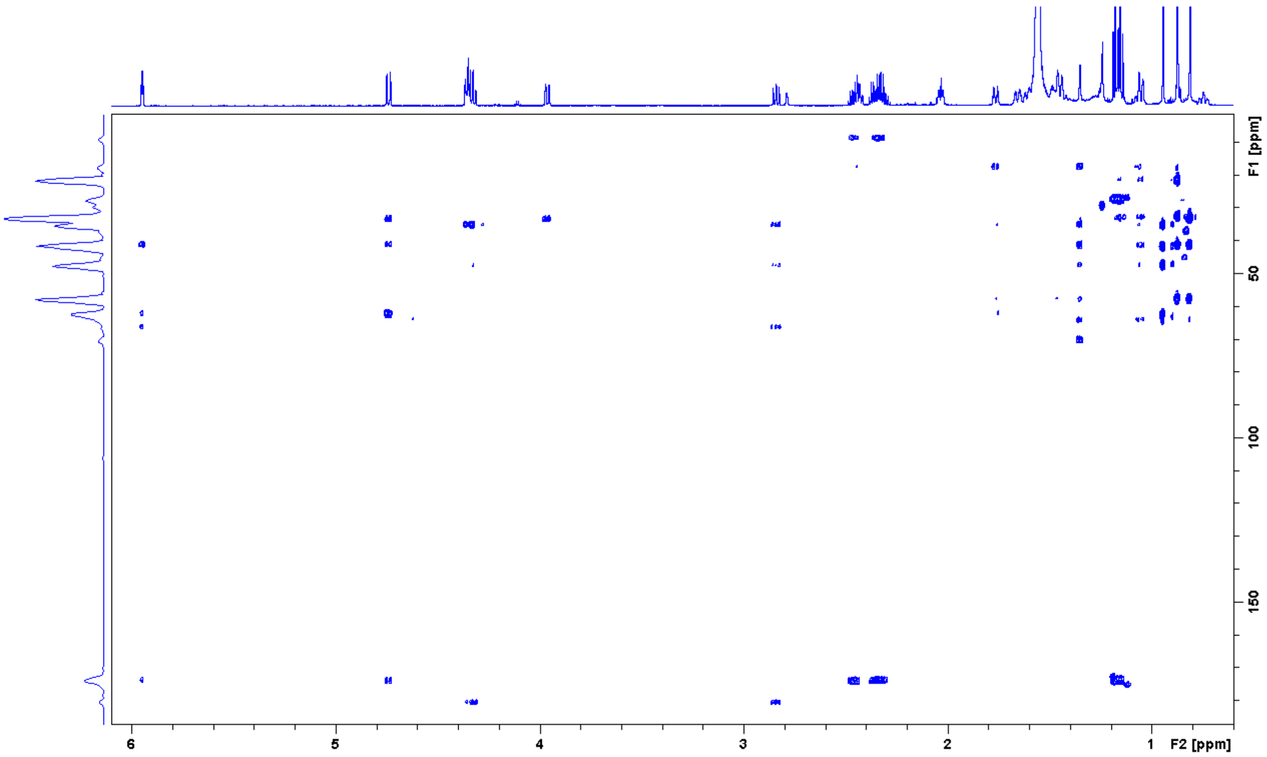

Figure S49. HMBC spectrum of **10** (700 MHz, $CDCl_3$).

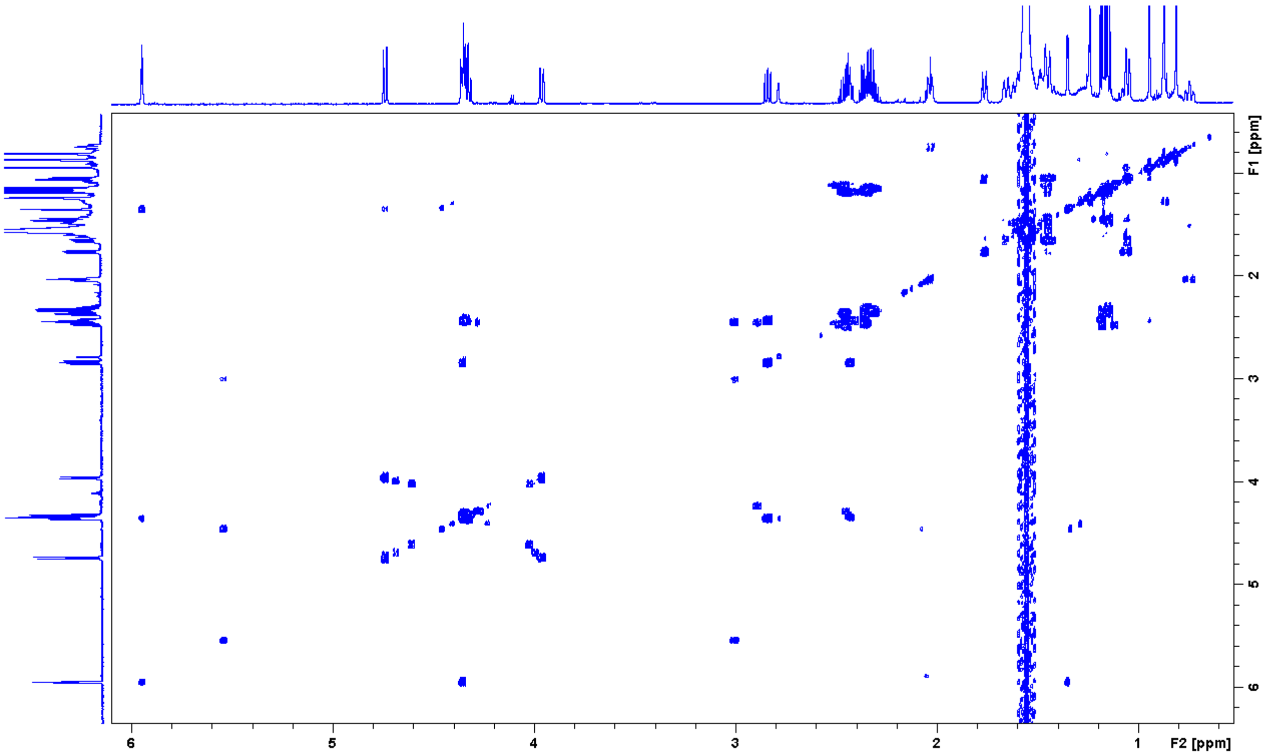

Figure S50. COSY spectrum of **10** (700 MHz, $CDCl_3$).

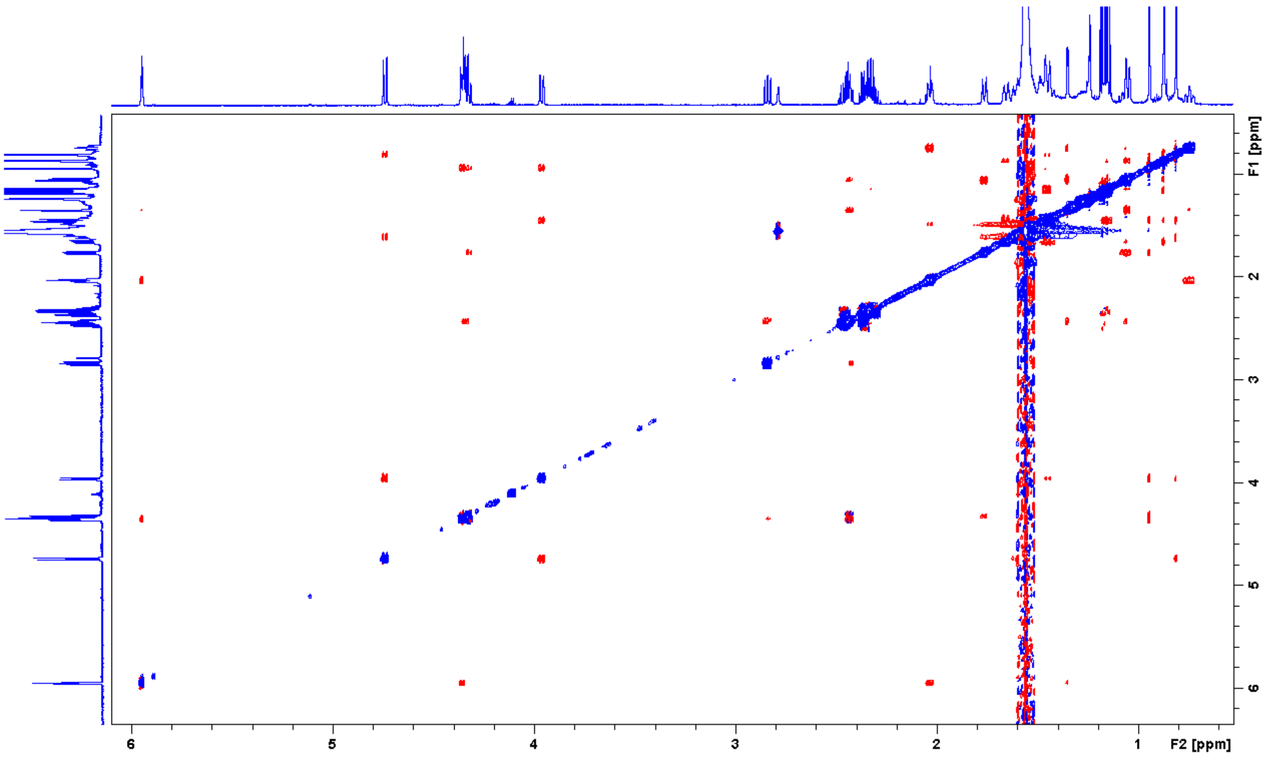

Figure S51. NOESY spectrum of **10** (700 MHz, $CDCl_3$).

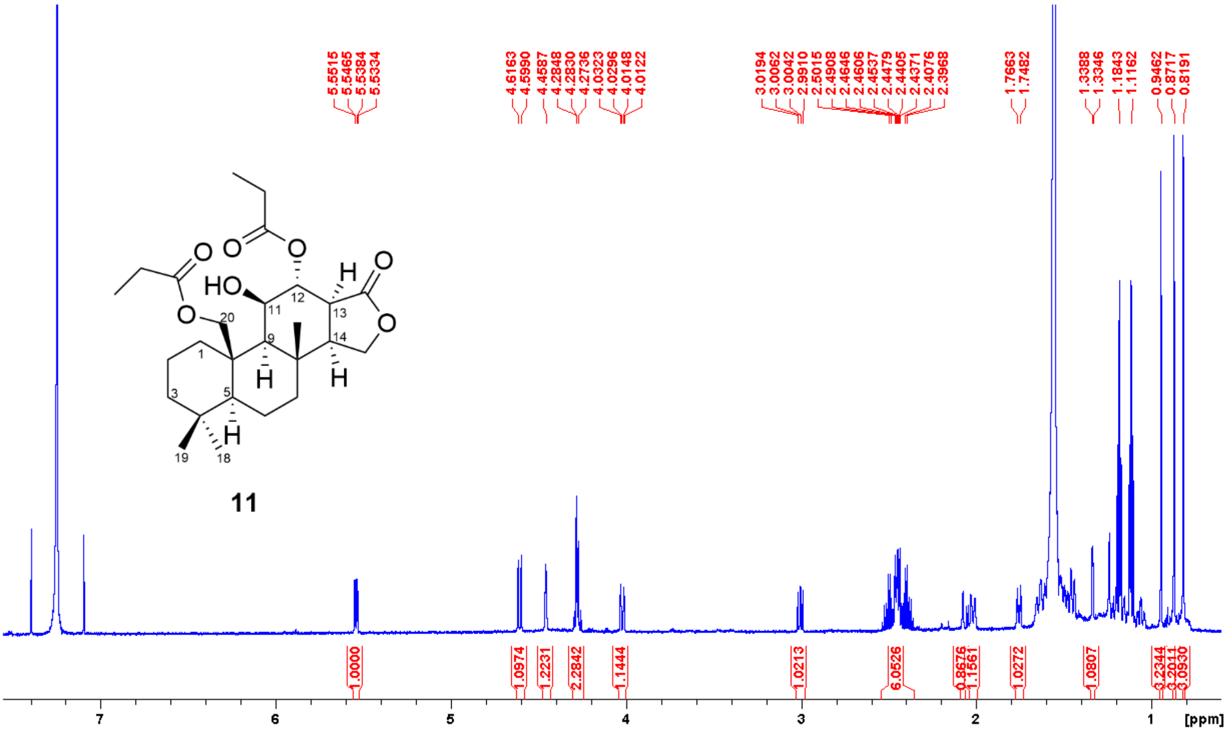

Figure S52. ^{1}H NMR spectrum of **11** (700 MHz, $CDCl_3$).

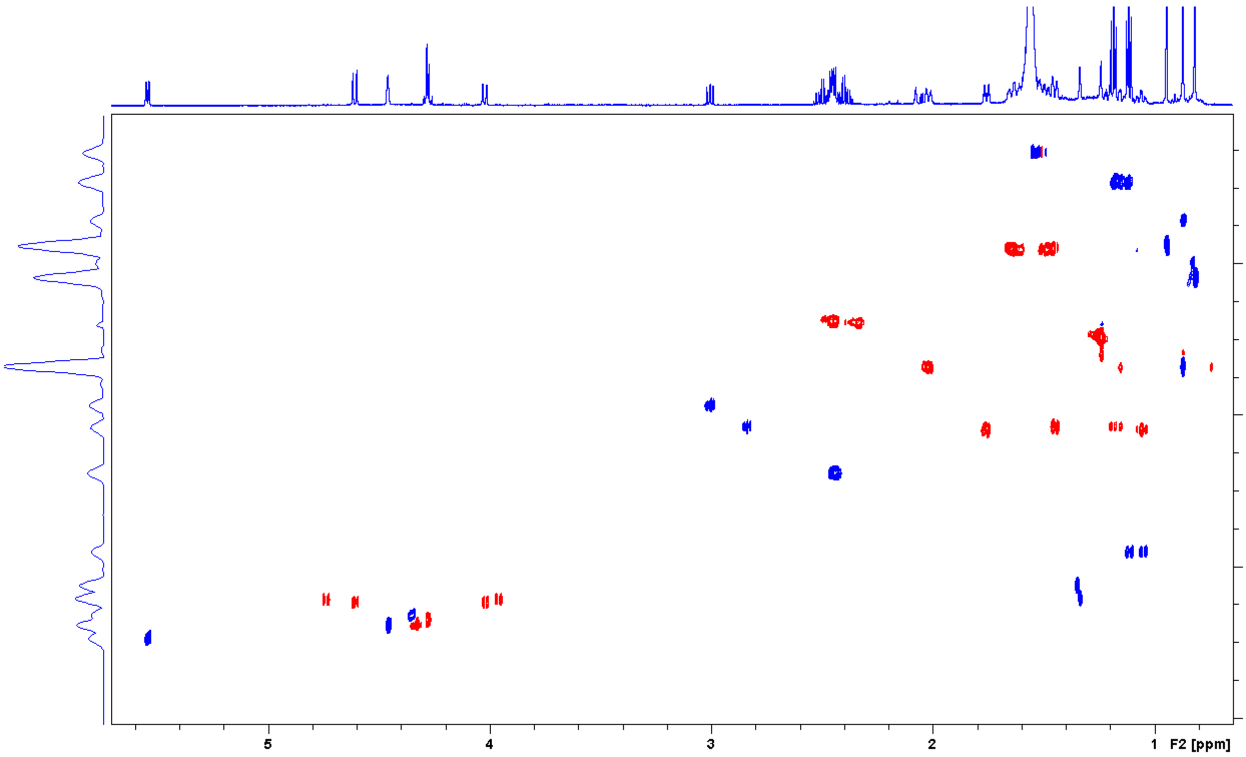

Figure S53. HSQC spectrum of **11** (700 MHz, $CDCl_3$).

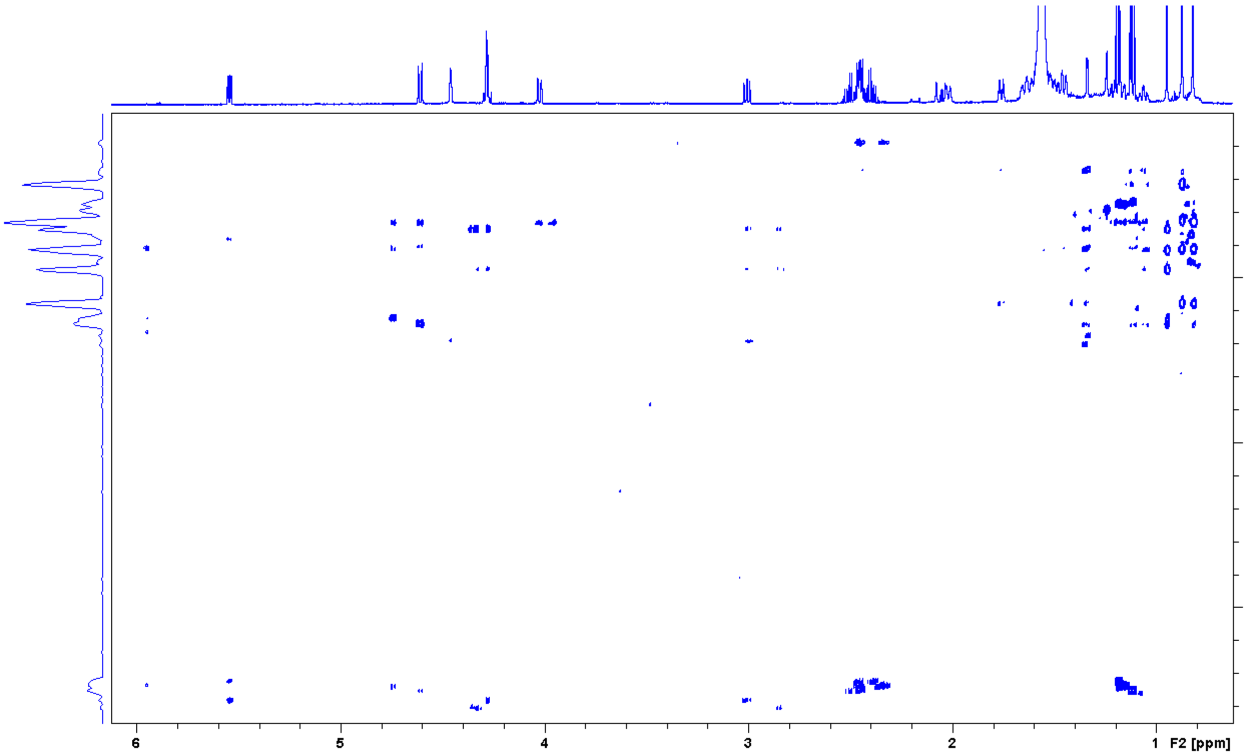

Figure S54. HMBC spectrum of **11** (700 MHz, $CDCl_3$).

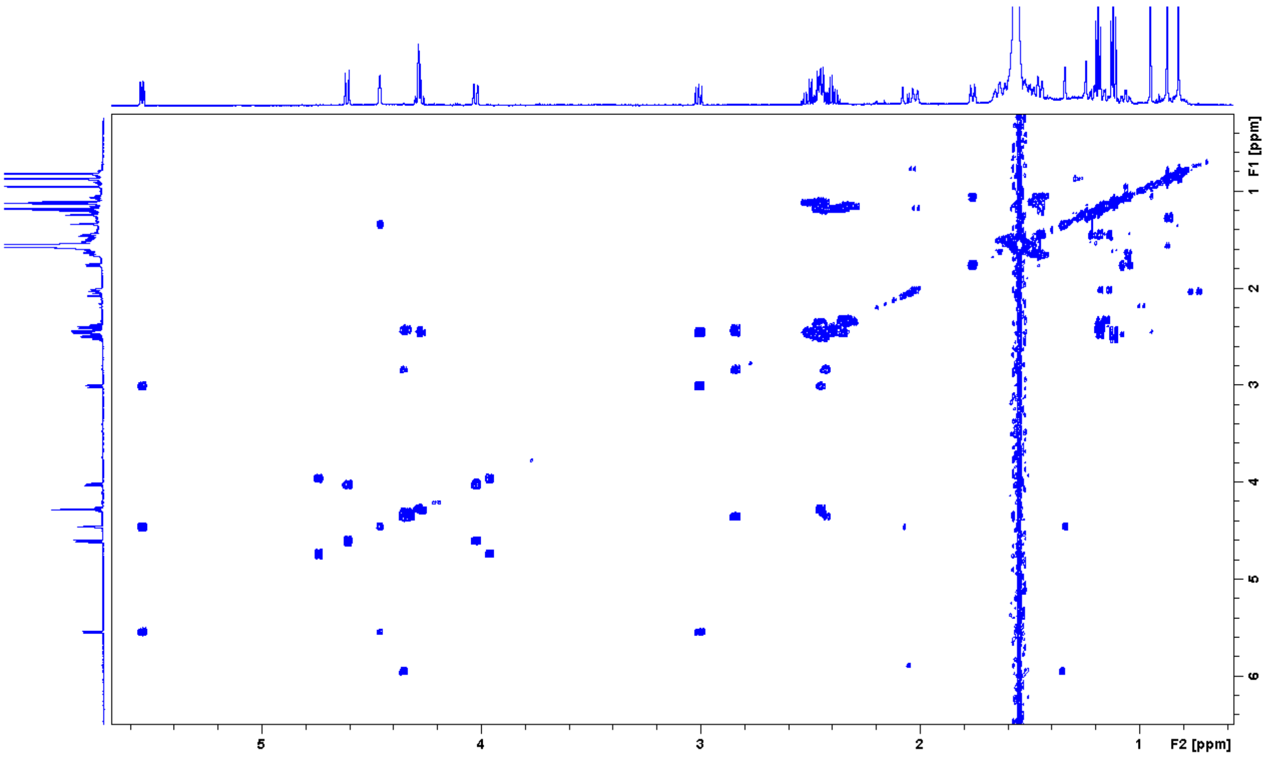

Figure S55. COSY spectrum of **11** (700 MHz, $CDCl_3$).

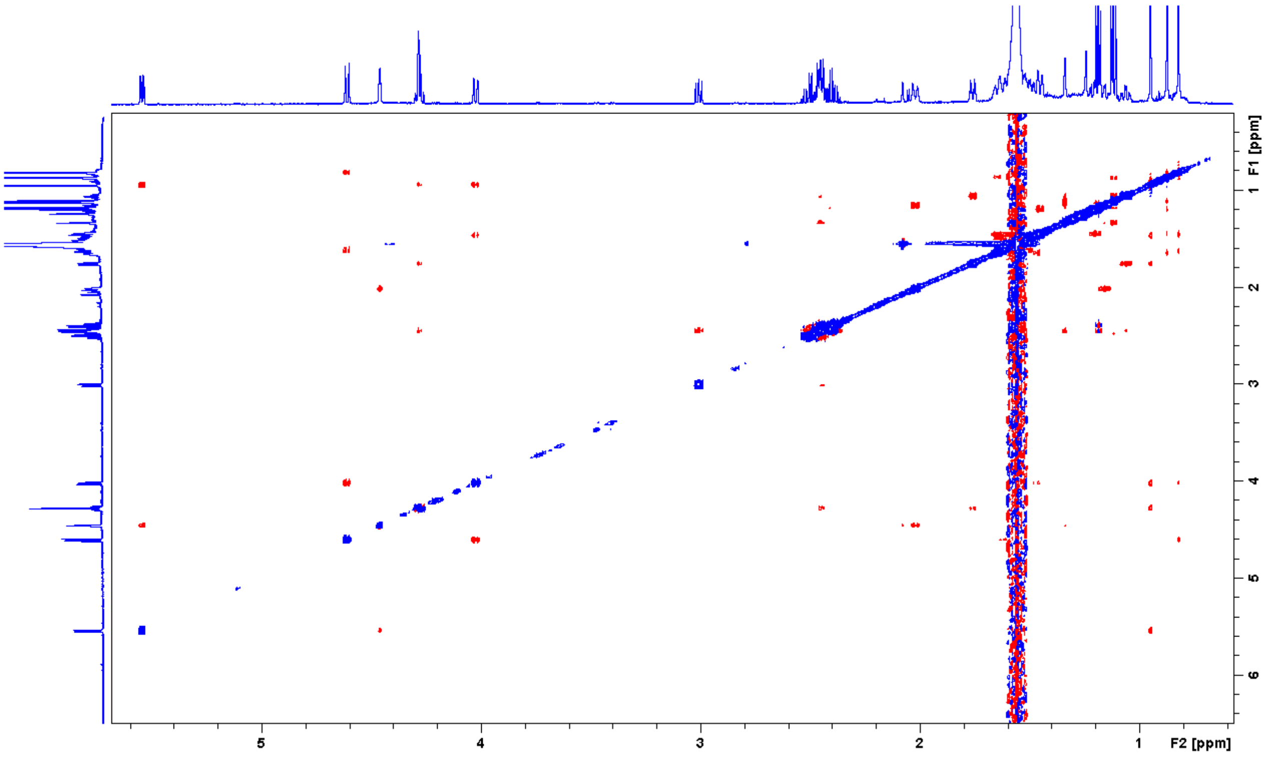

Figure S56. NOESY spectrum of **11** (700 MHz, $CDCl_3$).

Figure S57. Overlay of ^{1}H NMR spectra for the mantle of six specimens of *G. aureopurpureus* (500 MHz, $CDCl_3$).

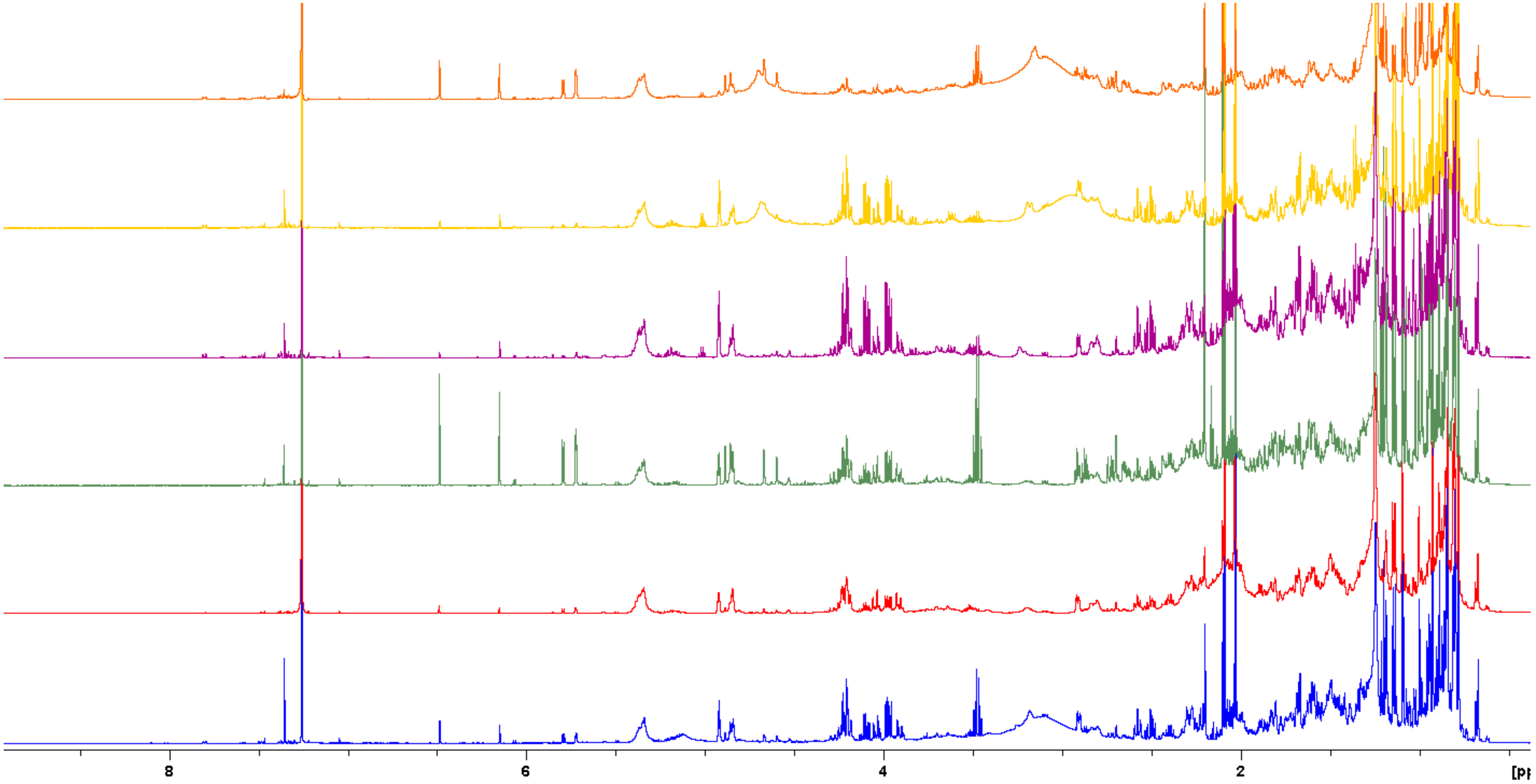

Figure S58. Overlay of ^{1}H NMR spectra for the viscera of six specimens of *G. aureopurpureus* (500 MHz, $CDCl_3$).

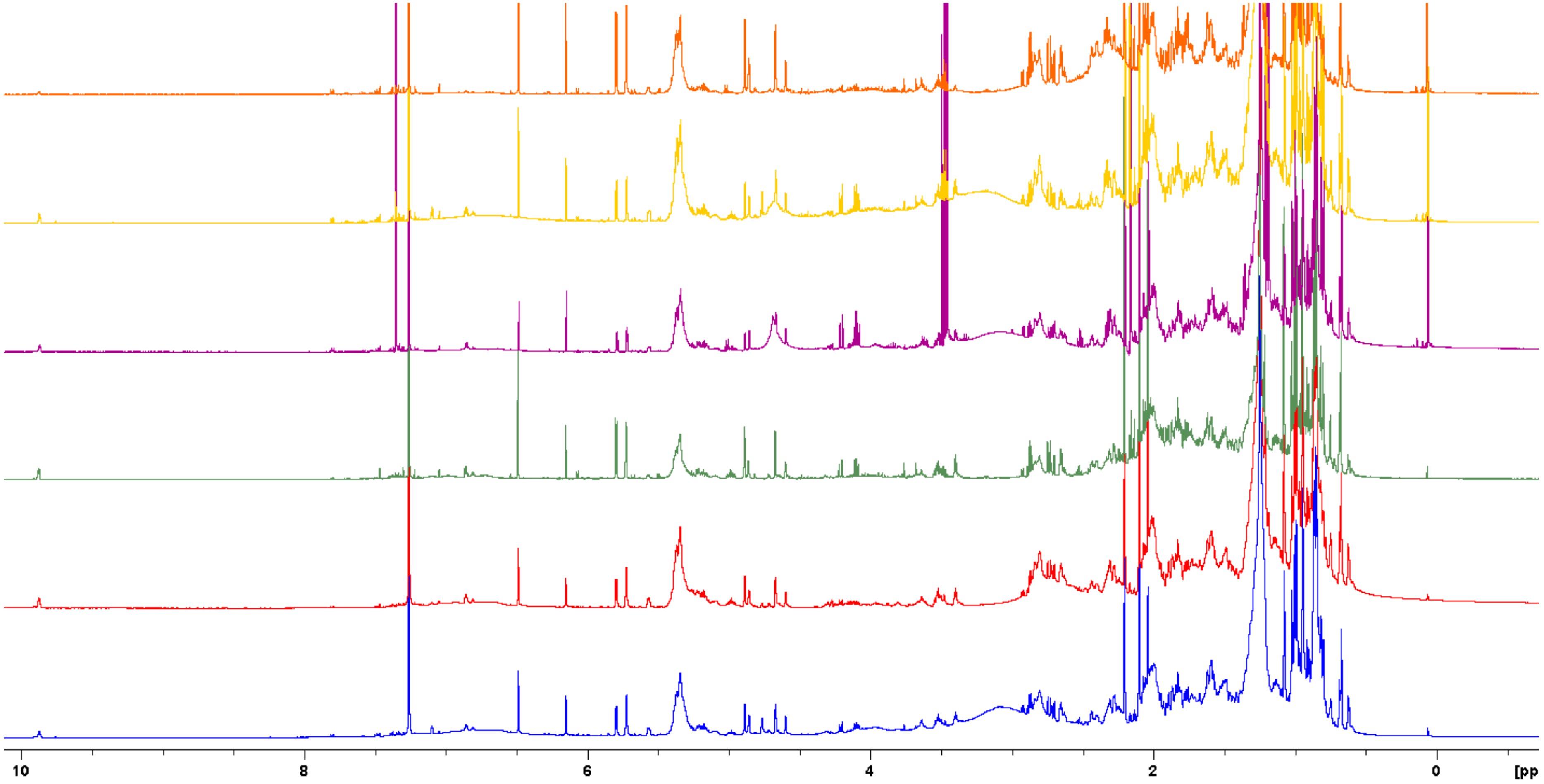

Figure S59. Overlay of ^{1}H NMR spectra for the mantle and viscera of *G. aureopurpureus* (500 MHz, $CDCl_3$).

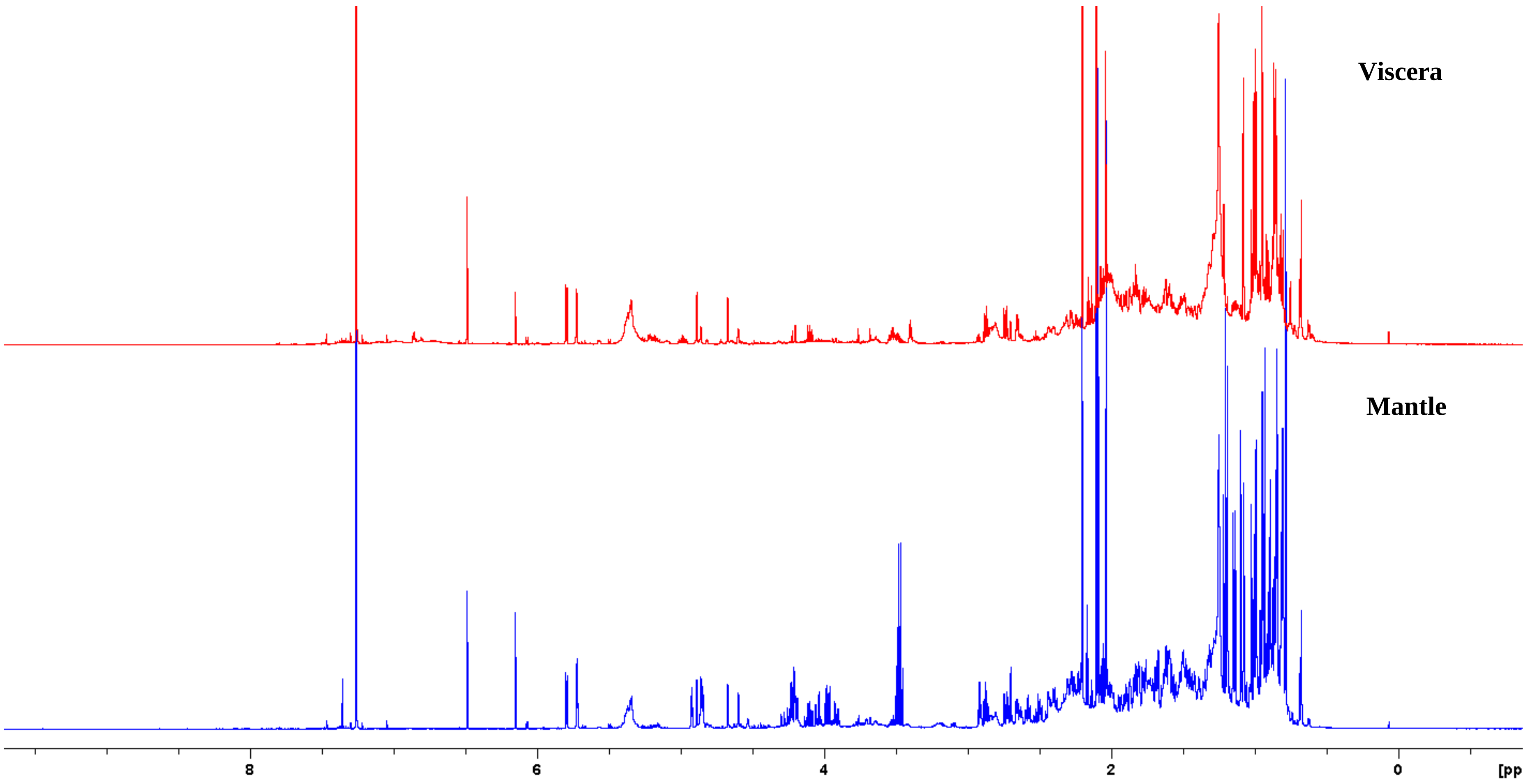

Figure S60. Overlay of ^{1}H NMR spectra for the mantle of three specimens of *Goniobranchus sp.1* (500 MHz, $CDCl_3$).

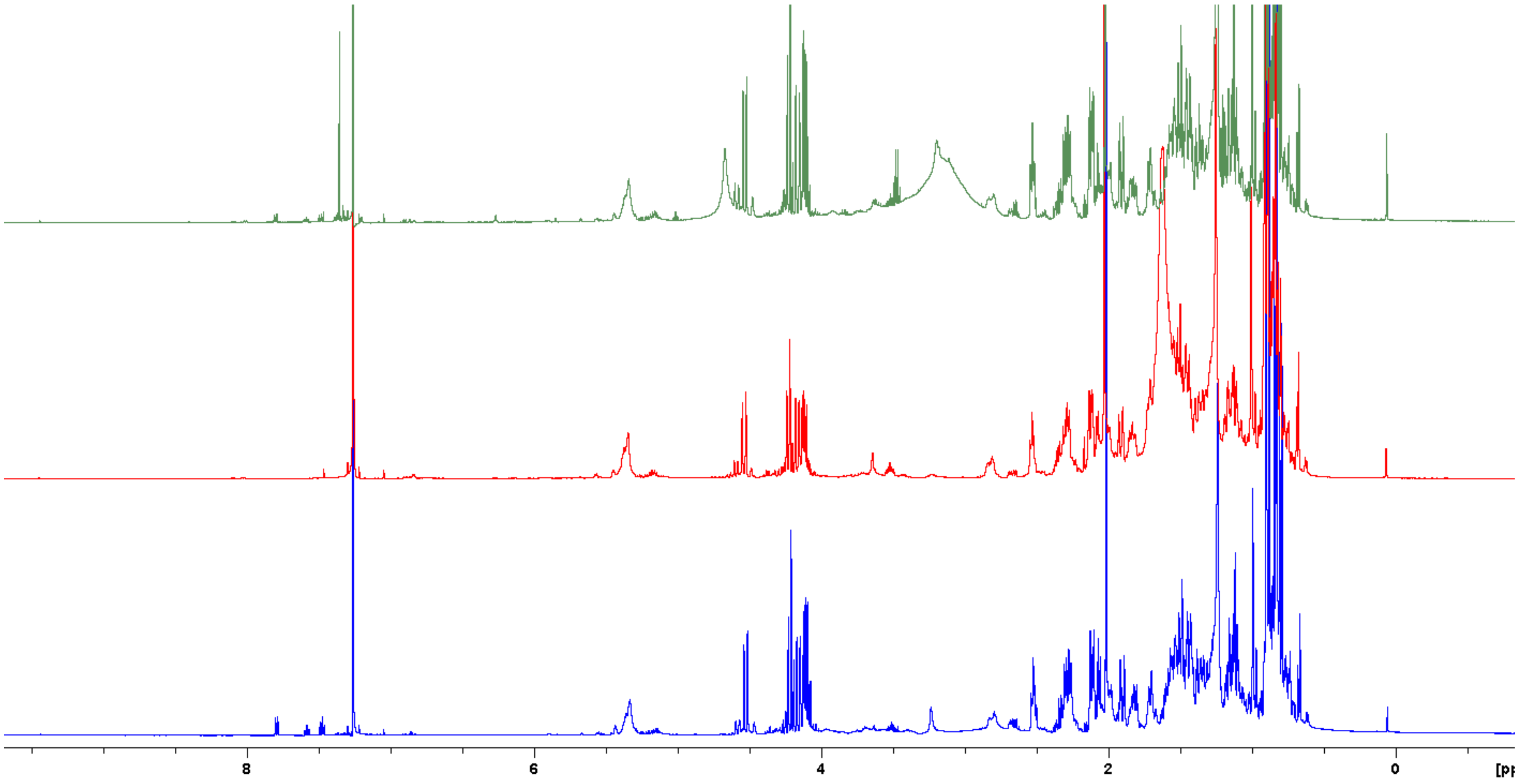

Figure S61. Overlay of ^{1}H NMR spectra for the viscera of three specimens of *Goniobranchus sp.1* (500 MHz, $CDCl_3$).

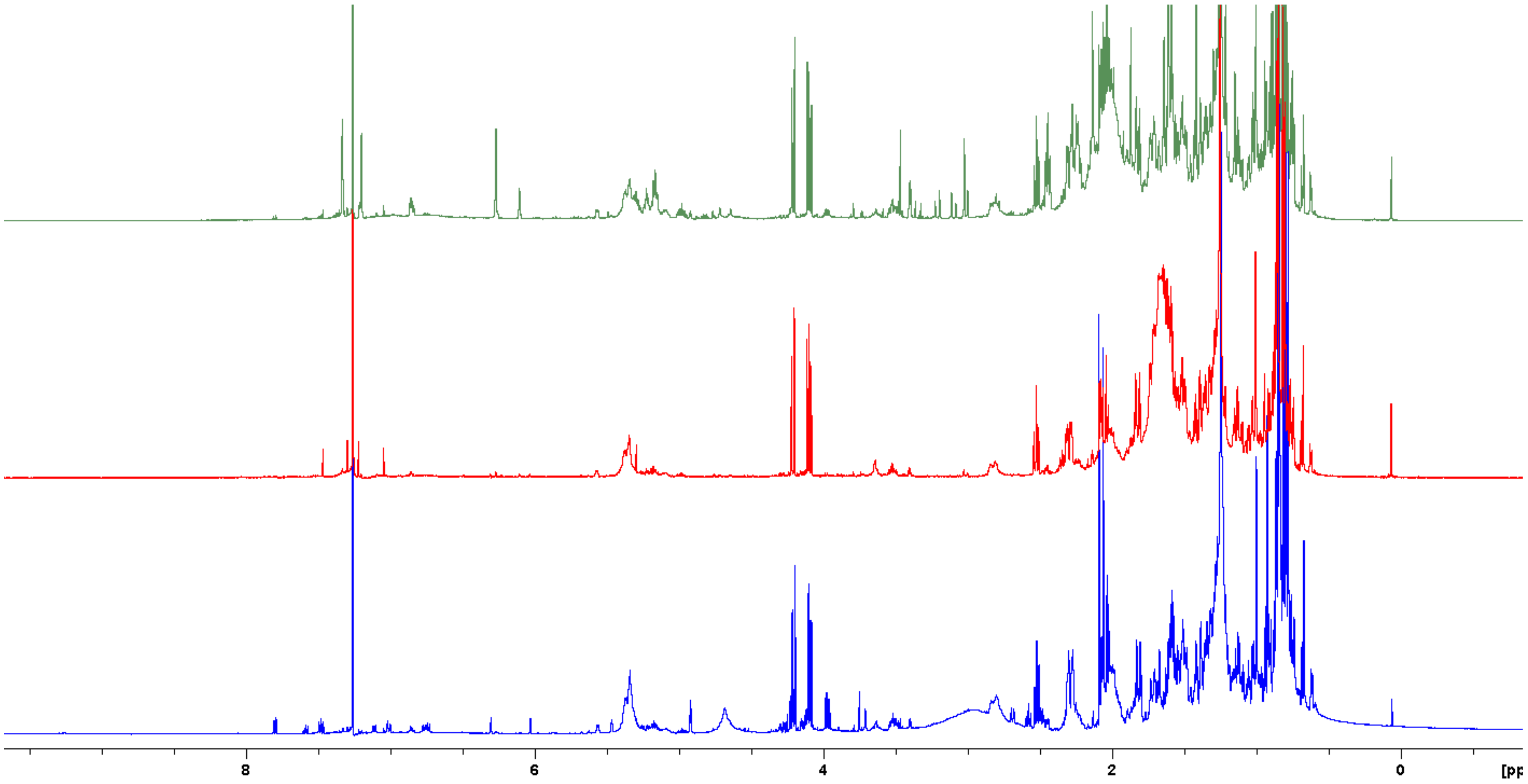

Figure S62. Overlay of ^{1}H NMR spectra for the mantle and viscera of *Goniobranchus sp.1* (500 MHz, $CDCl_3$).

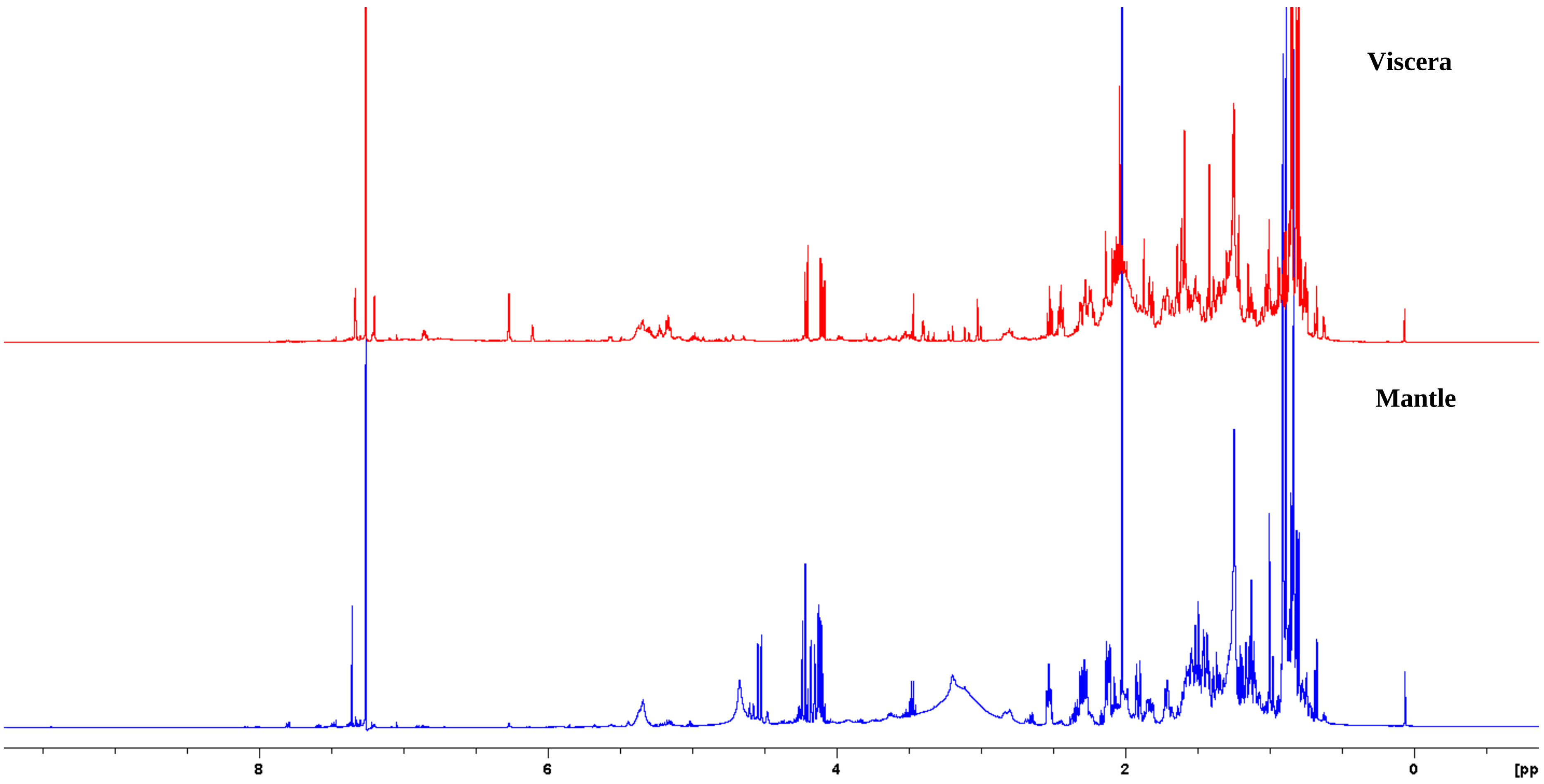

Supplement: Supplementary file 1 [file marinedrugs-19-00680-s001.zip › marinedrugs-1455637-supplementary.pdf]
